# Assessment of agronomic traits in fall armyworm (*Spodoptera frugiperda*) resistant maize (*Zea mays*) hybrids grown in the derived savanna agro-ecology of Nigeria

**DOI:** 10.1371/journal.pone.0352140

**Published:** 2026-06-26

**Authors:** Olufemi Stephen Akande, Adesike Oladoyin Olayinka, Moses Adeolu Adebayo

**Affiliations:** 1 Crop Production and Soil Science Department, Ladoke Akintola University of Technology, Ogbomoso, Oyo State, Nigeria; 2 Department of Crop and Animal Science, Ajayi Crowther University, Oyo, Oyo State, Nigeria; National Cheng Kung University, TAIWAN

## Abstract

Maize production in Nigeria has increased in recent years, but fall armyworm infestation remains a major constraint to high productivity, particularly during the minor cropping season. Twenty-six fall armyworm-resistant maize hybrids and six commercial checks were evaluated for grain yield and other traits using an 8 × 4 alpha-lattice design with three replications. Data collected at key growth stages and harvesting were analyzed using R statistical software version 4.4.2. Hybrids exhibited significant (p < 0.05) differences for grain yield, plant aspect, ear aspect, southern corn leaf blight, *curvularia* leaf spot and foliar fall armyworm damage at 8 and 12 weeks after planting. Notably, five experimental hybrids exhibited superior performance, combining high yield with desirable agronomic traits and low foliar fall armyworm damage. FAWSYN-1/(TZLComp. 1 C6-W-39-1-1)-B-B, followed closely by FAWSYN-1/IITATZI2305, FAWSYN-2/(TZLComp. 1 C6-W-39-1-1)-B-B, FAWSYN-1/TZISTR1121, and FAWSYN-3/IITATZI2305 exhibited the highest superiority indices across environments. When further tested for performance stability across more diverse agro-ecologies, the selected hybrids may be released as maize cultivars to mitigate fall armyworm attacks among Nigerian farmers.

## Introduction

Maize (*Zea mays*) is one of the most widely cultivated and important cereal crops globally, serving as a staple food for millions of people and as a vital component of animal feed, forage and industrial applications [1,2]. The annual global production has surpassed 1.147 billion tons [3]. Its adaptability to diverse agro-climatic conditions enables cultivation across various regions, with leading producers being the United States, China, Brazil, Argentina, and India [4]. However, achieving consistent productivity depends on the development and systematic evaluation of improved hybrids, as grain yield and related agronomic traits can vary significantly among genotypes and across growing seasons, particularly under rainfed conditions [2]. It remained a key source of food and economic livelihoods for approximately 300 million smallholder farmers across sub-Saharan Africa (SSA) [5].

Maize production in Nigeria reached 12.75 million metric tons in 2021 [6]. Despite its critical role in the country, the crop faces multiple constraints, particularly biotic stresses, including insect pests and diseases, which cause significant yield reductions [7]. Among these, *Spodoptera frugiperda,* commonly referred to as FAW, an invasive polyphagous pest, has become a major threat since its detection in West and Central Africa (WCA) in 2016 [8]. The pest feeds voraciously on maize leaves, stalks, tassels, and ears, leading to weakened plants, increased vulnerability to secondary infections, and substantial yield losses [9]. FAW infestations have been associated with annual economic losses estimated at US$ 2.5–6.2 billion in twelve affected African countries, and up to US$ 13 billion in maize, rice, sorghum, and sugarcane across SSA [10,11]. For instance, Ethiopia experienced a 36% average annual yield loss between 2017 and 2019, attributed to the pest, resulting in an economic loss of US$ 200 million [12]. Similarly, maize yields in Nigeria declined by over 40% due to foliar FAW damage [13].

The invasion of FAW in Africa has led to increased pesticide use among smallholder farmers, raising concerns about environmental and health risks. Studies have shown that synthetic pesticides remain the most popular management option, with usage increasing up to 3-fold in some areas [14]. However, the calamitous environmental impacts of pesticide use, coupled with the rising costs of the chemicals, which are becoming increasingly unaffordable by resource-poor farmers across SSA, have rendered this strategy unattractive [15,16]. Breeding maize hybrids with enhanced resistance or tolerance to FAW has emerged as a sustainable strategy to mitigate crop losses and reduce reliance on pesticides [17,18]. Institutions such as the International Institute of Tropical Agriculture (IITA) and the International Maize and Wheat Improvement Centre (CIMMYT) have made substantial progress in developing FAW-resistant maize varieties using a combination of traditional breeding and molecular tools [19].

The derived savanna agro-ecology of southwestern Nigeria is an important maize-growing region characterized by bimodal rainfall patterns and moderate temperatures conducive to maize cultivation [2]. However, its climatic conditions also favor FAW resurgence and proliferation, posing significant challenges to maize productivity in the zone [20]. While several resistant FAW maize hybrids have been developed and evaluated, their performance and adaptability within the derived savanna agro-ecology of Nigeria remain understudied [21–23]. Systematic evaluation of these hybrids for yield, pest resistance, and agronomic traits is essential to guide the identification and selection of superior varieties. Thus, the objectives of this study were to: (i) assess the agronomic performance and variation among new, IITA-developed FAW-resistant maize hybrids in the minor cropping season, (ii) determine the association between grain yield and other desirable agronomic traits, (iii) identify key traits influencing hybrid performance, and (iv) select superior maize hybrids with enhanced yield and FAW tolerance.

## Materials and methods

### Genetic materials, experimental site, and field establishment

The genetic materials evaluated in this study consisted of 26 FAW-resistant maize hybrids and 6 commercial checks obtained from the International Institute of Tropical Agriculture (IITA), Ibadan, Nigeria (Table 1). The 26 experimental hybrids represent genetically diverse materials bred specifically for tolerance or resistance to FAW. Field trials were conducted in 2024 at two environments within the derived savanna agro-ecology of southwestern Nigeria at the Teaching and Research Farm of Ladoke Akintola University of Technology (LAUTECH), Ogbomoso (8.172°N, 4.274°E), and the Teaching and Research Farm of Ajayi Crowther University (ACU), Oyo (7.734°N, 4.064°E).

**Table 1:** List of the 26 FAW-resistant synthetic top-cross maize hybrids and 6 commercial hybrid checks evaluated in two locations in the derived savanna agro-ecology of Nigeria.

Hybrid ID	Pedigree
**G1**	FAWSYN-1/(TZLComp. 1 C6-W-39-1-1)-B-B
**G2**	FAWSYN-1/TZISTR1869
**G3**	FAWSYN-1/TZISTR1878
**G4**	FAWSYN-1/TZISTR1121
**G5**	FAWSYN-1/TZISTR1129
**G6**	FAWSYN-1/TZISTR2024
**G7**	FAWSYN-1/TZISTR1305
**G8**	FAWSYN-1/TZISTR2042
**G9**	FAWSYN-2/(TZLComp. 1 C6-W-39-1-1)-B-B
**G10**	FAWSYN-2/TZISTR1878
**G11**	FAWSYN-2/TZISTR1129
**G12**	FAWSYN-2/TZISTR2024
**G13**	FAWSYN-2/TZISTR1305
**G14**	FAWSYN-2/TZISTR2129–2
**G15**	FAWSYN-3/TZISTR1869
**G16**	FAWSYN-3/TZISTR1872
**G17**	FAWSYN-3/TZISTR1878
**G18**	FAWSYN-3/IITATZI2300
**G19**	FAWSYN-3/IITATZI2305
**G20**	FAWSYN-1/IITATZI2300
**G21**	FAWSYN-2/IITATZI2300
**G22**	FAWSYN-1/IITATZI2305
**G23**	FAWSYN-2/IITATZI2305
**G24**	FAWSYN-1
**G25**	FAWSYN-2
**G26**	FAWSYN-3
**G27**	SAMMAZ 51
**G28**	Oba Super 11
**G29**	SC301
**G30**	Oba Super 7
**G31**	Oba Super 9
**G32**	Oba Super II

Both environments experience bimodal rainfall, with average annual precipitation ranging from 1,000–1,200 mm and mean temperatures of 28 °C to 30 °C during the major and minor maize-growing seasons [24]. These climatic conditions, typical of the derived savanna agro-ecological zone, provide an ideal environment for assessing the agronomic performance and pest resistance of maize hybrids under natural FAW pressure.

The experimental field at each location was ploughed and harrowed two weeks before planting to optimize soil conditions. Experimental hybrids and checks were planted in July 2024 during the minor cropping season. Entries were laid out in an 8 × 4 alpha-lattice design with three replicates. Each plot was 4 m in length with inter-row spacing of 0.75 m and intra-row spacing of 0.5 m. Three seeds were planted in each hill, and the plants were thinned to two per stand, 2 weeks after planting (WAP) to attain a plant population density of 53,333 plants/ha. Standard agronomic practices, including fertilizer application and weed control, were done to ensure optimal crop growth. No pesticides were used to control FAW infestation throughout each experiment.

### Data collection

Data on key agronomic traits, foliar diseases (maize streak virus, southern corn leaf rust, southern corn leaf blight, and curvularia leaf spot), and foliar fall armyworm damage were recorded at 4, 8, and 12 weeks after planting and at harvest. These assessments were conducted in accordance with standard protocols for maize field trials, using a 1–5 scale for diseases and a modified Davis 1–9 scale for fall armyworm damage [15,25]. Phenological traits, including days to pollen shed and days to silking, were recorded as the number of days from planting to when 50% of plants in each plot began shedding pollen and emerged with receptive silks, respectively. The anthesis-silking interval was calculated by subtracting the number of days to pollen from the number of days to silking, providing insight into flowering synchrony, which affects grain yield [25]. Plant and ear heights were measured using a meter rule from the base of the plant to the first tassel branch and to the first node bearing the upper ear, respectively. Measurements were taken on ten representative plants per plot to calculate average values. Plant aspect was rated visually on a scale of 1–5, where 1 = excellent overall phenotypic appearance and 5 = poor overall phenotypic appearance. Ear aspect was also scored visually on a scale of 1–5, where 1 = clean, uniform, large, and well-filled ears, and 5 = rotten, variable, small, and partially filled ears. Husk cover was assessed on a scale of 1–5, where 1 = husks tightly arranged and extended beyond the ear tip, and 5 = ear tips fully exposed. Ear rot was evaluated using a scale of 1–5, with 1 indicating no rot and 5 severe infection [22]. Foliar diseases evaluated include maize streak virus (MSV), southern corn leaf rust (SCLR), southern corn leaf blight (SCLB), and *curvularia* leaf spot (CLS). Each disease was scored on a 1–5 scale based on symptom severity, with 1 indicating no visible symptoms and 5 indicating a severe infection [15]. Foliar FAW damage was assessed at 4, 8, and 12 weeks after planting (WAP) using a modified Davis scale ranging between 1 (no visible damage) and 9 (severe damage with heavily destroyed leaves) [26]. All ears were harvested per plot, field weight and grain weight were taken, and the percent grain moisture at harvest was determined using a moisture meter. Grain yield (Kg/ha) was computed using total field weight adjusted to 15% moisture content, and 80% shelling percentage [22].


$$\begin{array}{c} GrainYield\left(kg/ha\right)=\left(\frac{earweight\left(kg/plot\right)}{\text{plot area }\left({\text{m}}^{\text{2}}\right)}\times \text{0}.\text{80}\times \left(\frac{\left(\text{100 }-\text{ MC}\right)}{\text{8}\text{5}}\right)\times \text{10},\text{000}\right) \end{array}$$

### Statistical data analysis

Analysis of variance (ANOVA) was performed using the Multi-environment Trials Analysis (metan) package in R version 4.4.2 [27] to determine the differences among the hybrids, and trait means were separated with Fisher’s protected Least Significant Differences (LSD) test at 5% probability level. The ANOVA model treated all factors (genotype, environment, genotype × environment interaction, replicate within environment, and block within replicate and environment) as random effects, expressed as: $y_{ijkl}=\mu +g_{i}+e_{j}+(ge{)}_{ij}+r_{k(j)}+b_{l(kj)}+\varepsilon_{ijkl}$, where $y_{ijkl}$ is the observed value of a response variable for the ith genotype evaluated in the jth environment, replicate k within environment j, and block l within replicate k and environment j; μ is the overall mean; $g_{i}$ is the effect of the ith genotype; $e_{j}$ is the random effect of the jth environment; $(ge{)}_{ij}$ is the effect of the genotype × environment interaction; $r_{k(j)}$ is the effect of replicate k nested within environment j; $b_{l(kj)}$ is the effect of block l nested within replicate k and environment j; and $\varepsilon_{ijkl}$ is the residual error [28].

The genotypic variance ($\overset{\text{2}}{\underset{g}{\sigma }}$), genotype × environment interaction variance ($\overset{\text{2}}{\underset{ge}{\sigma }}$), and residual error variance ($\overset{\text{2}}{\underset{e}{\sigma }}$) were estimated using META-R v6.0 [29]. The Principal Component Analysis (PCA) was performed using the “prcomp” function in R version 4.4.2 to reduce the dimensionality and identify the most significant traits contributing to the variability of the hybrids. Those PCs with eigenvalues >1 were selected [30]. Pearson’s correlation analysis was computed to determine the strength and direction of associations among the measured traits using the ‘metan’ package in R version 4.4.2 [27]. Genotype by yield × trait (GYT) analysis and biplot were visualized in R 4.4.2 to evaluate multiple traits in relation to grain yield simultaneously and to rank hybrids for superiority based on multi-trait performance [31,32].

## Results

### Analysis of variance

Results of analysis of variance shown in Table 2 indicated significant hybrid effects (p < 0.05) for grain yield, plant aspect, ear aspect, southern corn leaf blight, *curvularia* leaf spot, and foliar FAW damage at 8 and 12 weeks after planting (WAP). Significant hybrid × environment interaction effects (p < 0.05) were observed for plant aspect, ear aspect, southern corn leaf blight, and foliar FAW damage at 8 WAP. Environmental effects were significant (p < 0.05) for all traits, except grain yield and ear aspect. The coefficients of variation (CV) were below 20% for plant aspect, ear aspect, ears per plant, and *curvularia* leaf spot. In contrast, grain yield, plant height, ear height, husk cover, ear rot, maize streak virus, southern corn leaf rust, southern corn leaf blight, and foliar FAW damage at 4, 8 and 12 WAP had CV values above 20%. The coefficients of determination (R²) were generally moderate to high (0.54–0.91) for most of the traits

**Table 2:** Mean squares of grain yield, other agronomic traits, foliar diseases, and foliar FAW damage of FAW-resistant maize hybrids and checks evaluated across two environments in the derived savanna agro-ecology of Nigeria during the minor season of 2024.

Source of Variation	df	GY (Kg/ha)	PH (cm)	EH (cm)	HC (1–5)	PASP (1–5)	EPP (count)	EASP (1–5)	EROT (1–5)
**Hybrid**	31	944986.05^***^	4872.88	1457.97	0.45	0.56^*^	0.03	0.45^*^	0.47
**Hybrid × Environment**	31	458714.73	5795.06	1433.73	0.58	0.50^*^	0.02	0.48^*^	0.43
**Environment**	1	157331.98	73310.09^***^	19528.27^***^	23.31^***^	6.75^***^	3.57^***^	0.29	2.88^*^
**Replicate (Environment)**	4	8640746.71^***^	10090.06	1118.13	2.80^***^	3.10^***^	0.14^***^	1.90^***^	1.30^*^
**Block (Environment × Replicate)**	42	749233.82^**^	5804.50	1772.87	0.80	0.6^**^	0.02	0.28	0.51
**Error**	82	361892.90	5417.27	1545.23	0.54	0.31	0.02	0.28	0.44
**R** ^**2**^		0.79	0.61	0.60	0.69	0.75	0.82	0.68	0.61
**CV (%)**		27.52	46.4	52.02	24.12	15.32	14.84	16.57	22.02
**Source of Variation**	**df**	**MSV** **(1–5)**	**SCLR** **(1–5)**	**SCLB** **(1–5)**	**CLS** **(1–5)**	**FFAWD4** **(1–9)**	**FFAWD8** **(1–9)**	**FFAWD12** **(1–9)**	
**Hybrid**	31	0.42	0.36	0.93^**^	0.63^*^	1.28	1.57^*^	1.42^*^	
**Hybrid × Environment**	31	0.46	0.38	1.03^**^	0.48	1.39	1.59^*^	1.12	
**Environment**	1	2.88^*^	1.88^*^	35.88^***^	232.98^***^	8.33^**^	180.19^***^	292.55^***^	
**Replicate (Environment)**	4	0.08	0.77^*^	2.18^**^	0.10	8.60^***^	11.13^***^	3.29^**^	
**Block (Environment × Replicate)**	42	0.48	0.33	0.75^*^	0.27	1.13	1.92^**^	1.62^**^	
**Error**	82	0.52	0.29	0.45	0.33	1.01	0.93	0.86	
**R** ^**2**^		0.54	0.64	0.79	0.91	0.68	0.84	0.87	
**CV (%)**		44.49	30.2	29.18	17.29	28.49	32.85	29.09	

*, **, *** = Significance at 0.05, 0.01, and 0.001 probability levels respectively; GY = grain yield; PH = plant height; EH = ear height; HC = husk cover; PASP = plant aspect; EPP = ears per plant; EASP = ear aspect; EROT = ear rot; MSV = maize streak virus; SCLR = southern corn leaf rust; SCLB = southern corn leaf blight; CLS = *curvularia* leaf spot; FFAWD4, FFAWD8, FFAWD12 = foliar FAW damage ratings at 4, 8, and 12 WAP; df = degrees of freedom; R^2^  = coefficient of determination; CV (%) = coefficient of variation.

### Mean performance

The mean performance of the evaluated hybrids is shown in Table 3. Grain yield ranged between 1,452.76 Kg/ha in FAWSYN-2 and 3,127.84 Kg/ha in FAWSYN-1/(TZLComp. 1 C6-W-39-1-1)-B-B, with a trial mean of 2,185.98 Kg/ha. Relative to the best commercial check, Oba Super 11 with 2,979.36 Kg/ha, the top-performing hybrid showed a 5% yield advantage. Plant height ranged from 139.77 cm in FAWSYN-2/TZISTR2129–2 to 309.83 cm in FAWSYN-2/IITATZI2305. Ear height ranged from 64.88 cm in FAWSYN-1 to 156.04 cm in FAWSYN-1/TZISTR1305. Husk cover scores (1–5) ranged from 2.50 in FAWSYN-1/TZISTR2042 to 3.58 in FAWSYN-1/(TZLComp. 1 C6-W-39-1-1)-B-B and FAWSYN-1/TZISTR1305. Plant aspect scores (1–5) ranged from 3.08 in FAWSYN-1/TZISTR1121 to 4.33 in FAWSYN-1/TZISTR1305. Ears per plant ranged from 0.73 in FAWSYN-1/TZISTR1129 to 1.05 in FAWSYN-3/IITATZI2300. Ear aspect scores (1–5) ranged from 2.75 in FAWSYN-1/IITATZI2305 to 4.00 in SC301. Ear rot scores (1–5) ranged from 2.58 in FAWSYN-2/TZISTR1129, FAWSYN-3/TZISTR1878, and FAWSYN-1/TZISTR1305 to 3.42 in FAWSYN-1/TZISTR2024, FAWSYN-2/TZISTR2024, SC301, and Oba Super 9. Maize streak virus scores (1–5) ranged from 1.17 in FAWSYN-3/TZISTR1869 and SAMMAZ 51 to 2.17 in FAWSYN-1/TZISTR1121. Southern corn leaf rust scores (1–5) ranged from 1.33 in Oba Super 11 to 2.25 in FAWSYN-1/IITATZI2305 and FAWSYN-2/IITATZI2300. Southern corn leaf blight scores (1–5) ranged from 1.33 in FAWSYN-1/TZISTR2024 to 3.17 in FAWSYN-1/IITATZI2305. *Curvularia* leaf spot scores (1–5) ranged from 2.08 in Oba Super 11 to 4.50 in FAWSYN-3/TZISTR1878 and Oba Super II. Foliar FAW damage ratings (1–9) at 4 WAP ranged from 2.00 in FAWSYN-1/(TZLComp. 1 C6-W-39-1-1)-B-B, FAWSYN-1/TZISTR2042, Oba Super 11, and Oba Super 9 to 4.00 in FAWSYN-3/TZISTR1872 and SC301. At 8 WAP, it ranged from 2.67 in FAWSYN-1, FAWSYN-3/IITATZI2300, FAWSYN-1/TZISTR1305, FAWSYN-3/TZISTR1872, and Oba Super 7 to 4.00 in FAWSYN-1/TZISTR1878, SC301, and Oba Super II, while at 12 WAP, it ranged from 2.33 in FAWSYN-1/TZISTR2042 to 4.17 in FAWSYN-2.

**Table 3:** Mean performance of FAW-resistant maize hybrids and checks for grain yield, other agronomic traits, foliar diseases, and foliar FAW damage across environments in 2024.

HYBRID	GY (Kg/ha)	PH (cm)	EH (cm)	HC (1–5)	PASP (1–5)	EPP (count)	EASP (1–5)	EROT (1–5)	MSV (1–5)	SCLR (1–5)	SCLB (1–5)	CLS (1–5)	FFAWD4 (1–9)	FFAWD8 (1–9)	FFAWD12 (1–9)
**FAWSYN-1/(TZLComp. 1 C6-W-39-1-1)-B-B**	3127.84	165.11	76.98	3.58	3.17	0.93	2.92	3.00	1.25	2.00	2.17	3.58	3.00	2.00	3.33
**FAWSYN-2/(TZLComp. 1 C6-W-39-1-1)-B-B**	2842.10	170.23	87.04	2.83	3.25	0.95	3.08	3.08	1.25	2.17	2.33	3.50	3.33	3.33	3.17
**FAWSYN-1/IITATZI2305**	2717.11	155.94	68.97	3.08	3.33	0.97	2.75	2.75	1.92	2.25	3.17	3.25	3.67	2.50	2.50
**FAWSYN-3/IITATZI2305**	2632.29	162.35	79.39	2.58	3.58	0.96	3.08	3.08	1.42	1.50	2.25	3.58	3.33	2.67	3.33
**FAWSYN-1/TZISTR1121**	2577.07	148.85	70.35	3.00	3.08	0.93	3.08	2.67	2.17	1.75	2.25	3.50	3.50	2.50	2.67
**FAWSYN-1/TZISTR2042**	2420.24	155.99	74.32	2.50	3.17	1.04	3.50	3.17	1.58	2.00	2.17	3.75	2.67	2.50	2.33
**FAWSYN-3/IITATZI2300**	2409.35	156.60	75.92	2.75	3.33	1.05	3.08	3.33	1.92	1.75	2.25	3.50	3.17	2.33	2.50
**FAWSYN-2/IITATZI2300**	2343.79	156.60	70.85	3.17	3.58	0.93	3.17	3.17	1.67	2.25	2.33	3.33	3.17	2.67	2.83
**FAWSYN-1/IITATZI2300**	2323.15	161.22	75.22	3.00	3.25	0.93	2.92	3.08	1.83	2.00	2.17	3.58	3.17	2.67	2.83
**FAWSYN-3/TZISTR1869**	2200.63	155.22	79.04	3.00	3.83	0.96	3.25	3.08	1.17	1.75	2.33	3.08	3.67	3.17	3.17
**FAWSYN-1/TZISTR2024**	2197.74	151.08	71.51	3.00	3.42	0.89	3.58	3.42	1.75	1.50	1.33	3.33	2.83	3.33	2.50
**FAWSYN-1/TZISTR1878**	2193.70	157.81	76.46	2.83	3.67	0.90	3.33	3.17	1.92	1.50	2.67	3.33	4.33	3.50	4.00
**FAWSYN-2/TZISTR1129**	2172.43	152.17	66.56	3.00	3.58	0.90	2.92	2.58	1.42	1.58	2.42	3.33	3.83	3.17	3.67
**FAWSYN-2/IITATZI2305**	2152.97	309.83	65.74	2.92	3.67	0.88	2.83	2.83	2.08	1.58	2.92	3.50	3.50	2.83	3.83
**FAWSYN-2/TZISTR1878**	2117.46	156.39	68.19	3.00	3.17	0.94	3.50	3.25	1.83	1.58	2.58	3.17	3.50	2.83	3.00
**FAWSYN-3**	2048.60	148.01	71.67	3.33	3.75	0.92	3.67	2.92	1.67	1.42	1.75	3.50	3.83	3.17	3.00
**FAWSYN-2/TZISTR2129–2**	2025.88	139.77	70.56	3.50	3.67	0.89	3.00	3.00	1.67	1.83	2.75	3.25	3.17	2.50	2.67
**FAWSYN-1/TZISTR1869**	1968.10	155.58	70.06	3.00	3.67	0.78	3.33	2.67	1.75	1.67	2.17	3.33	3.50	2.83	3.00
**FAWSYN-2/TZISTR2024**	1929.36	155.64	72.85	3.25	3.83	0.88	3.25	3.42	1.75	1.75	1.75	3.17	3.50	2.83	3.00
**FAWSYN-3/TZISTR1878**	1924.39	157.86	77.23	3.33	3.75	0.83	3.25	2.58	1.75	1.75	2.92	3.33	4.50	3.67	2.67
**FAWSYN-1**	1767.63	142.60	64.88	3.25	4.00	0.83	2.92	2.83	1.58	2.17	2.00	3.42	3.50	2.67	3.50
**FAWSYN-2/TZISTR1305**	1740.76	150.08	70.51	2.83	4.00	0.87	2.83	2.75	1.83	1.92	2.58	3.42	3.17	2.67	3.17
**FAWSYN-1/TZISTR1129**	1739.74	158.57	77.94	3.42	3.75	0.73	2.92	2.75	1.58	1.50	2.00	2.83	3.50	3.17	3.67
**FAWSYN-1/TZISTR1305**	1640.63	146.94	156.04	3.58	4.33	0.83	3.00	2.58	1.58	1.75	2.58	3.33	3.33	2.50	3.00
**FAWSYN-3/TZISTR1872**	1639.52	147.85	65.24	3.25	4.00	0.82	3.08	2.75	1.75	1.75	2.08	3.50	2.83	4.00	3.50
**FAWSYN-2**	1452.76	139.91	66.50	2.75	3.58	0.94	3.42	3.33	1.33	1.83	2.00	3.08	3.33	3.17	4.17
**Checks**															
**SAMMAZ 51**	2236.18	145.62	73.84	2.92	3.75	0.94	3.33	2.67	1.17	1.92	2.58	3.67	4.00	3.67	3.17
**Oba Super 11**	2979.36	154.74	77.28	2.83	3.25	0.93	3.25	3.33	1.83	1.33	1.92	2.08	3.67	2.00	3.00
**SC301**	1902.07	165.52	72.08	3.07	3.58	0.92	4.00	3.42	1.50	2.00	2.67	3.50	4.33	4.00	3.50
**Oba Super 7**	2161.48	152.77	77.84	2.92	3.83	0.99	3.17	3.08	1.33	1.58	2.50	3.42	3.83	2.67	4.00
**Oba Super 9**	2438.78	154.32	80.90	2.92	3.58	0.93	3.17	3.42	1.42	1.92	2.50	3.33	3.83	3.00	3.83
**Oba Super II**	1928.39	145.23	66.31	3.42	4.08	0.97	3.33	3.25	1.25	1.58	1.75	2.58	4.50	3.50	3.33
**Statistics**															
**Mean**	2185.98	158.64	75.57	3.06	3.61	0.91	3.18	3.01	1.62	1.78	2.31	3.32	3.53	2.94	3.18
**LSD(0.05)**	690.93	84.53	45.15	0.85	0.64	0.16	0.61	0.76	0.83	0.62	0.77	0.66	1.16	1.11	1.06

GY = grain yield; PH = plant height; EH = ear height; HC = husk cover; PASP = plant aspect; EPP = ears per plant; EASP = ear aspect; EROT = ear rot; MSV = maize streak virus; SCLR = southern corn leaf rust; SCLB = southern corn leaf blight; CLS = *curvularia* leaf spot; FFAWD4, FFAWD8, FFAWD12 = foliar FAW damage ratings at 4, 8, and 12 WAP; LSD(0.05) = Least Significant difference at 5% probability level.

### Variance components

Variance component analysis (Table 4) showed that the phenotypic variance exceeded the genotypic variance for all traits, with grain yield having the highest genotypic variance (44145.37) and phenotypic variance (387199.03). Grain yield had a Phenotypic Coefficient of Variation (PCV) of 14.56% and a Genotypic Coefficient of Variation (GCV) of 9.61%. Other traits, such as plant height, ear height, husk cover, plant and ear aspects, and ears per plant, often showed low genotypic variance, often close to zero. *Curvularia* leaf spot and foliar fall armyworm damage at 12 WAP resulted in GCV values of 4.74% and 4.21%, respectively. The PCV values were higher than the GCV.

**Table 4:** Variance components for grain yield, agronomic traits, foliar disease scores, and foliar FAW damage across environments in 2024.

Trait	σ²g	σ²ge	σ²e	σ²p	GCV(%)	PCV(%)
**Grain yield (Kg/ha)**	44145.37	0.00	343053.66	387199.03	9.61	14.56
**Plant height (cm)**	0.00	0.00	5383.13	5383.13	0.00	46.25
**Ear height (cm)**	0.00	0.00	1554.13	1554.13	0.00	52.17
**Husk cover (1–5)**	0.00	0.00	0.55	0.55	0.00	24.20
**Plant aspect (1–5)**	0.00	0.03	0.31	0.34	0.00	15.39
**Ears per plant (count)**	0.00	0.00	0.02	0.02	1.91	6.77
**Ear aspect (1–5)**	0.00	0.05	0.26	0.31	0.00	15.86
**Ear rot (1–5)**	0.00	0.00	0.40	0.40	1.83	8.76
**Maize streak virus (1–5)**	0.00	0.00	0.47	0.47	0.00	42.10
**Southern corn leaf rust (1–5)**	0.00	0.02	0.30	0.32	0.00	30.92
**Southern corn leaf blight (1–5)**	0.00	0.16	0.47	0.63	0.00	29.73
***Curvularia* leaf spot (1–5)**	0.02	0.06	0.30	0.39	4.74	9.76
**Foliar FAW damage at 4 WAP (1–9)**	0.01	0.05	0.94	1.00	2.07	12.22
**Foliar FAW damage at 8 WAP (1–9)**	0.00	0.03	0.83	0.87	0.00	13.42
**Foliar FAW damage at 12 WAP (1–9)**	0.02	0.00	0.83	0.85	4.21	12.44

σ²g = genotypic variance; σ²ge = genotype × environment interaction variance; σ²p = phenotypic variance; σ²e = residual error variance; GCV (%) = Genotypic Coefficient of Variation; PCV (%) = Phenotypic Coefficient of Variation.

### Principal component analysis

Principal Component Analysis (PCA) showed the contribution of grain yield, agronomic traits, foliar diseases, and foliar FAW damage to variation among the maize hybrids and checks evaluated across environments (Table 5). The first five principal components (PC) cumulatively accounted for 69% of the total variation. The PC1 explained 22% of the variation with an eigenvalue of 3.28 and was characterised by high absolute loadings for plant aspect (PASP; 0.47), grain yield (GY; −0.43), ears per plant (EPP; −0.39), and husk cover (HC; 0.30). The PC2 explained 17% of the variation with an eigenvalue of 2.49 and was characterized by ear aspect (EASP; −0.50), ear rot (EROT; −0.46), and southern corn leaf blight (SCLB; 0.32). The PC3 accounted for 12% of the variation with an eigenvalue of 1.75 and was characterized by SCLB (−0.49), HC (0.31), *Curvularia* leaf spot (CLS; −0.33), and foliar FAW damage at 4, 8, and 12 WAP (FFAWD4, FFAWD8, and FFAWD12; −0.31, −0.32, and −0.32, respectively). The PC4 explained 11% of the variation with an eigenvalue of 1.59 and was characterized by southern corn leaf rust (SCLR; −0.52), maize streak virus (MSV; 0.49), CLS (−0.37), and plant height (PH; 0.43). The PC5 explained 9% of the total variation with an eigenvalue of 1.28 and was characterized by CLS (0.40), MSV (0.39), GY (−0.36), ear height (EH; −0.34), and FFAWD4 (−0.33).

**Table 5:** Principal component analysis of the contributions of grain yield, other agronomic traits, foliar diseases, and foliar FAW damage to total variation among the maize hybrids and checks evaluated across environments.

Traits	Eigenvectors				
	PC1	PC2	PC3	PC4	PC5
**Grain yield (kg ha**^**−1**^)	−0.43	0.03	−0.02	0.04	−0.36
**Plant height (cm)**	−0.05	0.17	−0.38	0.43	0.00
**Ear height (cm)**	0.12	0.20	0.22	−0.22	−0.34
**Husk cover (1–5)**	0.30	0.19	0.31	−0.09	−0.10
**Plant aspect (1–5)**	0.47	0.06	0.05	−0.11	−0.06
**Ears per plant (count)**	−0.39	−0.22	−0.15	−0.19	−0.22
**Ear aspect (1–5)**	0.03	−0.50	−0.04	−0.11	0.25
**Ear rot (1–5)**	−0.21	−0.46	0.07	0.01	−0.06
**Maize streak virus (1–5)**	−0.11	0.23	0.05	0.49	0.39
**Southern corn leaf rust (1–5)**	−0.15	0.23	−0.16	−0.52	0.06
**Southern corn leaf blight (1–5)**	0.00	0.32	−0.49	−0.03	−0.22
***Curvularia* leaf spot (1–5)**	−0.11	0.22	−0.33	−0.37	0.40
**Foliar FAW damage at 4 WAP (1–9)**	0.26	−0.20	−0.31	0.10	−0.33
**Foliar FAW damage at 8 WAP (1–9)**	0.32	−0.26	−0.32	−0.14	0.29
**Foliar FAW damage at 12 WAP (1–9)**	0.27	−0.14	−0.32	0.13	−0.27
**Eigenvalue**	3.28	2.49	1.75	1.59	1.28
**Proportion (%)**	22	17	12	11	9
**Cumulative Proportion (%)**	22	38	50	61	69

PC = principal component; bolded values (|loading| ≥ 0.30) contributed more to the variation.

**Fig 1:**
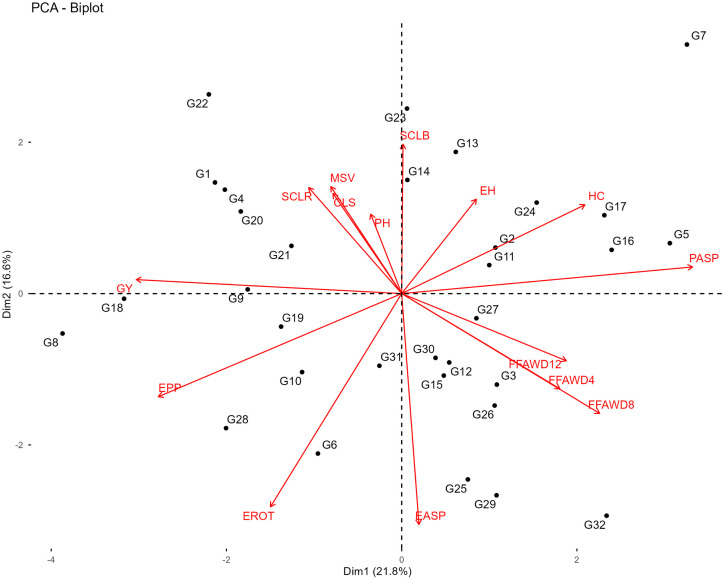
The PCA biplot showing the relationship between PC1 and PC2 for contributions of grain yield, foliar diseases, and other agronomic traits to total variation among the maize hybrids and checks evaluated across environments. GY = grain yield; PH = plant height; EH = ear height; HC = husk cover; PASP = plant aspect; EPP = ears per plant; EASP = ear aspect; EROT = ear rot; MSV = maize streak virus; SCLR = southern corn leaf rust; SCLB = southern corn leaf blight; CLS = *curvularia* leaf spot; FFAWD4, FFAWD8, FFAWD12 = foliar FAW damage ratings at 4, 8, and 12 WAP; G1-G32 = maize hybrids as presented in Table 1.

The PCA biplot is illustrated in Fig 1. Along PC1, PASP, HC, EH, FFAWD4, FFAWD8, and FFAWD12 were positioned on the positive side of the axis, whereas GY, EPP, PH, MSV, EASP, EROT, SCLR, SCLB, and CLS were positioned on the negative side. Along PC2, GY, HC, MSV, SCLR, SCLB, and CLS were positioned on the positive side, whereas EPP, EASP, EROT, FFAWD4, FFAWD8, and FFAWD12 were positioned on the negative side. Genotypes G5, G7, G16, G17, and G24 were positioned on the positive side of PC1, whereas G1, G8, G9, G18, and G22 were located on the negative side. Genotypes G13, G14, and G23 were positioned in the positive PC2 quadrant, while G3, G25, G26, and G29 were located in the negative PC2 quadrant.

### Correlation analysis

**Fig 2:**
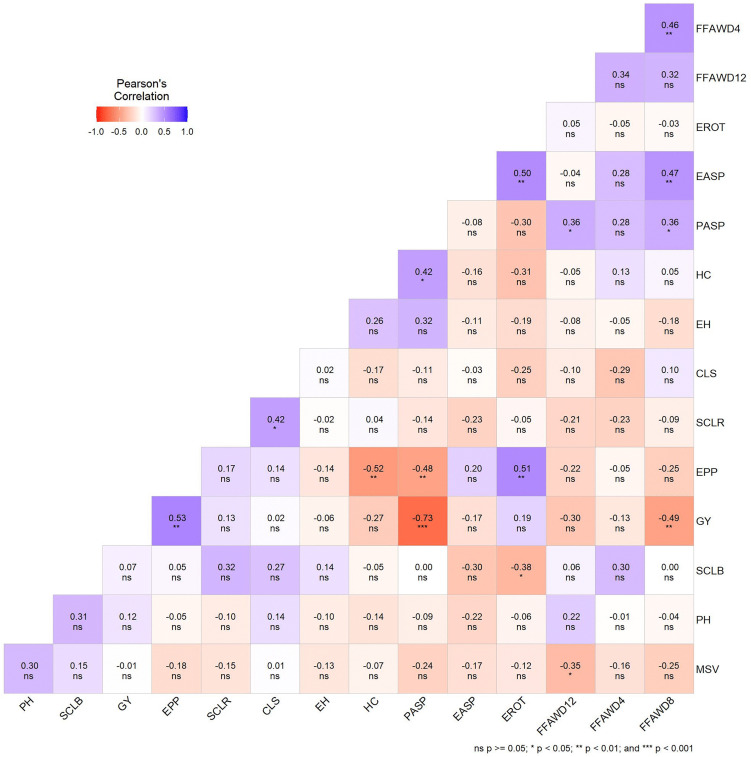
Correlogram showing the relationship between traits of FAW-resistant maize hybrids and checks evaluated across environments. The cell value denotes correlation coefficient (r) values; ^*,**,***^ = Significance at 0.05, 0.01, and 0.001 probability levels respectively; ns = non-significance; GY = grain yield, PH = plant height, EH = ear height. HC = husk cover, PASP = plant aspect, EPP = ears per plant, EASP = ear aspect, EROT = ear rot, MSV = maize streak virus, SCLR = southern corn leaf rust, SCLB = southern corn leaf blight, CLS = *curvularia* leaf spot, FFAWD4 = foliar FAW damage at 4 WAP, FFAWD8 = foliar FAW damage at 8 WAP, FFAWD12 = foliar FAW damage at 12 WAP.

Several significant positive and negative Pearson’s correlation coefficients were identified among the traits evaluated across environments (Fig 2). Grain yield had a strong negative correlation coefficient with plant aspect (r = −0.73, p < 0.001), a strong positive correlation coefficient with ears per plant (r = 0.53, p < 0.01), and a moderate negative correlation coefficient with foliar fall armyworm damage at 8 WAP (r = −0.49, p < 0.01). Husk cover had a moderate negative correlation coefficient with ears per plant (r = −0.52, p < 0.01) but a moderate positive correlation coefficient with plant aspect (r = 0.42, p < 0.05). Ears per plant had a moderate positive correlation coefficient with ear rot (r = 0.51, p < 0.01) and a moderate negative correlation coefficient with plant aspect (r = −0.48, p < 0.01).

Ear aspect had a moderate positive correlation coefficient with ear rot (r = 0.50, p < 0.01) and a moderate positive correlation coefficient with foliar fall armyworm damage at 8 WAP (r = 0.47, p < 0.01). Foliar fall armyworm damage at 4 WAP had a moderate positive correlation coefficient with damage at 8 WAP (r = 0.46, p < 0.01). Plant aspect had moderate positive correlation coefficients with foliar fall armyworm damage at 8 WAP (r = 0.36, p < 0.05) and 12 WAP (r = 0.36, p < 0.05). Maize streak virus had a moderate negative correlation coefficient with foliar fall armyworm damage at 12 WAP (r = −0.35, p < 0.05). In addition, southern corn leaf rust had a moderate positive correlation coefficient with CLS (r = 0.42, p < 0.05), while ear rot had a moderate negative correlation coefficient with southern corn leaf blight (r = −0.38, p < 0.05).

### Genotype by yield × trait analysis and superiority index

**Fig 3:**
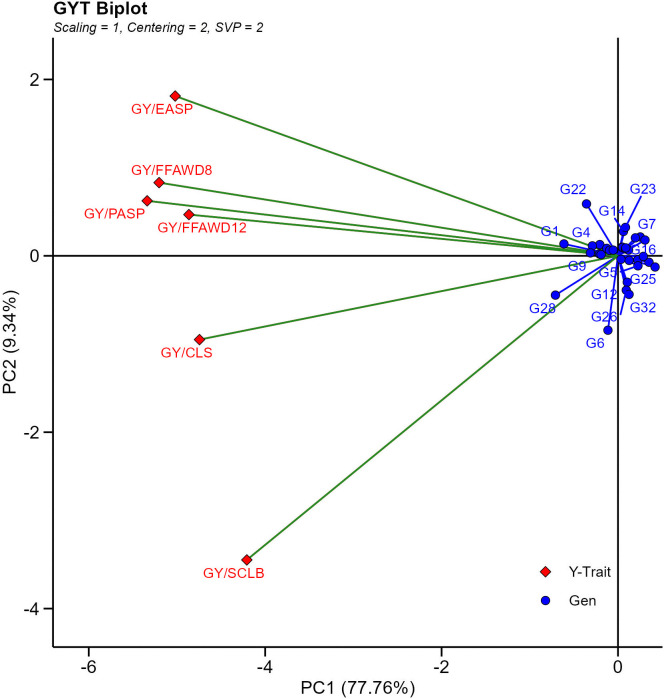
GYT biplot showing the relationships among the yield-trait combination of the 32 FAW-resistant maize hybrids and checks evaluated across environments in 2024. GY = grain yield, PASP = plant aspect, EASP = ear aspect, SCLB = southern corn leaf blight, CLS = *curvularia* leaf spot, FFAWD8, FFAWD12 = foliar FAW damage at 8 and 12 WAP, G1-G32 = maize hybrids as presented in Table 1.

**Fig 4:**
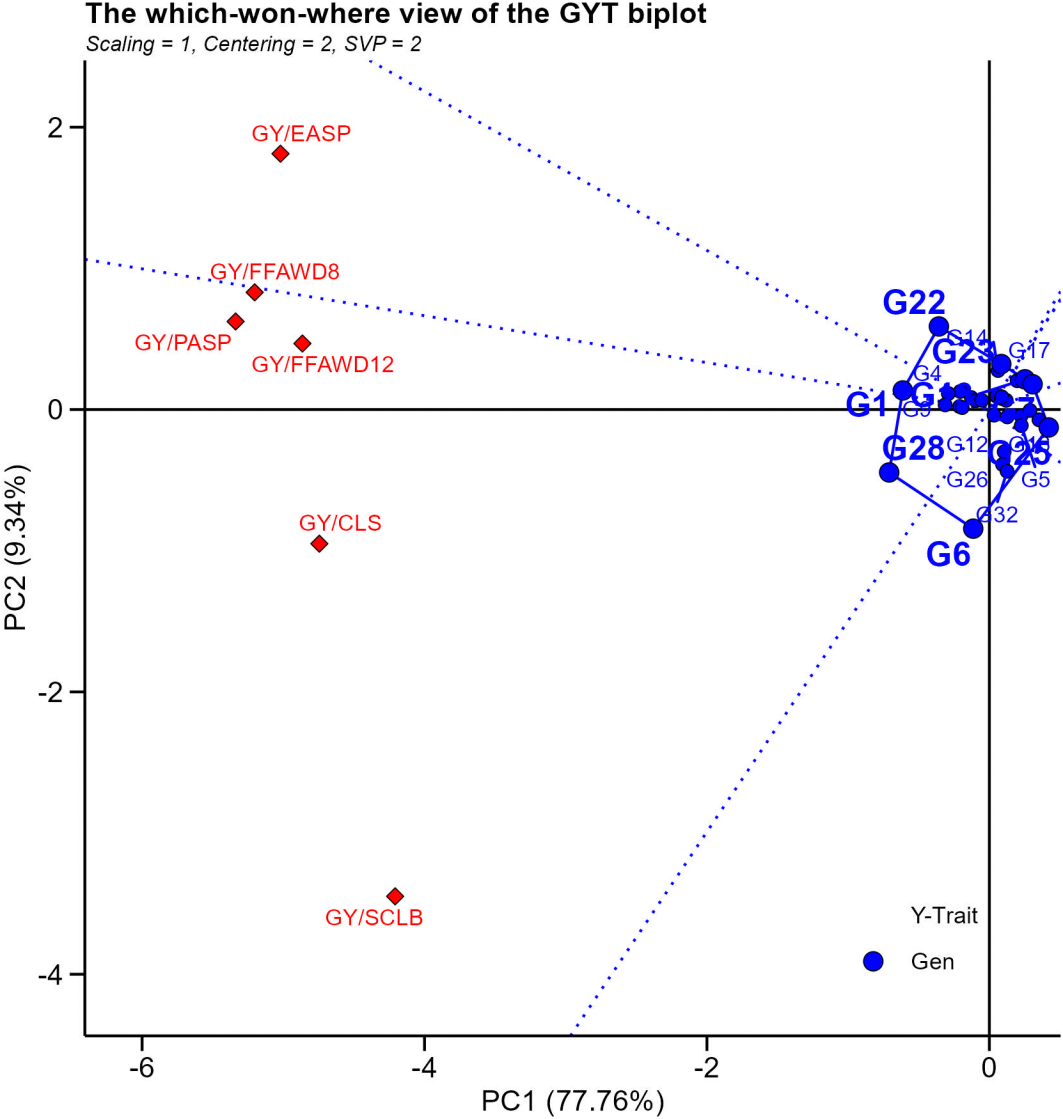
Which-won-where view of the GYT biplot for the 32 FAW-resistant maize hybrids and checks evaluated across environments in 2024. GY = grain yield, PASP = plant aspect, EASP = ear aspect, SCLB = southern corn leaf blight, CLS = *curvularia* leaf spot, FFAWD8, FFAWD12 = foliar FAW damage at 8 and 12 WAP, G1-G32 = maize hybrids as presented in Table 1.

**Fig 5:**
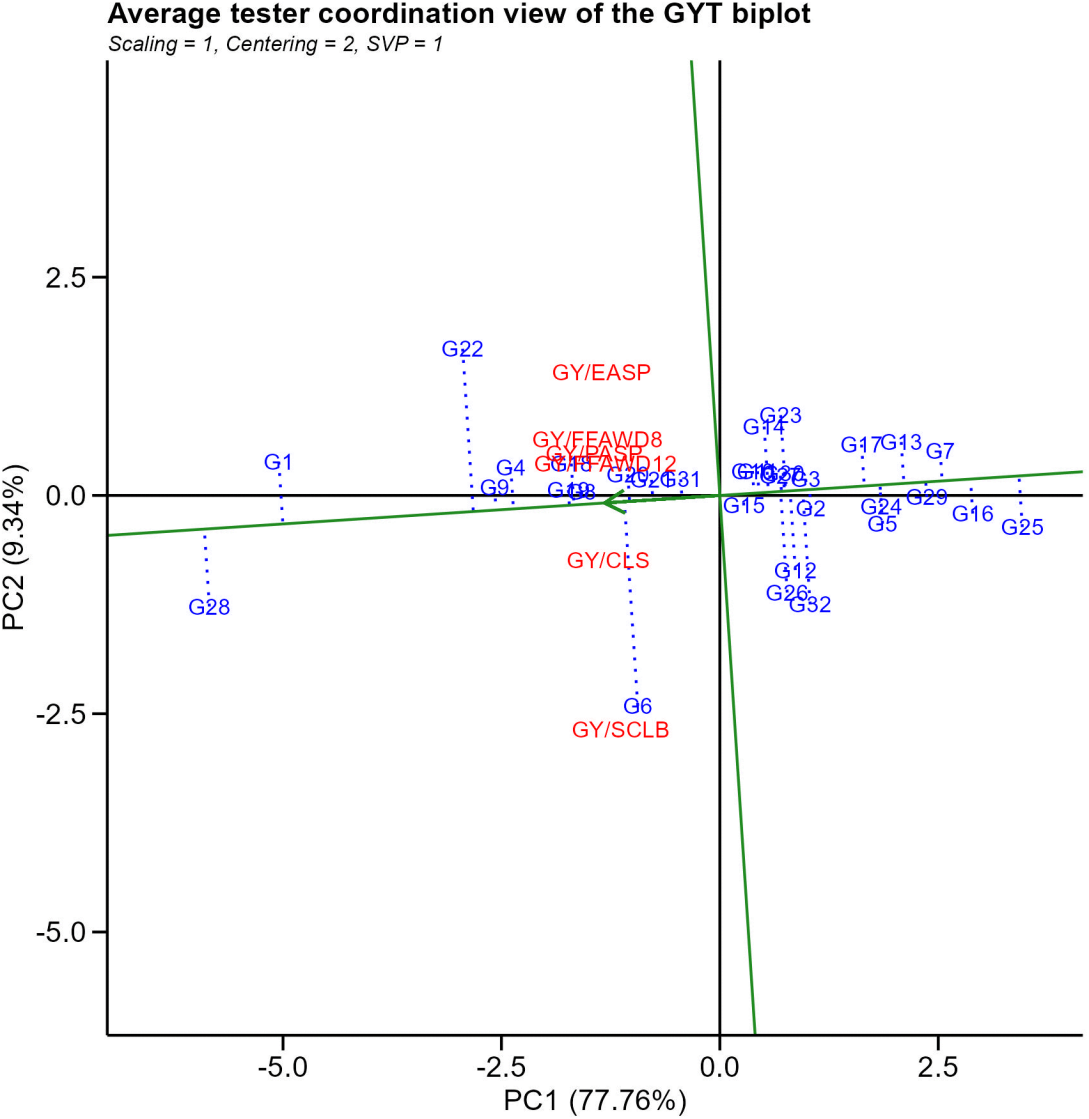
The Average Tester Coordination view of the genotype by yield× trait (GYT) biplot for the 32 FAW-resistant maize hybrids and checks evaluated across environments in 2024. GY = grain yield, PASP = plant aspect, EASP = ear aspect, SCLB = southern corn leaf blight, CLS = *curvularia* leaf spot, FFAWD8, FFAWD12 = foliar FAW damage at 8 and 12 WAP, G1-G32 = maize hybrids as presented in Table 1.

The genotype by yield × trait (GYT) analysis combined grain yield with the traits that had a significant hybrid effect, including plant aspect, ear aspect, southern corn leaf blight, *curvularia* leaf spot, and foliar fall armyworm damage at 8 and 12 WAP to rank the hybrids. The standardized genotype by yield × trait combinations and the corresponding superiority index are presented in Table 6. The GYT biplot explained 87.10% of the total variation among the yield × trait combinations, with PC1 accounting for 77.76% and PC2 accounting for 9.34% (Fig 3). For clarity, entry numbers corresponding to each hybrid were used for ease of graphical presentation. Accordingly, G1 aligned with GY/PASP, GY/EASP, and GY/FFAWD8, G22 aligned with GY/FFAWD12, and G6 aligned with GY/SCLB, while the check Oba Super 11 (G28) aligned with GY/CLS. Based on the “which won where” analysis (Fig 4), the perpendicular lines divided the polygon into eight sectors, and the yield × trait combinations were captured in sectors with vertex entries G1 (GY/PASP, GY/EASP, and GY/FFAWD8), G22 (GY/FFAWD12), G6 (GY/SCLB), and the check G28 (GY/CLS). The Average Tester Coordination (ATC) view (Fig 5) identified the top five hybrids as FAWSYN-1/(TZLComp. 1 C6-W-39-1-1)-B-B (G1), FAWSYN-1/IITATZI2305 (G22), FAWSYN-2/(TZLComp. 1 C6-W-39-1-1)-B-B (G9), FAWSYN-1/TZISTR1121 (G4), and FAWSYN-3/IITATZI2305 (G19), whereas the bottom five were FAWSYN-2 (G25), FAWSYN-3/TZISTR1872 (G16), FAWSYN-1/TZISTR1305 (G7), FAWSYN-2/TZISTR1305 (G13), and FAWSYN-1/TZISTR1129 (G5). Among the checks, Oba Super 11 (G28) had the highest superiority index.

**Table 6:** Standardized genotype by yield × trait and superiority index of FAW-resistant maize hybrids evaluated across environments in 2024.

Hybrid	Pedigree	GY/PASP	GY/EASP	GY/SCLB	GY/CLS	GY/FFAWD8	GY/FFAWD12	Superiority Index
**G1**	FAWSYN-1/(TZLComp. 1 C6-W-39-1-1)-B-B	2.38	2.56	1.87	1.19	3.01	1.22	2.04
**G22**	FAWSYN-1/IITATZI2305	1.28	1.99	−0.47	0.98	1.18	2.01	1.16
**G9**	FAWSYN-2/(TZLComp. 1 C6-W-39-1-1)-B-B	1.66	1.55	0.97	0.84	0.28	1.01	1.05
**G4**	FAWSYN-1/TZISTR1121	1.41	0.97	0.68	0.40	0.96	1.37	0.96
**G19**	FAWSYN-3/IITATZI2305	0.76	1.09	0.78	0.39	0.80	0.43	0.71
**G18**	FAWSYN-3/IITATZI2300	0.68	0.60	0.38	0.12	0.97	1.36	0.68
**G8**	FAWSYN-1/TZISTR2042	0.95	−0.01	0.57	−0.14	0.72	1.75	0.64
**G6**	FAWSYN-1/TZISTR2024	0.17	−0.54	2.69	−0.05	−0.46	0.91	0.45
**G20**	FAWSYN-1/IITATZI2300	0.63	0.70	0.39	−0.12	0.35	0.59	0.42
**G21**	FAWSYN-2/IITATZI2300	0.24	0.32	0.12	0.20	0.38	0.63	0.32
**G15**	FAWSYN-3/TZISTR1869	−0.27	−0.10	−0.13	0.26	−0.32	−0.07	−0.11
**G10**	FAWSYN-2/TZISTR1878	0.33	−0.59	−0.62	0.00	−0.12	−0.01	−0.17
**G11**	FAWSYN-2/TZISTR1129	−0.07	0.35	−0.30	−0.10	−0.36	−0.62	−0.18
**G14**	FAWSYN-2/TZISTR2129–2	−0.41	−0.12	−0.95	−0.26	0.12	0.27	−0.23
**G26**	FAWSYN-3	−0.45	−0.90	0.78	−0.49	−0.51	−0.13	−0.28
**G23**	FAWSYN-2/IITATZI2305	−0.19	0.45	−0.95	−0.31	−0.08	−0.78	−0.31
**G12**	FAWSYN-2/TZISTR2024	−0.73	−0.67	0.51	−0.35	−0.38	−0.35	−0.33
**G3**	FAWSYN-1/TZISTR1878	−0.12	−0.23	−0.61	−0.06	−0.59	−0.85	−0.41
**G2**	FAWSYN-1/TZISTR1869	−0.51	−0.69	−0.27	−0.46	−0.33	−0.28	−0.42
**G17**	FAWSYN-3/TZISTR1878	−0.66	−0.68	−1.26	−0.53	−0.98	0.07	−0.67
**G24**	FAWSYN-1	−1.12	−0.58	−0.36	−0.88	−0.45	−1.08	−0.75
**G5**	FAWSYN-1/TZISTR1129	−0.98	−0.65	−0.42	−0.32	−0.88	−1.24	−0.75
**G13**	FAWSYN-2/TZISTR1305	−1.16	−0.53	−1.20	−0.93	−0.49	−0.84	−0.86
**G7**	FAWSYN-1/TZISTR1305	−1.53	−0.98	−1.36	−1.03	−0.47	−0.86	−1.04
**G16**	FAWSYN-3/TZISTR1872	−1.33	−1.09	−0.75	−1.17	−1.42	−1.27	−1.17
**G25**	FAWSYN-2	−1.36	−1.80	−0.99	−1.16	−1.23	−1.91	−1.41
**Checks**								
**G28**	Oba Super 11	1.93	1.51	2.31	4.45	2.72	1.51	2.41
**G31**	Oba Super 9	0.41	0.52	0.00	0.37	0.13	−0.38	0.18
**G27**	SAMMAZ 51	−0.13	−0.15	−0.44	−0.34	−0.65	−0.01	−0.29
**G30**	Oba Super 7	−0.34	−0.07	−0.44	−0.21	0.12	−0.89	−0.31
**G32**	Oba Super II	−0.93	−0.77	0.51	0.46	−0.88	−0.69	−0.38
**G29**	SC301	−0.55	−1.46	−1.04	−0.73	−1.16	−0.88	−0.97
**Mean**		0.00	0.00	0.00	0.00	0.00	0.00	
**Standard deviation**		1.00	1.00	1.00	1.00	1.00	1.00	

GY = grain yield, PASP = plant aspect, EASP = ear aspect, SCLB = southern corn leaf blight, CLS = *curvularia* leaf spot, FFAWD8, FFAWD12 = foliar FAW damage at 8 and 12 WAP.

## Discussion

Insufficient maize production in many regions is largely driven by FAW outbreaks, which could reduce maize yields and cause annual yield losses that jeopardize the livelihoods of smallholder farmers [11]. Cultivating resistant hybrids could reduce insecticide dependence, lower production costs, and contribute to sustainable food security in FAW-affected regions [22]. Thirty-two FAW-resistant maize hybrids and checks were evaluated across two environments exposed to natural FAW pressure to determine resistance levels and yield potential. Rainfall was highly irregular, and FAW pressure varied but remained sufficient at both experimental sites, allowing differentiation of hybrid responses.

The significant hybrid effect, along with hybrid × environment (G × E) and environment effects for several traits, indicated genetic variation whose expression depended on growing conditions. Similar patterns were reported in FAW-resistance studies in which genotype and G × E effects were significant for grain yield and FAW leaf and ear damage under artificial or natural infestation [33–35]. The significant environmental effects observed further indicated that environmental conditions strongly influenced both productivity and resistance expression [22,33,34]. The high R² values were consistent with reports of moderate-to-high heritability for FAW foliar damage and grain yield, which suggested that selection for resistance and agronomic performance could be effective when trials were appropriately replicated across environment [22,36]. The coefficients of variation above 20% for grain yield and foliar FAW damage were consistent with FAW screening trials and likely reflected heterogeneous pest pressure and complex FAW damage–yield relationships [33–35].

Grain yield levels were lower than those reported in FAW-tolerant hybrid trials conducted under on-station, artificial infestation, or well-managed conditions, where tolerant hybrids frequently produced 4.6–7.1 t/ha, and some entries exceeded 7 t/ha under FAW pressure [22,36,37]. However, comparable or lower yields were reported under natural infestation and smallholder-type stress conditions, such as in Mozambique, where the mean yield was approximately 0.8 t/ha, and the best entry reached about 1.3 t/ha [38]. This contrast suggested that yield performance was strongly influenced by the intensity of environmental stress, particularly irregular rainfall, agronomic management, and pest pressure [22,36]. Foliar FAW damage levels were consistent with ranges reported in hybrid and open-pollinated maize hybrids evaluated under artificial infestation, where damage scores commonly ranged from moderate to moderately high depending on genotype and infestation timing [33,35,37]. Similar studies reported that hybrids with lower foliar FAW damage tended to maintain higher grain yield, reflecting the negative relationship between foliar FAW damage and productivity [33,39]. Ear aspect, husk cover, and ear rot responses aligned with reports describing FAW-tolerant hybrids as possessing acceptable ear quality, improved husk protection, and reduced secondary ear damage, thereby helping maintain yield under infestation [22,37,39]. Comparable performance of commercial checks relative to trial entries was also reported in FAW screening studies, in which the checks generally performed within the same performance range as many trial entries, although they were often surpassed by improved FAW-resistant hybrids [22,34,35].

The negligible or zero genotypic variance for most agronomic and disease traits and for several foliar FAW damage scores indicated that environmental factors played a dominant role in trait expression, thereby masking the genotypic expression of these traits [40]. This could be due to spatially heterogeneous factors or environmental variability, such as soil fertility or moisture, which may also have been absorbed into the residual term. In addition, the limited number of test environments, comprising two similar locations evaluated within a single year, may not have provided sufficient environmental diversity for genotypes to fully express their genetic potential, thereby contributing to the low estimated genotypic variance. However, some FAW studies that applied mixed-model approaches, including spatial or related adjustments, often reported reduced residual variance and recovered larger genotypic variance components [29,41]. Genotype composition may also have contributed; closely related elite maize hybrids could have expressed genuinely smaller among-genotype variance for several traits than more diverse sets that included broad germplasm sources, landraces, or open-pollinated varieties [33,34].

The first two principal components were primarily associated with agronomic and ear-quality traits, whereas foliar FAW damage scores contributed more strongly to later components. This differed from other FAW studies, in which early principal components were dominated by foliar FAW-damage variables and their association with grain yield [36,42]. This could be due to the inclusion of broader agronomic traits, consistent with studies that predominantly focus on plant architecture and yield-related variables, such as plant height, ear traits, and grain yield [43–45]. This pattern suggested that variation among maize hybrids was more strongly explained by plant morphology, yield components, and disease-related traits than by foliar FAW damage scores alone.

The Pearson’s correlation pattern indicated that grain yield was closely associated with plant architecture and key yield components, consistent with FAW-infested multi-environment trials in which ears per plant, plant height, ear height, and ear-related traits were major contributors to yield variation under stress conditions [34,36,38]. Negative associations between foliar FAW damage and grain yield were also consistent with findings from Mozambique, Nepal, and other FAW-affected environments, where foliar and ear damage reduced grain yield and ear development. However, the magnitude of these relationships depended on genotype and infestation intensity [46,47]. Similar relationships were reported under both controlled infestation and field conditions, indicating that foliar FAW damage reduced productivity primarily through its effects on plant vigor and ear development [22,48,49]. Consistent associations among foliar FAW damage scores across the growth stages and with agronomic traits were also reported, reflecting the tendency for susceptible maize hybrids to exhibit sustained injury and reduced agronomic performance, whereas tolerant hybrids maintained more favorable growth and yield characteristics under infestation [34,36].

Grain yield is a genetically complex trait influenced by multiple agronomic and environmental factors; therefore, direct selection for yield alone is often inefficient, particularly under variable stress conditions [50]. In contrast, phenology, growth, and resistance-related traits often exhibited higher heritability and lower genotype × environment sensitivity, making them more reliable targets for indirect selection [51]. Multi-trait indices such as GYT therefore provided a more robust basis for advancement because they ranked genotypes using yield–trait combinations that jointly captured productivity, stress tolerance, and overall plant type [31,52]. The high ranking of the commercial check, OBA Super 11, on the GYT superiority index indicated a favorable aggregate profile of grain yield, phenotypic appeal, tolerance to foliar diseases, and tolerance to FAW foliar damage, consistent with reports in which commercial checks or released cultivars occupied leading GYT positions because of their balanced performance across multiple desirable traits [53–55]. Therefore, advancing the most promising experimental hybrids based on the GYT superiority index enabled the prioritization of entries that combined high grain yield with other desirable traits exhibiting significant hybrid effects, thereby supporting more efficient parent selection and cultivar advancement than selection based on grain yield alone [56,57].

## Conclusions

The study assessed the agronomic performance and variability among newly developed FAW-resistant maize hybrids under the minor cropping season, revealing significant differences among genotypes for grain yield and related agronomic traits. These differences demonstrate the presence of exploitable genetic variability that can be used for improvement of maize productivity under FAW pressure. The association analysis showed that grain yield was significantly related to several agronomic traits, indicating that yield performance is influenced by a combination of contributing characteristics rather than a single trait. This highlights the importance of considering multiple traits in hybrid evaluation and selection. Key traits influencing hybrid performance were identified, providing insight into the primary drivers of yield variation among the tested hybrids. These traits can serve as reliable selection criteria in future breeding efforts targeting improved productivity and FAW resistance. Overall, some of the evaluated hybrids consistently demonstrated superior agronomic performance and yield potential, suggesting their suitability for further advancement through the breeding pipeline. However, these promising hybrids should undergo additional multi-location and on-farm trials before recommendation for commercial cultivation to ensure stability and adaptability across diverse environments.
