# Supplementary material for: Assessment of agronomic traits in fall armyworm (Spodoptera frugiperda) resistant maize (Zea mays) hybrids grown in the derived savanna agro-ecology of Nigeria
Source: PLoS One. 2026 Jun 26;21(6):e0352140. doi: 10.1371/journal.pone.0352140 (PMC13308806; doi:10.1371/journal.pone.0352140)

PLOS ONE

Assessment of agronomic traits in fall armyworm (Spodoptera frugiperda) resisistant maize hybrids grown in the derived savanna agro-ecology of Nigeria

--Manuscript Draft--

**Manuscript Number:**	PONE-D-25-64535
**Article Type:**	Research Article
**Full Title:**	Assessment of agronomic traits in fall armyworm (Spodoptera frugiperda) resisistant maize hybrids grown in the derived savanna agro-ecology of Nigeria
**Short Title:**	Agronomic traits of FAW-resistant maize in Nigeria’s derived savanna
**Corresponding Author:**	Adesike Oladoyin Olayinka<br>Ladoke Akintola University of Technology Faculty of Agricultural Sciences<br>Ibadan, Nigeria NIGERIA
**Keywords:**	Fall armyworm resistance; maize hybrids; trait selection; variation; Genotype by yield*trait biplot.
**Abstract:**	Maize production in Nigeria has increased since 2010, but fall armyworm remains a major constraint. To address this, 26 fall armyworm-resistant maize hybrids and six commercial checks were obtained from the International Institute of Tropical Agriculture (IITA), Ibadan. Their agronomic performance was evaluated using an 8 × 4 α-lattice design with three replications at teaching and research farms of Ladoke Akintola University of Technology, Ogbomoso, and Ajayi Crowther University, Oyo, Nigeria, within the derived savanna agroecological zone of southwest Nigeria. Agronomic data were collected at key growth stages and analyzed using R statistical software v4.4.2. Significant ($p < 0.001$) phenotypic differences were observed among hybrids for grain yield, ear height, plant aspect, ear aspect, southern corn leaf blight, and FAW damage at 8 and 12 weeks. Notably, FAWSYN-1/(TZLComp. 1 C6-W-39-1-1)-B-B), Oba Super 11, FAWSYN-2/(TZLComp. 1 C6-W-39-1-1)-B-B, FAWSYN-1/IITATZI2305, and FAWSYN-1/TZISTR1121 exhibited superior performance, combining high yield with desirable agronomic traits and low FAW damage. FAWSYN-1/(TZLComp. 1 C6-W-39-1-1)-B-B) followed closely by FAWSYN-2/(TZLComp. 1 C6-W-39-1-1)-B-B and FAWSYN-1/IITATZI2305 recorded the highest grain yield across environments. These superior hybrids are recommended for further multi-environment testing across diverse agro-ecologies in Nigeria before their release to farmers.
**Order of Authors:**	Olufemi Stephen Akande, M.Tech
	Adesike Oladoyin Olayinka
	Moses Adeolu Adebayo
**Additional Information:**	
**Question**	**Response**
**Financial Disclosure**<br><br>Enter a financial disclosure statement that describes the sources of funding for the work included in this submission. Review the submission guidelines for detailed requirements. View published research articles from *PLOS ONE* for specific examples.<br><br>This statement is required for submission and **will appear in the published article** if the submission is accepted. Please make	The author(s) received no specific funding for this work.

sure it is accurate.<br><br>**Funded studies**<br>Enter a statement with the following details:<br>• Initials of the authors who received each award<br>• Grant numbers awarded to each author<br>• The full name of each funder<br>• URL of each funder website<br>• Did the sponsors or funders play any role in the study design, data collection and analysis, decision to publish, or preparation of the manuscript?<br><br>Did you receive funding for this work?	
**Competing Interests**<br><br>Use the instructions below to enter a competing interest statement for this submission. On behalf of all authors, disclose any competing interests that could be perceived to bias this work—acknowledging all financial support and any other relevant financial or non-financial competing interests.<br><br>This statement is required for submission and **will appear in the published article** if the submission is accepted. Please make sure it is accurate and that any funding sources listed in your Funding Information later in the submission form are also declared in your Financial Disclosure statement.<br><br>View published research articles from *PLOS ONE* for specific examples.	The authors have declared that no competing interests exist.

**NO authors have competing interests**<br><br>Enter: *The authors have declared that no competing interests exist.*<br><br>**Authors with competing interests**<br><br>Enter competing interest details beginning with this statement:<br><br>*I have read the journal's policy and the authors of this manuscript have the following competing interests: [insert competing interests here]*<br><br>* typeset	
**Ethics Statement**<br><br>Enter an ethics statement for this submission. This statement is required if the study involved:<br><br>• Human participants<br>• Human specimens or tissue<br>• Vertebrate animals or cephalopods<br>• Vertebrate embryos or tissues<br>• Field research<br><br>Write "N/A" if the submission does not require an ethics statement.<br><br>General guidance is provided below. Consult the submission guidelines for detailed instructions. **Make sure that all information entered here is included in the Methods section of the manuscript.**	N/A

Powered by Editorial Manager® and ProduXion Manager® from Aries Systems Corporation

**Format for specific study types**<br><br>**Human Subject Research (involving human participants and/or tissue)**<br>• Give the name of the institutional review board or ethics committee that approved the study<br>• Include the approval number and/or a statement indicating approval of this research<br>• Indicate the form of consent obtained (written/oral) or the reason that consent was not obtained (e.g. the data were analyzed anonymously)<br><br>**Animal Research (involving vertebrate animals, embryos or tissues)**<br>• Provide the name of the Institutional Animal Care and Use Committee (IACUC) or other relevant ethics board that reviewed the study protocol, and indicate whether they approved this research or granted a formal waiver of ethical approval<br>• Include an approval number if one was obtained<br>• If the study involved *non-human primates*, add *additional details* about animal welfare and steps taken to ameliorate suffering<br>• If anesthesia, euthanasia, or any kind of animal sacrifice is part of the study, include briefly which substances and/or methods were applied<br><br>**Field Research**<br><br>Include the following details if this study involves the collection of plant, animal, or other materials from a natural setting:<br>• Field permit number<br>• Name of the institution or relevant body that granted permission	
**Data Availability**<br><br>Authors are required to make all data underlying the findings described fully available, without restriction, and from the time of publication. PLOS allows rare exceptions to address legal and ethical concerns. See the PLOS Data Policy and FAQ for detailed information.	Yes - all data are fully available without restriction

A Data Availability Statement describing where the data can be found is required at submission. Your answers to this question constitute the Data Availability Statement and **will be published in the article**, if accepted.<br><br>**Important:** Stating 'data available on request from the author' is not sufficient. If your data are only available upon request, select 'No' for the first question and explain your exceptional situation in the text box.<br><br>Do the authors confirm that all data underlying the findings described in their manuscript are fully available without restriction?	
**Describe where the data may be found in full sentences. If you are copying our sample text, replace any instances of XXX with the appropriate details.**<br><br>• If the data are **held or will be held in a public repository**, include URLs, accession numbers or DOIs. If this information will only be available after acceptance, indicate this by ticking the box below. For example: *All XXX files are available from the XXX database (accession number(s) XXX, XXX.)*.<br>• If the data are all contained **within the manuscript and/or Supporting Information files**, enter the following: *All relevant data are within the manuscript and its Supporting Information files.*<br>• If neither of these applies but you are able to provide **details of access elsewhere**, with or without limitations, please do so. For example:<br><br>*Data cannot be shared publicly because of [XXX]. Data are available from the XXX Institutional Data Access / Ethics Committee (contact via XXX) for researchers who meet the criteria for access to confidential data.*<br><br>*The data underlying the results presented in the study are available from (include the name of the third party*	The data underlying the results presented in the study are available on request to the corresponding Author

Powered by Editorial Manager® and ProduXion Manager® from Aries Systems Corporation

and contact information or URL).<br>• This text is appropriate if the data are owned by a third party and authors do not have permission to share the data.<br><br>* typeset	
Additional data availability information:	

Powered by Editorial Manager® and ProduXion Manager® from Aries Systems Corporation

Manuscript

Click here to access/download;Manuscript;PLOS ONE MANUSCRIPT.docx

Assessment of agronomic traits in fall armyworm (*Spodoptera frugiperda*) resistant maize hybrids grown in the derived savanna agro-ecology of Nigeria

Akande, O.S.[1], Olayinka, A. O.[1*], Adebayo, M.A.[2]

[1]Crop Production and Soil Science Department, Ladoke Akintola University of Technology, PMB 4000, Ogbomoso, Nigeria.

[2]Department of Crop and Animal Science, Ajayi Crowther University, P.M.B. 1066, Oyo, Oyo State, Nigeria.

[*]Corresponding author

Email: aokolawole@lautech.edu.ng (AO)

Abstract

Maize production in Nigeria has increased since 2010, but fall armyworm remains a major constraint. To address this, 26 fall armyworm-resistant maize hybrids and six commercial checks were obtained from the International Institute of Tropical Agriculture (IITA), Ibadan. Their agronomic performance was evaluated using an 8 × 4 α-lattice design with three replications at teaching and research farms of Ladoke Akintola University of Technology, Ogbomoso, and Ajayi Crowther University, Oyo, Nigeria., within the derived savanna agroecological zone of southwest Nigeria. Agronomic data were collected at key growth stages and analyzed using R statistical software v4.4.2. Significant ($p < 0.001$) phenotypic differences were observed among hybrids for grain yield, ear height, plant aspect, ear aspect, southern corn leaf blight, and FAW damage at 8 and 12 weeks. Notably, FAWSYN-1/(TZLComp. 1 C6-W-39-1-1)-B-B), Oba Super 11, FAWSYN-2/(TZLComp. 1 C6-W-39-1-1)-B-B, FAWSYN-1/IITATZI2305, and FAWSYN-1/TZISTR1121 exhibited superior performance, combining high yield with desirable agronomic traits and low FAW damage. FAWSYN-1/(TZLComp. 1 C6-W-39-1-1)-B-B) followed closely by FAWSYN-2/(TZLComp. 1 C6-W-39-1-1)-B-B and FAWSYN-1/IITATZI2305 recorded the highest grain yield across environments. These superior hybrids are recommended for further multi-environment testing across diverse agro-ecologies in Nigeria before their release to farmers.

Introduction

Maize (*Zea mays* L.) is one of the most widely cultivated and important cereal crops globally, serving as a staple food for millions and a vital component in animal feed and industrial applications (1). Annual global production has surpassed 1147 million tons (2). Its

adaptability to diverse agro-climatic conditions enables cultivation across various regions, with leading producers including the United States, China, Brazil, Argentina, and India (3). It remained a key food source and economic livelihood for approximately 300 million smallholder farmers across sub-Saharan Africa (4). Maize production in Nigeria reached 12.75 million metric tons in 2021 (5).

Despite its critical role, maize production in Nigeria faces multiple constraints, especially biotic stresses such as insect pests and diseases, which cause significant yield reductions. Among these, the fall armyworm (*Spodoptera frugiperda*), an invasive polyphagous pest, has become a major threat since its detection in West and Central Africa in 2016 (6). The pest feeds voraciously on maize leaves and stalks, leading to weakened plants, increased vulnerability to secondary infections, and substantial yield losses. Fall armyworm infestations have been associated with annual economic losses estimated between US$ 2.5 billion and 6.2 billion in affected African countries (7). For instance, Ethiopia experienced a 36% average annual yield loss between 2017 and 2019 attributed to this pest, equating to a US$ 200 million economic setback (8). Similarly, maize yields in Nigeria declined by over 40% due to fall armyworm damage (9) underscoring the global impact of this pest.

FAW invasion in Africa has led to increased pesticide use among smallholder farmers, raising concerns about environmental and health risks. Studies show that synthetic pesticides remain the most popular management option, with usage increasing up to 3-fold in some areas (10). Consequently, breeding maize hybrids with enhanced resistance or tolerance to fall armyworm has emerged as a sustainable strategy to mitigate crop losses and reduce pesticide dependency. Institutions such as the International Institute of Tropical Agriculture (IITA) and the International Maize and Wheat Improvement Center (CIMMYT) have made substantial progress in developing fall armyworm-resistant maize varieties using a combination of traditional breeding and molecular tools (11).

The derived savanna agro-ecology of southwestern Nigeria is an important maize-growing region characterized by bimodal rainfall patterns and moderate temperatures conducive to maize cultivation. However, its climatic conditions also favour fall armyworm proliferation, posing significant challenges to maize productivity in this zone (12). While several resistant hybrids have been developed, their performance and adaptability within the derived savanna environment remain understudied. Systematic evaluation of these hybrids for yield, pest resistance, and agronomic traits is essential to guide selection and distribution.

Thus, the objectives of this study are to: (i) assess the agronomic performance and variation among fall armyworm-resistant maize hybrids, (ii) investigate the association between grain yield and agronomic traits, (iii) identify key traits influencing hybrid performance, and (iv) select superior maize hybrids with enhanced yield and fall armyworm tolerance.

Materials and methods

Plant materials, experimental site, and cultivation

The plant materials evaluated in this study consisted of twenty-six fall armyworm-resistant maize hybrids and six commercial checks obtained from the International Institute of Tropical Agriculture (IITA), Ibadan, Nigeria (Table 1). These hybrids represent genetically diverse materials bred specifically for tolerance or resistance to fall armyworm (*Spodoptera frugiperda*), alongside commonly used commercial varieties for comparative purposes.

Table 1. Fall armyworm-resistant synthetic females top-cross maize hybrid and checks evaluated in 2024.

Entry ID	Genotype	Source
**G1**	FAWSYN-1/(TZLComp. 1 C6-W-39-1-1)-B-B	IITA
**G2**	FAWSYN-1/TZISTR1869	IITA
**G3**	FAWSYN-1/TZISTR1878	IITA
**G4**	FAWSYN-1/TZISTR1121	IITA
**G5**	FAWSYN-1/TZISTR1129	IITA
**G6**	FAWSYN-1/TZISTR2024	IITA
**G7**	FAWSYN-1/TZISTR1305	IITA
**G8**	FAWSYN-1/TZISTR2042	IITA
**G9**	FAWSYN-2/(TZLComp. 1 C6-W-39-1-1)-B-B	IITA
**G10**	FAWSYN-2/TZISTR1878	IITA
**G11**	FAWSYN-2/TZISTR1129	IITA
**G12**	FAWSYN-2/TZISTR2024	IITA
**G13**	FAWSYN-2/TZISTR1305	IITA
**G14**	FAWSYN-2/TZISTR2129-2	IITA
**G15**	FAWSYN-3/TZISTR1869	IITA
**G16**	FAWSYN-3/TZISTR1872	IITA
**G17**	FAWSYN-3/TZISTR1878	IITA
**G18**	FAWSYN-3/IITATZI2300	IITA
**G19**	FAWSYN-3/IITATZI2305	IITA
**G20**	FAWSYN-1/IITATZI2300	IITA
**G21**	FAWSYN-2/IITATZI2300	IITA
**G22**	FAWSYN-1/IITATZI2305	IITA
**G23**	FAWSYN-2/IITATZI2305	IITA
**G24**	FAWSYN-1	IITA
**G25**	FAWSYN-2	IITA
**G26**	FAWSYN-3	IITA
**G27**	SAMMAZ 51	IITA
**G28**	Oba Super 11	IITA
**G29**	SC301	IITA
**G30**	Oba Super 7	IITA
**G31**	Oba Super 9	IITA
**G32**	Oba Super II	IITA

Field trials were conducted in 2024 at two environments within the Derived savanna agro-ecology of southwestern Nigeria, the teaching and research farms of Ladoke Akintola University of Technology (LAUTECH), Ogbomoso (8.172°N, 4.274°E), and Ajayi Crowther University (ACU), Oyo (7.734°N, 4.064°E). Both environments experience bimodal rainfall with an average annual precipitation ranging between 1,000 and 1,200 mm and mean temperatures of 28 to 30°C during the maize growing season. Six months of weather data from May – October, 2024 is shown in Fig 1. These climatic conditions, typical of the Derived savanna zone, provide an ideal environment for assessing the adaptability and pest resistance of maize hybrids under natural fall armyworm pressure.

Fig 1. The weather data for May to October, 2024. Source: weather station, Faculty of Agricultural Sciences, LAUTECH, Ogbomoso.

The experimental fields were prepared by plough[illegible] and har[illegible]ing two weeks before planting to optimize soil conditions. The maize hybrids were planted in July 2024 during the main rainy season under rainfed conditions. The trials employed an 8x[illegible]lpha-lattice design with three replicates. Each plot meas[illegible] 4 meters in length with row spacing of 0.75 meters and intra-row spacing of 0.5 meters, establishing a plant population density of approximately 53,333 plants per hectare after thinning. Standard agronomic practices, including fertilizer application and weed control, were follo[illegible] to ensure optimal crop growth.

Data collection

Data collection focused on key agronomic traits, foliar diseases, and fall armyworm damage (FAWD) at critical maize growth stage[illegible]o evaluate the performance of the maize hybrids under study. Phenological traits including days to pollen shed and days to silking were recorded as the number of days from planting until 50% of plants within each plot reached

these reproductive stages. The anthesis-silking interval was calculated by subtracting days to pollen from days to silking, providing insight into flowering synchrony, which affects grain yield (13).

Plant height and ear height were measured using a meter rule from the base of the plant to the first tassel branch and to the first node bearing the ear, respectively. Measurements were taken on ten representative plants per plot to calculate average values (13). Plant and ear aspects were scored on a 1 to 5 scale, where a lower score indicates better visual quality and plant health. Husk cover was similarly scored, assessing the protection of the ear from pests and environmental damage. Ear rot was evaluated using a 1 to 5 scale, with 1 indicating no rot and 5 severe infection (13).

Foliar diseases evaluated included Maize Streak Virus (MSV), Southern Corn Leaf Rust (SCLR), Southern Corn Leaf Blight (SCLB), and Curvularia Leaf Spot (CLS). Each disease was scored on a 1 to 5 scale based on symptom severity, where 1 represented no visible symptoms and 5 severe infection (13). Fall armyworm damage was assessed at 4, 8, and 12 weeks after sowing using a modified Davis scale ranging from 1 (no visible damage) to 9 (severe damage with heavily destroyed leaves) (14). This allowed monitoring of pest pressure progression during critical vegetative and reproductive growth stages.

Grain yield data were obtained by harvesting ears from each plot, calculating total grain weight, and adjusting to a standard moisture content of 15%. A shelling percentage of 80% was assumed to convert ear weight to grain yield per hectare. Grain moisture content was measured using a digital moisture meter (13).

Statistical data analysis

All collected data was entered into Microsoft Excel 2019 before analysis. Analysis of variance (ANOVA) was performed using the Multi-environment Trials Analysis (metan)

package in R version 4.4.2 (15) to examine differences among the hybrids [illegible]d significant differences were verified with the Fisher's protected Least Significant Differences (LSD) test at 5% probability level. The ANOVA model included fixed effects for genotypes and environments, and random effects for blocks and replicates nested within environments, expressed as yijk = μ + ek + bj(k) + αi + (eα)ik + eijk, where yijk is the observed value of a response variable for the i-th hybrid, measured in the j-th block within the k-th environment, μ is the overall mean, bj(k) is the is the block within environment, αi is the effect of the i-th hybrid, (eα)ik is the interaction effect between the i-th hybrid and the k-th environment, and eijk is the residual error term.

Variance components for genotypic variance σ_g^2, genotype-by-environment interaction variance (σ_{ge}^2), residual error variance (σ_e^2), and broad sense heritability (H^2) were analysed using META-R v6.0 (16).

[illegible]incipal Component Analysis (PCA) was calculated using "prcomp" function in R version 4.4.2 to reduce [illegible]ensionality and identify the most significant traits contributing to genetic variation among hybrids. Those PCs with Eigenvalues >1 were selected (17).

Pearson's correlation analysis was computed to determine associations among all traits measured using the "*metan*" package in R (15).

Genotype-by-yield*trait (GYT) biplot was analysed in R 4.4.2 to simultaneously evaluate multiple traits in relation to grain yield for superiority index ranking of hybrids based on multi-trait performance (18).

Results

Analysis of variance

The analysis of variance is presented in Table 2, which indicated highly significant hybrid effects for grain yield ($p < 0.001$), ear height ($p < 0.01$), plant aspect, and ear aspect ($p < 0.05$). No significant variation was observed for plant height, husk cover, ears per plant, and ear rot. Among the foliar diseases, only southern corn leaf blight demonstrated a significant hybrid effect ($p < 0.01$). Fall armyworm damage exhibited significant variation among hybrids at 8 and 12 weeks after sowing ($p < 0.05$), but not at 4 weeks. The coefficients of variation (CV) were below 20% for traits such as plant height (14.07%) and ears per plant (14.78%), indicating data consistency. In contrast, grain yield, husk cover, and ear rot displayed CVs exceeding 20%, suggesting greater variability, potentially attributable to environmental factors or measurement subjectivity. The coefficient of determination (R^2) was high for most traits, including 0.78 for grain yield, indicating that the model effectively captured the observed variability.

Table 2. Mean squares of grain yield, foliar diseases and other agronomic trats of fall armyworm resistant maize hybrid and checks evaluated across environments

Source of Variation	Df	GY (kg/ha)	PH (cm)	EH (cm)	HC (1-5)	PASP (1-5)	EPP	EASP (1-5)	EROT (1-5)
**Environment**	1	157331.98	905055.83***	200494.29***	23.31***	6.75***	3.57***	0.29	2.88*
**Replicate (Environment)**	4	8640746.71***	3377.46***	500.18***	2.80***	3.10***	0.14***	1.90***	1.30*
**Block(Environment*Replicate)**	42	749234**	262.63***	111.83***	0.80	0.60**	0.02	0.28	0.51
**Hybrid**	31	944986***	154	77**	0.44	0.56*	0.03	0.45*	0.47
**Hybrid*Environment**	31	458715	163*	77**	0.58	0.50*	0.03	0.48*	0.43
**Error**	82	361893	98.20	36.44	0.54	0.31	0.02	0.28	0.44
**R^2**		0.78	0.99	0.99	0.70	0.73	0.82	0.68	0.65
**CV (%)**		27.67	14.07	18.20	23.72	16.16	14.78	16.50	21.02

Source of Variation	Df	MSV (1-5)	SCLR (1-5)	SCLB (1-5)	CLS (1-5)	FAWD4 (1-9)	FAWD8 (1-9)	FAWD12 (1-9)
**Environment**	1	2.88*	1.88*	35.88***	232.98***	8.33**	180.19***	292.55***
**Replicate (Environment)**	4	0.08	0.77*	2.18**	0.10	8.60***	11.13***	3.29**
**Block(Environment*Replicate)**	42	0.48	0.33	0.75*	0.27	1.13	1.92**	1.62**
**Hybrid**	31	0.42	0.36	0.93**	0.63	1.28	1.57*	1.42*
**Hybrid*Environment**	31	0.46	0.38	1.03**	0.48	1.39	1.56*	1.12
**Error**	82	0.52	0.29	0.45	0.33	1.01	0.93	0.86
**R^2**		0.53	0.65	0.79	0.64	0.68	0.84	0.91
**CV (%)**		45.17	29.94	29.24	80.98	28.08	32.65	23.69

GY = Grain yield (kg/ha); PH = Plant height (cm); EH = Ear height (cm); HC = Husk cover; PASP = Plant aspect; EPP = Ears per plant; EASP = Ear aspect; EROT = Ear rot; MSV = Maize streak virus severity; SCLR = Southern corn leaf rust; SCLB = Southern corn leaf blight; CLS = Curvularia leaf spot; FAWD4, FAWD8, FAWD12 = Fall armyworm damage ratings at 4, 8, and 12 weeks, df = degree of freedom, R^2 = coefficient of determination, CV (%) = coefficient of variation.

Mean performance

The mean performance of the evaluated hybrids is shown in Table 3. Grain yield ranged from 1,452.76 kg/ha in FAWSYN-2 to 3,331.71 kg/ha in FAWSYN-1/(TZLComp. 1 C6-W-39-1-1)-B-B (G1). The commercial check Oba Super 11 (G28) yielded 2,979.36 kg/ha, comparable to several resistant hybrids. Plant height varied from 62.08 cm to 81.12 cm, with G1 tallest, while ear height ranged from 27.04 cm in Oba Super II (G32) to 41.00 cm in G9. Husk cover scores (1–5) ranged from 2.50 to 4.33, with FAWSYN-2/TZISTR2042 (G8) showing the best cover, while ears per plant ranged from 0.73 to 1.05, highest in FAWSYN-3/IITATZI2300 (G18). Maize streak virus scores ranged from 1.17 in FAWSYN-3/TZISTR1869 and Oba Super 11 (G28) to 2.08 in FAWSYN-2/IITATZI2305 (G23). Southern corn leaf rust and blight were lowest in Oba Super 11 and FAWSYN-3/TZISTR1872, respectively. Curvularia leaf spot varied from 2.08 in Oba Super II to 3.75 in G8. Fall armyworm damage ranged from 2.00 in G1 and G28 to 4.50 in Oba Super II at 4 weeks; at 8 weeks it was lowest in G1 and G28 (2.00) and highest in SC301 (G29) (4.00), while at 12 weeks it ranged from 2.50 in FAWSYN-1/TZISTR2024 to 4.17 in FAWSYN-2.

Table 3. Mean performance of FAW-resistant maize hybrids and checks for grain yield, agronomic traits, foliar diseases and FAW infestation scores across environments.

HYBRID	GY (kg/ha)	PH (cm)	EH (cm)	HC (1-5)	PASP (1-5)	EPP	EASP (1-5)	EROT (1-5)	MSV (1-5)	SCLR (1-5)	SCLB (1-5)	CLS (1-5)	FAWD4 (1-9)	FAWD8 (1-9)	FAWD12 (1-9)
**FAWSYN-1/(TZLComp. 1 C6-W-39-1-1)-B-B**	3127.84	81.12	40.85	3.58	3.17	0.93	2.92	3.00	1.25	2.00	2.17	3.58	3.00	2.00	3.33
**FAWSYN-1/TZISTR1869**	1968.11	75.06	33.27	3.00	3.67	0.78	3.33	2.67	1.75	1.67	2.17	3.33	3.50	2.83	3.00
**FAWSYN-1/TZISTR1878**	2193.70	74.98	36.53	2.83	3.67	0.90	3.33	3.17	1.92	1.50	2.67	3.33	4.33	3.50	4.00
**FAWSYN-1/TZISTR1121**	2577.07	68.17	30.91	3.00	3.08	0.93	3.08	2.67	2.17	1.75	2.25	3.50	3.50	2.50	2.67
**FAWSYN-1/TZISTR1129**	1739.74	63.53	30.92	3.42	3.75	0.73	2.92	2.75	1.58	1.50	2.00	2.83	3.50	3.17	3.67
**FAWSYN-1/TZISTR2024**	2197.74	66.60	28.94	3.00	3.42	0.89	3.58	3.42	1.75	1.50	1.33	3.33	2.83	3.33	2.50
**FAWSYN-1/TZISTR1305**	1640.63	63.78	33.78	3.58	4.33	0.83	3.00	2.58	1.58	1.75	2.58	3.33	3.33	2.50	3.00
**FAWSYN-1/TZISTR2042**	2420.24	70.85	37.69	2.50	3.17	1.04	3.50	3.17	1.58	2.00	2.17	3.75	2.67	2.50	2.33
**FAWSYN-2/(TZLComp. 1 C6-W-39-1-1)-B-B**	2842.11	78.49	41.00	2.83	3.25	0.95	3.08	3.08	1.25	2.17	2.33	3.50	3.33	3.33	3.17
**FAWSYN-2/TZISTR1878**	2117.46	74.06	34.69	3.00	3.17	0.94	3.50	3.25	1.83	1.58	2.58	3.17	3.50	2.83	3.00
**FAWSYN-2/TZISTR1129**	2172.43	62.08	27.45	3.00	3.58	0.90	2.92	2.58	1.42	1.58	2.42	3.33	3.83	3.17	3.67
**FAWSYN-2/TZISTR2024**	1929.36	74.46	35.06	3.25	3.83	0.88	3.25	3.42	1.75	1.75	1.75	3.17	3.50	2.83	3.00
**FAWSYN-2/TZISTR1305**	1740.76	65.10	32.73	2.83	4.00	0.87	2.83	2.75	1.83	1.92	2.58	3.42	3.17	2.67	3.17
**FAWSYN-2/TZISTR2129-2**	2025.88	67.01	34.59	3.50	3.67	0.89	3.00	3.00	1.67	1.83	2.75	3.25	3.17	2.50	2.67
**FAWSYN-3/TZISTR1869**	2200.63	69.75	35.48	3.00	3.83	0.96	3.25	3.08	1.17	1.75	2.33	3.08	3.67	3.17	3.17
**FAWSYN-3/TZISTR1872**	1639.52	63.86	28.61	3.25	4.00	0.82	3.08	2.75	1.75	1.75	2.08	3.50	2.83	4.00	3.50
**FAWSYN-3/TZISTR1878**	1924.39	72.89	35.98	3.33	3.75	0.83	3.25	2.58	1.75	1.75	2.92	3.33	4.50	3.67	2.67
**FAWSYN-3/IITATZI2300**	2409.35	74.27	33.35	2.75	3.33	1.05	3.08	3.33	1.92	1.75	2.25	3.50	3.17	2.33	2.50
**FAWSYN-3/IITATZI2305**	2632.29	73.75	35.99	2.58	3.58	0.96	3.08	3.08	1.42	1.50	2.25	3.58	3.33	2.67	3.33
**FAWSYN-1/IITATZI2300**	2323.15	75.75	31.99	3.00	3.25	0.93	2.92	3.08	1.83	2.00	2.17	3.58	3.17	2.67	2.83
**FAWSYN-2/IITATZI2300**	2343.79	75.09	31.42	3.17	3.58	0.93	3.17	3.17	1.67	2.25	2.33	3.33	3.17	2.67	2.83
**FAWSYN-1/IITATZI2305**	2717.11	68.82	30.86	3.08	3.33	0.97	2.75	2.75	1.92	2.25	3.17	3.25	3.67	2.50	2.50
**FAWSYN-2/IITATZI2305**	2152.97	64.47	28.95	2.92	3.67	0.88	2.83	2.83	2.08	1.58	2.92	3.50	3.50	2.83	3.83
**FAWSYN-1**	1767.63	67.19	30.07	3.25	4.00	0.83	2.92	2.83	1.58	2.17	2.00	3.42	3.50	2.67	3.50
**FAWSYN-2**	1452.76	63.35	29.05	2.75	3.58	0.94	3.42	3.33	1.33	1.83	2.00	3.08	3.33	3.17	4.17
**FAWSYN-3**	2048.60	69.47	34.54	3.33	3.75	0.92	3.67	2.92	1.67	1.42	1.75	3.50	3.83	3.17	3.00
**SAMMAZ 51**	2236.18	68.57	33.91	2.92	3.75	0.94	3.33	2.67	1.17	1.92	2.58	3.67	4.00	3.67	3.17
**Oba Super 11**	2979.36	73.56	32.90	2.83	3.25	0.93	3.25	3.33	1.83	1.33	1.92	2.08	3.67	2.00	3.00
**SC301**	1902.07	75.76	29.51	3.07	3.58	0.92	4.00	3.42	1.50	2.00	2.67	3.50	4.33	4.00	3.50
**Oba Super 7**	2161.48	75.38	35.76	2.92	3.83	0.99	3.17	3.08	1.33	1.58	2.50	3.42	3.83	2.67	4.00
**Oba Super 9**	2438.78	71.99	37.67	2.92	3.58	0.93	3.17	3.42	1.42	1.92	2.50	3.33	3.83	3.00	3.83
**Oba Super II**	1928.39	64.87	27.04	3.42	4.08	0.97	3.33	3.25	1.25	1.58	1.75	2.58	4.50	3.50	3.33
**LSD (0.05)**	945.77	87.52	41.56	1.03	0.83	0.25	0.68	0.80	0.81	0.66	1.09	1.53	1.32	1.87	1.97

GY = Grain yield (kg/ha); PH = Plant height (cm); EH = Ear height (cm); HC = Husk cover; PASP = Plant aspect; EPP = Ears per plant; EASP = Ear aspect; EROT = Ear rot; MSV = Maize streak virus severity; SCLR = Southern corn leaf rust; SCLB = Southern corn leaf blight; CLS = Curvularia leaf spot; FAWD4, FAWD8, FAWD12 = Fall armyworm damage ratings at 4, 8, and 12 weeks, LSD (0.05): Least Significant difference at 5% probability level

Variance Components and Heritability

Variance component analysis (Table 4) ~~revealed~~ that phenotypic variance exceeded genotypic variance ~~across~~ all traits, with grain yield ~~showing~~ the highest genotypic variance (44,159.79) and phenotypic variance (387,203.12), ~~indicating moderate genetic variability among hybrids.~~ Grain yield had moderate broad-sense heritability (44%), with PCV of 14.56% and GCV of 9.61%. Other traits such as plant height, ear height, husk cover, plant and ear aspects, and ears per plant showed low or negligible genotypic variance and heritability, often close to zero. Curvularia leaf spot and fall armyworm damage at 12 weeks recorded GCV values of 4.74% and 4.21%, respectively. PCV values were ~~consistently~~ higher than GCV, ~~confirming strong environmental effects.~~

Table 4. Variance components ~~of~~ grain yield, foliar diseases, FAW ratings, and other agronomic traits across environments.

Trait	σ^2g	σ^2ge	σ^2e	σ^2p	GCV %	PCV %	H^2
**Grain yield (kg/ha)**	44159.79	0.00	343043.33	387203.12	9.61	14.56	0.44
**Plant height (cm)**	0.00	7.36	116.41	123.76	0.00	6.82	0.00
**Ear height (cm)**	0.06	7.97	38.87	46.90	0.75	9.78	0.01
**Husk cover (1-5)**	0.00	0.00	0.55	0.55	0.00	9.88	0.00
**Plant aspect (1-5)**	0.00	0.03	0.31	0.34	0.00	7.08	0.00
**Ears per plant (count)**	0.00	0.00	0.02	0.02	1.91	6.77	0.08
**Ear aspect (1-5)**	0.00	0.05	0.26	0.31	0.00	8.19	0.00
**Ear rot (1-5)**	0.00	0.00	0.40	0.40	1.83	8.76	0.04
**Maize streak virus (1-5)**	0.00	0.00	0.47	0.47	0.00	17.19	0.00
**Southern corn leaf rust (1-5)**	0.00	0.02	0.30	0.32	0.00	14.00	0.00
**Southern corn leaf blight (1-5)**	0.00	0.16	0.47	0.63	0.00	17.22	0.00
**Curvularia leaf spot (1-5)**	0.02	0.06	0.30	0.39	4.74	9.76	0.24
**FAW damage at 4 weeks (1-9)**	0.01	0.05	0.94	1.00	2.07	12.22	0.03
**FAW damage at 8 weeks (1-9)**	0.00	0.03	0.83	0.87	0.00	13.42	0.00
**FAW damage at 12 weeks (1-9)**	0.02	0.00	0.83	0.85	4.21	12.44	0.11

σ^2g: Genotypic variance; σ^2p: Phenotypic variance; σ^2e: Environmental variance; GCV %: Genotypic Coefficient of Variation; PCV %: Phenotypic Coefficient of Variation; H^2: Heritability (broad-sense).

Principal Component Analysis

Principal component analysis (PCA) revealed key traits driving variability among maize hybrids (Table 5). The first five components explained 71% of the variation. PC1 (26%) was associated with positive loadings for plant aspect, husk cover, and fall armyworm damage at 8 weeks, and negative loadings for grain yield, plant height, and ears per plant. PC2 (16%) showed positive contributions from ear aspect, ear rot, and fall armyworm damage, but negative loadings for maize streak virus, southern corn leaf blight, and rust. PC3 (12%) was dominated by southern corn leaf blight, curvularia leaf spot, and ear height, with maize streak virus negative. PC4 (9%) and PC5 (8%) highlighted curvularia leaf spot, husk cover, and rust.

Table 5. Principal component analysis of the contributions of grain yield, foliar diseases and other agronomic traits to total variation among the maize hybrids and checks evaluated across environments.

	**Eigenvectors**				
**Traits**	**PC1**	**PC2**	**PC3**	**PC4**	**PC5**
**Grain yield**	**-0.42**	-0.04	-0.01	**-0.29**	-0.09
**Plant height (cm)**	**-0.37**	0.13	0.22	-0.21	0.07
**Ear height (cm)**	**-0.31**	0.00	**0.31**	**-0.28**	0.22
**Husk cover (1-5)**	0.24	-0.17	0.02	**-0.34**	**0.38**
**Plant aspect (1-5)**	**0.41**	-0.01	0.15	-0.06	0.20
**Ears per plant (count)**	**-0.36**	0.20	0.04	0.14	-0.12
**Ear aspect (1-5)**	-0.01	**0.51**	0.02	0.27	0.00
**Ear rot (1-5)**	-0.21	**0.45**	-0.16	0.05	0.11
**Maize streak virus (1-5)**	-0.02	**-0.26**	**-0.36**	0.13	**-0.47**
**Southern corn leaf rust (1-5)**	-0.16	**-0.25**	**0.33**	**0.26**	**0.25**
**Southern corn leaf blight (1-5)**	-0.02	**-0.27**	**0.44**	-0.06	**-0.50**
**Curvularia leaf spot (1-5)**	-0.12	-0.21	**0.36**	**0.51**	0.09
**FAW damage at 4 weeks (1-9)**	0.18	**0.27**	**0.31**	**-0.32**	**-0.42**
**FAW damage at 8 weeks (1-9)**	**0.27**	**0.29**	**0.29**	**0.30**	-0.08
**FAW damage at 12 weeks (1-9)**	0.22	0.21	0.24	-0.17	-0.07
**Eigenvalue**	1.97	1.55	1.36	1.16	1.13
**Proportion (%)**	26	16	12	9	8
**Cumulative**	26	42	54	63	71

PC = principal component, Bolded values contributed higher to variation.

The biplot is ~~visualized~~ in Fig 2, showing clustering of related traits, such as fall armyworm damage at different weeks, indicating consistent behaviour across genotypes. Grain yield was ~~positioned~~ opposite to pest damage traits, ~~reaffirming~~ their negative correlation. Hybrids ~~like~~ G1, G8, and G9 clustered near the grain yield vector, ~~identifying them as high-performing genotypes with favourable agronomic and resistance traits.~~

Figure 2. PCA biplot illustrating the relationship between PC1 and PC2 for contributions of grain yield, foliar diseases and other agronomic traits to total variation among the maize hybrids and checks evaluated across environments.

Correlation Analysis

The strength and direction of linear relationships between traits showed that grain yield had a strong positive correlation with plant height ($r = 0.60$, $p < 0.001$) and moderate positive correlations with ear height ($r = 0.50$, $p < 0.01$) and ears per plant ($r = 0.53$, $p < 0.01$) (Fig. 3). Plant height correlated strongly with ear height ($r = 0.68$, $p < 0.001$) and ~~moderately negatively~~ with plant aspect ($r = -0.49$, $p < 0.01$). Ears per plant correlated positively with ear rot ($r = 0.51$, $p < 0.01$) but negatively with husk cover ($r = -0.52$, $p < 0.01$) and plant aspect ($r = -0.48$, $p < 0.01$). Grain yield was strongly and negatively associated with plant aspect ($r = -0.73$, $p < 0.001$) and moderately with fall armyworm damage at 8 weeks ($r = -0.49$, $p < 0.01$). Husk cover ~~showed~~ a positive correlation with plant aspect ($r = 0.42$, $p < 0.05$), while ear aspect correlated positively with ear rot ($r = 0.50$, $p < 0.01$) and fall armyworm damage at 8 weeks ($r = 0.47$, $p < 0.01$). Fall armyworm damage at 4 weeks correlated positively with damage at 8 weeks ($r = 0.46$, $p < 0.01$) and 12 weeks ($r = 0.34$, $p < 0.05$), while damage at 12 weeks correlated negatively with maize streak virus ($r = -0.35$, $p < 0.05$).

Fig 3. Correlogram showing the relationship between traits of fall armyworm-resistant hybrids evaluated across environments. The cell value denotes correlation coefficient (r)

values. GY = grain yield (kg/ha), PH = plant height (cm), EH = ear height (cm). HC = husk cover (1-5), PASP = plant aspect (1-5), EPP = ears per plant (count), EASP = ear aspect (1-5), EROT = ear rot (1-5), MSV = maize streak virus (1-5), SCLR = southern corn leaf rust (1-5), SCLB = southern corn leaf blight (1-5), CLS = curvularia leaf spot (1-5), FAWD4 = fall armyworm damage at 4 weeks (1-9), FAWD8 = fall armyworm damage at 8 weeks (1-9), FAWD12 = fall armyworm damage at 12 weeks (1-9).

Genotype by yield*trait analysis and superiority index

The genotype by yield*trait (GYT) analysis integrated yield with agronomic traits to rank maize hybrids based on performance across productivity and pest resistance traits. Standardized GYT values (Table 6) and the visualized GYT biplot (Fig 4) assessed yield and traits including ear height, plant aspect, husk cover, and fall armyworm damage at 8 and 12 weeks. Five hybrids emerged as top performers based on superiority indices (SI): FAWSYN-1/(TZLComp. 1 C6-W-39-1-1)-B-B (G1), Oba Super 11 (G28), FAWSYN-2/(TZLComp. 1 C6-W-39-1-1)-B-B (G9), FAWSYN-1/IITATZI2305 (G22), and FAWSYN-1/TZISTR1121 (G4). G1 achieved the highest SI of 2.32, showing superiority across yield-traits. The bottom hybrids: FAWSYN-2 (G25), FAWSYN-3/TZISTR1872 (G16), FAWSYN-1/TZISTR1129 (G5), FAWSYN-1/TZISTR1305 (G7), and SC301 (G29) underperformed in yield-trait combinations.

Table 6: Standardized genotype by yield*trait and superiority index of FAWR maize hybrids evaluated across environments.

Hybrid	Pedigree	GY*EH	GY/PASP	GY/EASP	GY/SCLB	GY/FAWD8	GY/FAWD12	SI
G1	FAWSYN-1/(TZLComp. 1 C6-W-39-1-1)-B-B	2.87	2.38	2.56	1.87	3.01	1.22	2.32
G28	Oba Super 11	1.31	1.93	1.51	2.31	2.72	1.51	1.88
G9	FAWSYN-2/(TZLComp. 1 C6-W-39-1-1)-B-B	2.28	1.66	1.55	0.97	0.28	1.01	1.29
G22	FAWSYN-1/IITATZI2305	0.56	1.28	1.99	-0.47	1.18	2.01	1.09
G4	FAWSYN-1/TZISTR1121	0.34	1.41	0.97	0.68	0.96	1.37	0.95
G19	FAWSYN-3/IITATZI2305	1.13	0.76	1.09	0.78	0.80	0.43	0.83
G8	FAWSYN-1/TZISTR2042	0.95	0.95	-0.01	0.57	0.72	1.75	0.82
G18	FAWSYN-3/IITATZI2300	0.38	0.68	0.60	0.38	0.97	1.36	0.73
G20	FAWSYN-1/IITATZI2300	0.06	0.63	0.70	0.39	0.35	0.59	0.45
G6	FAWSYN-1/TZISTR2024	-0.51	0.17	-0.54	2.69	-0.46	0.91	0.38
G21	FAWSYN-2/IITATZI2300	0.02	0.24	0.32	0.12	0.38	0.63	0.29
G31	Oba Super 9	0.98	0.41	0.52	0.00	0.13	-0.38	0.28
G15	FAWSYN-3/TZISTR1869	0.26	-0.27	-0.10	-0.13	-0.32	-0.07	-0.11
G10	FAWSYN-2/TZISTR1878	0.01	0.33	-0.59	-0.62	-0.12	-0.01	-0.17
G27	SAMMAZ 51	0.14	-0.13	-0.15	-0.44	-0.65	-0.01	-0.21
G14	FAWSYN-2/TZISTR2129-2	-0.16	-0.41	-0.12	-0.95	0.12	0.27	-0.21
G26	FAWSYN-3	-0.13	-0.45	-0.90	0.78	-0.51	-0.13	-0.22
G30	Oba Super 7	0.22	-0.34	-0.07	-0.44	0.12	-0.89	-0.23
G11	FAWSYN-2/TZISTR1129	-0.71	-0.07	0.35	-0.30	-0.36	-0.62	-0.28
G12	FAWSYN-2/TZISTR2024	-0.29	-0.73	-0.67	0.51	-0.38	-0.35	-0.32
G3	FAWSYN-1/TZISTR1878	0.37	-0.12	-0.23	-0.61	-0.59	-0.85	-0.34
G23	FAWSYN-2/IITATZI2305	-0.57	-0.19	0.45	-0.95	-0.08	-0.78	-0.35
G2	FAWSYN-1/TZISTR1869	-0.41	-0.51	-0.69	-0.27	-0.33	-0.28	-0.41
G17	FAWSYN-3/TZISTR1878	-0.21	-0.66	-0.68	-1.26	-0.98	0.07	-0.62
G32	Oba Super II	-1.11	-0.93	-0.77	0.51	-0.88	-0.69	-0.64
G24	FAWSYN-1	-1.06	-1.12	-0.58	-0.36	-0.45	-1.08	-0.78
G13	FAWSYN-2/TZISTR1305	-0.85	-1.16	-0.53	-1.20	-0.49	-0.84	-0.85
G5	FAWSYN-1/TZISTR1129	-1.02	-0.98	-0.65	-0.42	-0.88	-1.24	-0.87
G29	SC301	-0.90	-0.55	-1.46	-1.04	-1.16	-0.88	-1.00
G7	FAWSYN-1/TZISTR1305	-0.94	-1.53	-0.98	-1.36	-0.47	-0.86	-1.02
G16	FAWSYN-3/TZISTR1872	-1.38	-1.33	-1.09	-0.75	-1.42	-1.27	-1.21
G25	FAWSYN-2	-1.63	-1.36	-1.80	-0.99	-1.23	-1.91	-1.49
Mean		0.00	0.00	0.00	0.00	0.00	0.00	
Standard deviation		1.00	1.00	1.00	1.00	1.00	1.00	

GY: grain yield; DP: days to pollination; DS: days to silking; ASI: anthesis-silking interval; EH: ear height; HC: husk-cover; PASP: plant aspect; SCLB: southern corn leaf blight; CLS: curvularia leaf spot; FAWD8: fall armyworm damage at 8 week

Fig 4. The Average Tester Coordination view of the FAWR maize hybrids evaluated across environments. The biplot was based on the singular value decomposition of trait-standardized data ("Scaling=1, Centering=2") and genotype-focused singular value partition ("SVP=2"). The trait codes are: YLD: grain yield (kg/ha), EH: ear height (cm); PASP: plant aspect (1-5); EASP: ear aspect (1-5); FAWD8: fall armyworm damage at 8 weeks (1-9); FAWD12: fall armyworm damage at 12 weeks (1-9); SCLB: southern corn leaf blight (1-5).

Discussion

The 32 FAWR maize hybrids evaluated in this study performed differently in grain yield, ear height, plant aspect, ear aspect, southern corn leaf blight, and fall armyworm damage at 8 and 12 weeks across environments. Maize hybrids and open-pollinated varieties are more vulnerable to FAW damage at early growth stages but can recover in later stages, while inbred lines show increasing damage over time (19). Traits such as husk cover, ear rot resistance, and plant height correlate with FAW tolerance (20).

The significant hybrid differences observed in traits such as grain yield, ear height, plant aspect, and ear aspect underscore the importance of these characteristics in maize breeding. Specifically, ear aspect has been consistently identified as a major trait contributing directly to grain yield, alongside ears per plant, which also impacts yield positively (21–23). In contrast, the absence of significant differences in traits like plant height, husk cover, and ears per plant among fall armyworm resistance hybrids suggests that these traits may not exhibit the variability necessary for selection, indicating a potential limitation in trait diversity for breeding purposes (24,25). This aligns with findings that show positive associations between yield and certain traits, while others do not exhibit such relationships (26,27).

Variance component analysis revealed that environmental factors play a dominant role in trait expression, as indicated by the consistently higher phenotypic compared to genotypic variance across most traits (28). A broad-sense heritability of 0.44 for maize grain yield classified as moderate (29) suggests that approximately 44% of the variation in maize grain yield is due to genetic differences among the plants, and the remaining 56% is influenced by environmental factors (like rainfall, soil fertility, temperature) and other non-genetic factors (30). For most other traits, low heritability estimates highlight the challenge of achieving genetic gains through conventional selection alone, emphasizing the need for multi-location testing and the integration of genomic selection approaches.

Principal component analysis (PCA) further clarified the relationships among traits, with the first five components explaining a substantial 71% proportion of total variation. The negative association between grain yield and pest damage traits in PC1 reflects the classic trade-off between productivity and stress tolerance, reinforcing the challenge of breeding for both high yield and resilience (18).

Correlation analysis revealed that key yield components, such as plant height and ears per plant, are positively associated with grain yield (31), while negative correlations between yield and both plant aspect and FAW damage highlight the detrimental effects of poor plant architecture and pest pressure (32). These relationships underscore the importance of multi-trait selection indices that can balance yield improvement with enhanced resistance and optimal plant type (33).

The genotype by yield*trait (GYT) analysis provided a holistic ranking of hybrids, integrating yield with agronomic and resistance traits (18). The identification of superior hybrids such as G1 and G28 demonstrates the success of both experimental and commercial breeding efforts in delivering genotypes with balanced performance.

Conclusions

The results from this study emphasize the extent of variability among fall armyworm resistant maize hybrids, especially on grain yield, ear height, and plant and ear aspects. These differences offer valuable opportunities for breeders to select hybrids with strong overall performance. While some traits ~~like~~ ears per plant and ear aspect ~~show~~ clear connections to higher yield, others, such as plant height and husk cover, didn't vary as much among the hybrids, which may limit their inclusion in selection criteria. The moderate heritability of grain yield suggests that both genetics and the environment play major roles, indicating that selection efforts need to account for performance across different environments. Interestingly, the conflicts observed between yield and pest resistance reinforce the ongoing challenge of breeding for both productivity and resilience. The principal component analysis and GYT analysis clarified hybrids G1 and G28 performed excellently for their balance of agronomic strength and stress tolerance, showing that it is possible to breed maize that performs well without compromising on resilience. Going forward, breeding programs will benefit from combining traditional selection with genomic tools and multi-trait approaches to develop more adaptable and high-performing maize varieties.

Acknowledgement

We appreciate the International Institute of Tropical Agriculture, (IITA) Ibadan, Nigeria for providing the fall armyworm resistant maize hybrids used in this study.

References

1. Erenstein O, Jaleta M, Sonder K, Mottaleb K, Prasanna BM. Global maize production, consumption and trade: trends and R&D implications. Food Secur. 2022 Oct;14(5):1295–319.

2. Yang N, Yan J. New genomic approaches for enhancing maize genetic improvement. Curr Opin Plant Biol. 2021 Apr;60:101977.

3. Klein HS, Luna FV. Maize. In: Brazilian Crops in the Global Market [Internet]. Cham: Springer Nature Switzerland; 2023 [cited 2025 July 30]. p. 107–29. (Palgrave Studies in Economic History). Available from: https://link.springer.com/10.1007/978-3-031-38589-6_5

4. Badu-Apraku B, Fakorede MAB. Maize in Sub-Saharan Africa: Importance and Production Constraints. In: Advances in Genetic Enhancement of Early and Extra-Early Maize for Sub-Saharan Africa [Internet]. Cham: Springer International Publishing; 2017 [cited 2025 July 30]. p. 3–10. Available from: http://link.springer.com/10.1007/978-3-319-64852-1_1

5. Statista. Statista. 2023 [cited 2025 Mar 2]. Nigeria: maize production quantity. Available from: https://www.statista.com/statistics/1300743/production-volume-of-maize-in-nigeria/

6. Goergen G, Kumar PL, Sankung SB, Togola A, Tamò M. First Report of Outbreaks of the Fall Armyworm Spodoptera frugiperda (J E Smith) (Lepidoptera, Noctuidae), a New Alien Invasive Pest in West and Central Africa. Luthe DS, editor. PLOS ONE. 2016 Oct 27;11(10):e0165632.

7. Day R, Abrahams P, Bateman M, Beale T, Clottey V, Cock M, et al. Fall Armyworm: Impacts and Implications for Africa. Outlooks Pest Manag. 2017 Oct 1;28(5):196–201.

8. Abro Z, Kimathi E, De Groote H, Tefera T, Sevgan S, Niassy S, et al. Socioeconomic and health impacts of fall armyworm in Ethiopia. Nagoshi RN, editor. PLOS ONE. 2021 Nov 4;16(11):e0257736.

9. Utono IM, Adamu RS. Effect of belt expert (Flubendiamide + Thiacloprid), Imidacloprid, Thiamethoxam seed treatment and economic impact on fall armyworm (*Spodoptera frugiperda*) infestation on Maize in Nigeria. Cogent Food Agric. 2023 Dec 31;9(1):2164117.

10. Tambo JA, Kansiime MK, Mugambi I, Agboyi LK, Beseh PK, Day R. Economic impacts and management of fall armyworm (Spodoptera frugiperda) in smallholder agriculture: a panel data analysis for Ghana. CABI Agric Biosci. 2023 Oct 2;4(1):38.

11. Krishna VV, Lantican MA, Prasanna BM, Pixley K, Abdoulaye T, Menkir A, et al. Impact of CGIAR maize germplasm in Sub-Saharan Africa. Field Crops Res. 2023 Jan;290:108756.

12. Adunola MP, Fayeun LS, Fadara AB. Impact of climate change on armyworm infestation on maize in Nigeria: A review. J Plant Breed Crop Sci. 2021 Sept 30;13(3):158–67.

13. Badu-Apraku B, Fakorede MAB, Menkir A, Sanogo D. Conduct and management of maize field trials. 2012 [cited 2025 July 30]; Available from: https://cgspace.cgiar.org/bitstreams/81fda619-5d11-4b6f-9885-bf75ba268cc7/download

14. Matova PM, Kamutando CN, Magorokosho C, Kutywayo D, Gutsa F, Labuschagne M. Fall-armyworm invasion, control practices and resistance breeding in Sub-Saharan Africa. Crop Sci. 2020 Nov;60(6):2951–70.

15. Olivoto T, Lúcio AD. metan: An R package for multi-environment trial analysis. Jarman S, editor. Methods Ecol Evol. 2020 June;11(6):783–9.

16. Alvarado G, Rodríguez FM, Pacheco A, Burgueño J, Crossa J, Vargas M, et al. META-R: A software to analyze data from multi-environment plant breeding trials. Crop J. 2020;8(5):745–56.

17. Jolliffe IT, Cadima J. Principal component analysis: a review and recent developments. Philos Trans R Soc Math Phys Eng Sci. 2016 Apr 13;374(2065):20150202.

18. Yan W, Frégeau-Reid J. Genotype by yield* trait (GYT) biplot: a novel approach for genotype selection based on multiple traits. Sci Rep. 2018;8(1):8242.

19. Kenyi JJ, Mengesha W, Abe A, Menkir A, Meseka S. Variation in grain yield losses due to fall armyworm infestation among elite open-pollinated maize varieties under different levels of insecticide application. Agriculture. 2024;14(7):984.

20. Matova PM, Kamutando CN, Kutywayo D, Magorokosho C, Labuschagne M. Fall armyworm tolerance of maize parental lines, experimental hybrids, and commercial cultivars in Southern Africa. Agronomy. 2022;12(6):1463.

21. Badu-Apraku B, Magorokosho C, Fasanmade Y, Fakorede MAB, Talabi AO, Asiedu R, et al. Yield gains and associated changes in an early yellow bi-parental maize population following genomic selection for Striga resistance and drought tolerance. BMC Plant Biol. 2019 Apr 5;19(1).

22. Nasser LM, Badu-Apraku B, Gracen VE, Mafouasson HNA. Combining Ability of Early-Maturing Yellow Maize Inbreds under Combined Drought and Heat Stress and Well-Watered Environments. Agronomy. 2020 Oct 16;10(10):1585.

23. Amegbor IK, Van Biljon A, Shargie N, Tarekegne A, Labuschagne MT. Heritability and Associations among Grain Yield and Quality Traits in Quality Protein Maize (QPM) and Non-QPM Hybrids. Plants. 2022 Mar 8;11(6):713.

24. Darshan R, Prasanna P M, Hegde JN. Screening of Popular Maize Hybrids against fall Armyworm, Spodoptera frugiperda. J Exp Agric Int. 2024 Mar 21;46(5):306–12.

25. Sultana R. MOLECULAR AND MORPHOLOGICAL CHARACTERIZATION OF MAIZE GENOTYPES FOR OPAQUE 2 GENE AND YIELD CONTRIBUTING TRAITS. Big Data Agric. 2019 Jan 12;1(1):07–14.

26. Bhadmus OA, Badu-Apraku B, Adeyemo OA, Ogunkanmi AL. Genetic Analysis of Early White Quality Protein Maize Inbreds and Derived Hybrids under Low-Nitrogen and Combined Drought and Heat Stress Environments. Plants. 2021 Nov 26;10(12):2596.

27. Badu-Apraku B, Adu GB, Yacoubou AM, Toyinbo J, Adewale S. Gains in Genetic Enhancement of Early Maturing Maize Hybrids Developed during Three Breeding Periods under Striga-Infested and Striga-Free Environments. Agronomy. 2020 Aug 13;10(8):1188.

28. Alam M, Rahman M, Ahmed S, Jahan N, Khan M, Islam M, et al. Genetic Variation and Genotype by Environment Interaction for Agronomic Traits in Maize (Zea mays L.) Hybrids. Plants. 2022 June 6;11(11):1522.

29. Johnson HW, Robinson HF, Comstock RE. Estimates of genetic and environmental variability in soybeans. 1955 [cited 2025 July 31]; Available from: https://www.cabidigitallibrary.org/doi/full/10.5555/19561600791

30. Khajoane TJ. Genotype and environmental effects on maize grain yield, nutritional value and milling quality. 2022 [cited 2025 July 31]; Available from: https://scholar.ufs.ac.za/items/8cbafbac-5082-46f7-946f-da3878877362

31. Aman J, Bantte K, Alamerew S, Sbhatu DB. Correlation and Path Coefficient Analysis of Yield and Yield Components of Quality Protein Maize (*Zea mays* L.) Hybrids at Jimma, Western Ethiopia. Int J Agron. 2020 June 8;2020:1–7.

32. Chisonga C, Chipabika G, Sohati PH, Harrison RD. Understanding the impact of fall armyworm (Spodoptera frugiperda JE Smith) leaf damage on maize yields. PLoS One. 2023;18(6):e0279138.

33. Moeinizade S, Kusmec A, Hu G, Wang L, Schnable PS. Multi-trait Genomic Selection Methods for Crop Improvement. Genetics. 2020 Aug 1;215(4):931–45.

Fig 1

Click here to access/download;Figure;Fig 1.tif

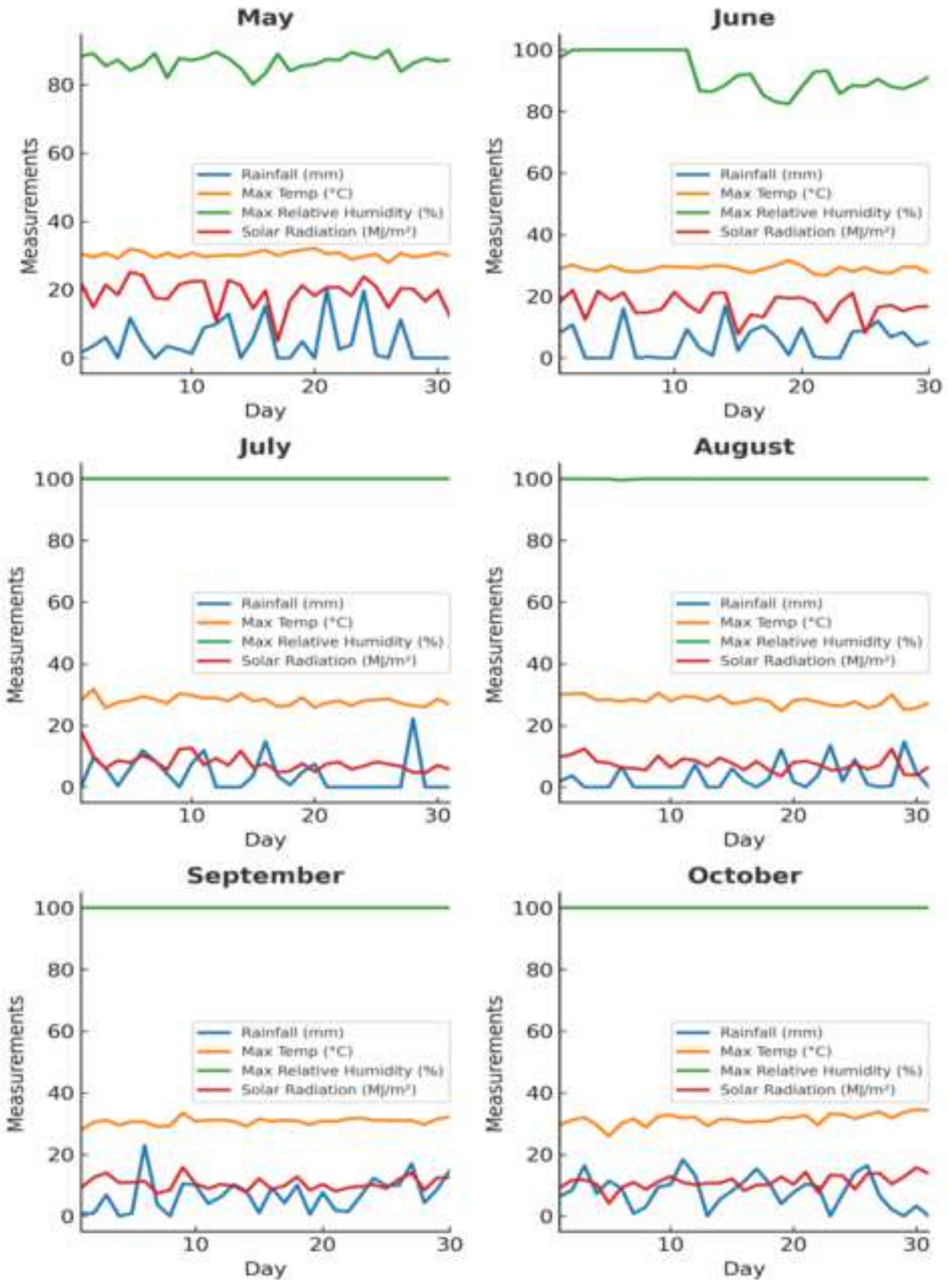

Fig 2

Click here to access/download;Figure;Fig 2.tif

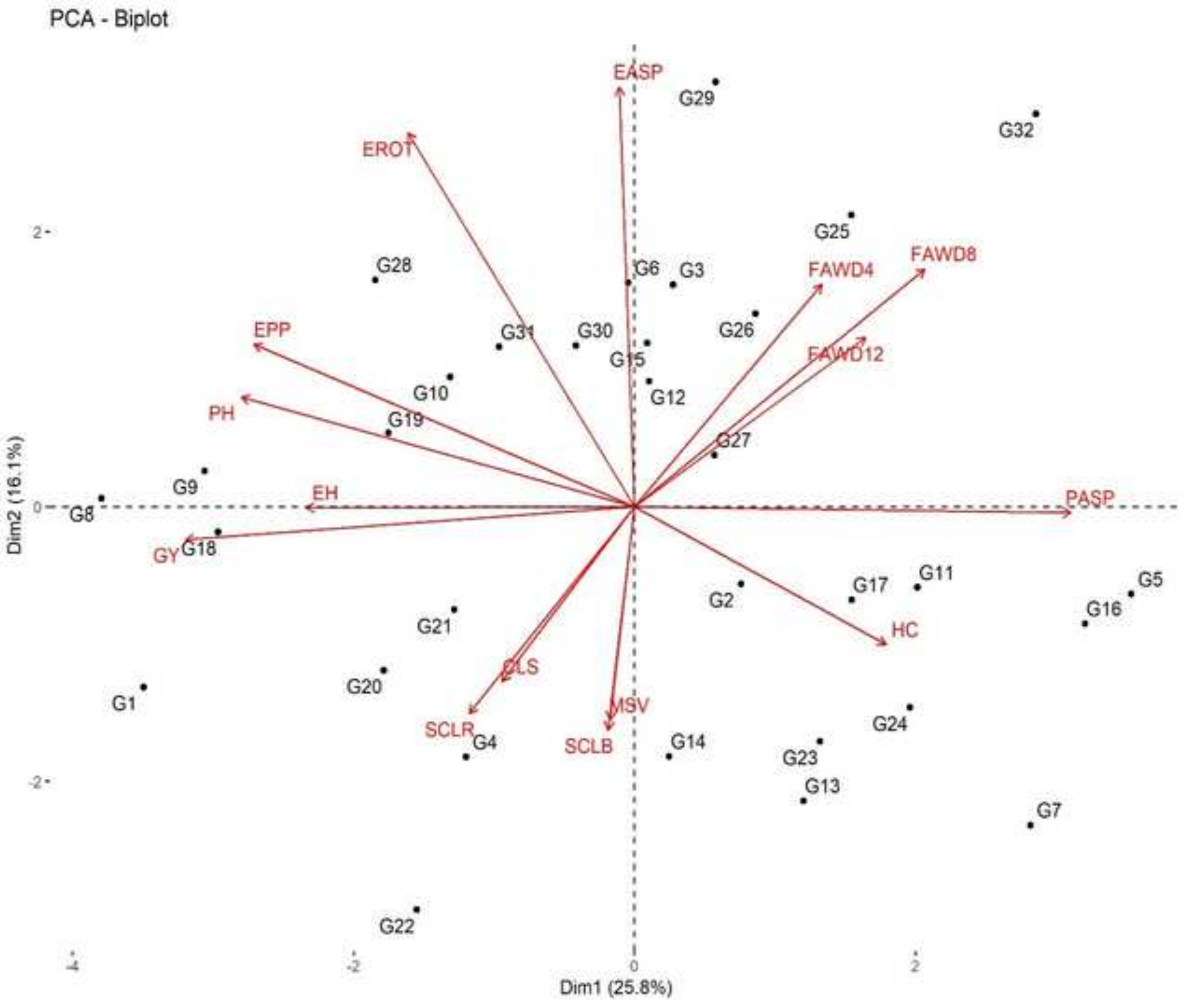

Fig 3

Click here to access/download;Figure;Fig 3.tif

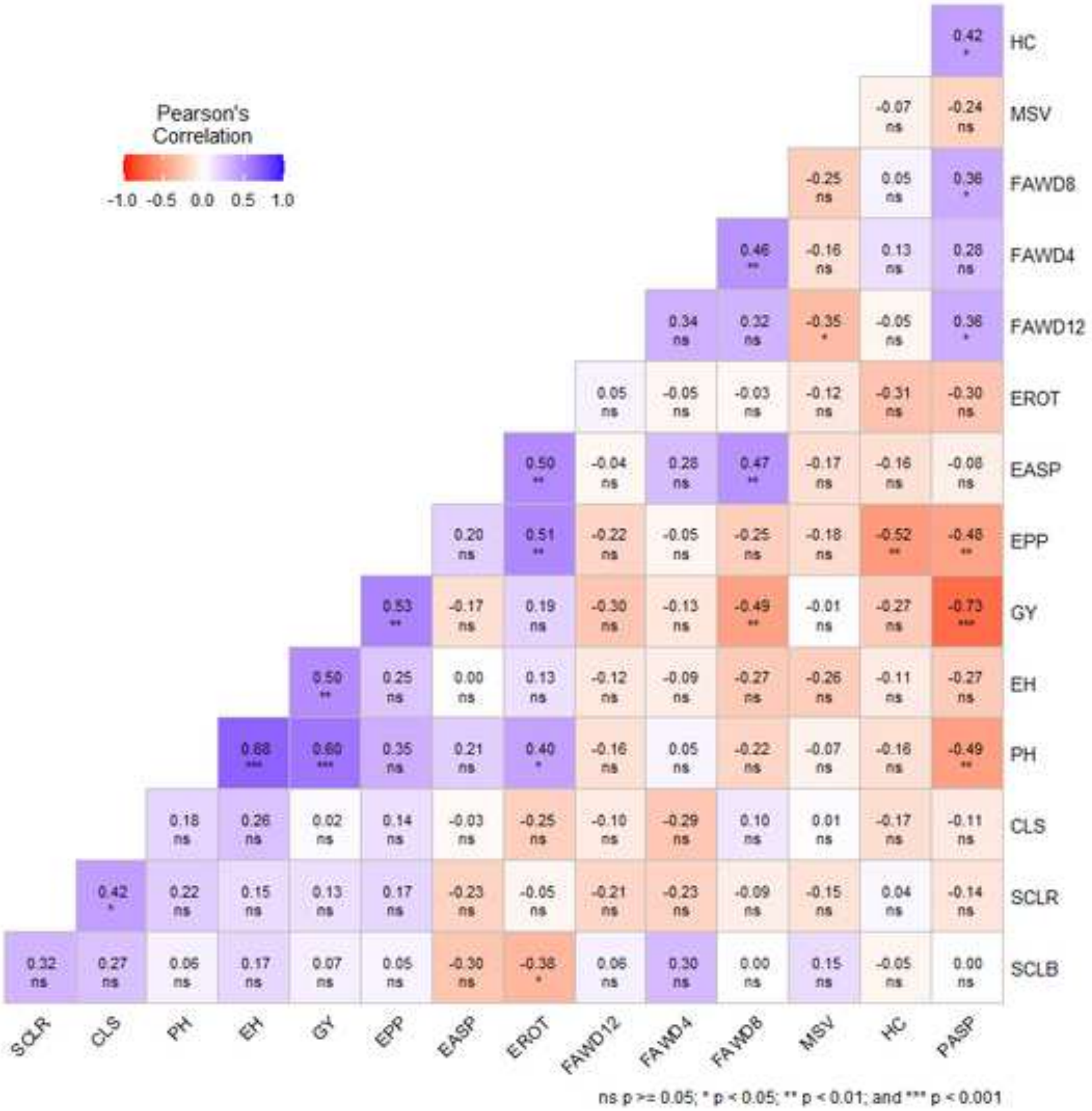

Fig 4

Click here to access/download;Figure;Fig 4.tif

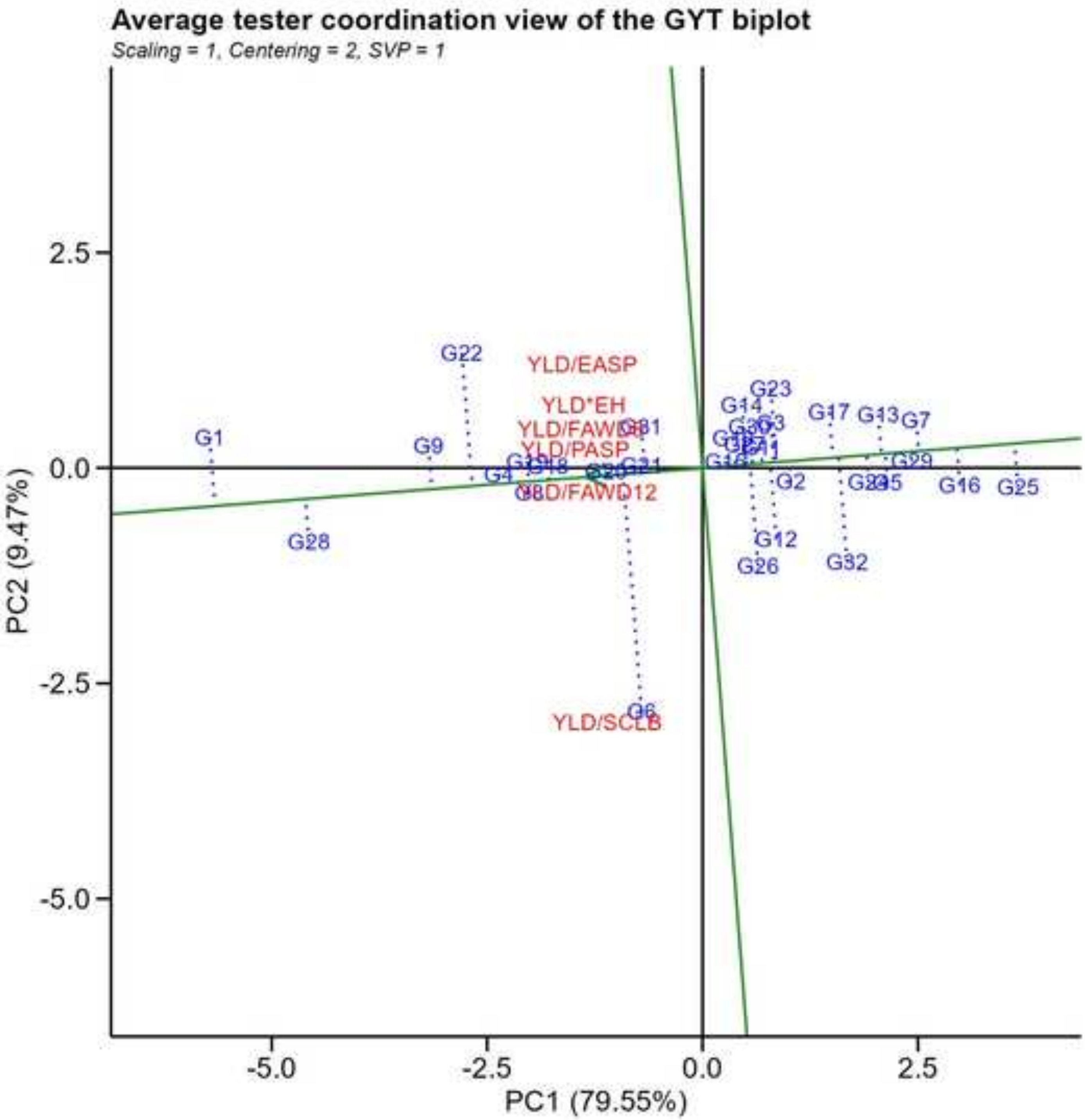

Supplement: Attachment — Submitted filename: PONE-D-25-64535_REVIEWED.pdf [file pone.0352140.s001.pdf]
