# Supplementary material for: Assessment of agronomic traits in fall armyworm (Spodoptera frugiperda) resistant maize (Zea mays) hybrids grown in the derived savanna agro-ecology of Nigeria
Source: PLoS One. 2026 Jun 26;21(6):e0352140. doi: 10.1371/journal.pone.0352140 (PMC13308806; doi:10.1371/journal.pone.0352140)
Supplement: Attachment — Submitted filename: PONE-D-25-64535_R1_REVISION.pdf [file pone.0352140.s003.pdf]

PLOS One

Assessment of agronomic traits in fall armyworm (Spodoptera frugiperda) resisistant maize (Zea mays) hybrids grown in the derived savanna agro-ecology of Nigeria

--Manuscript Draft--

**Manuscript Number:**	PONE-D-25-64535R1
**Article Type:**	Research Article
**Full Title:**	Assessment of agronomic traits in fall armyworm (Spodoptera frugiperda) resisistant maize (Zea mays) hybrids grown in the derived savanna agro-ecology of Nigeria
**Short Title:**	Agronomic traits of FAW-resistant maize in Nigeria’s derived savanna
**Corresponding Author:**	Adesike Oladoyin Olayinka<br>Ladoke Akintola University of Technology Faculty of Agricultural Sciences<br>Ibadan, Nigeria NIGERIA
**Keywords:**	Fall armyworm resistance; maize hybrids; trait selection; variation; Genotype by yield*trait biplot.
**Abstract:**	Maize production in Nigeria has increased in recent years, but fall armyworm infestation remains a major constraint to sustainable high productivity, particularly during the minor cropping season. Twenty-six fall armyworm-resistant maize hybrids and six commercial checks were evaluated for grain yield and other traits using an 8 × 4 alpha-lattice design with three replications. Data collected at key growth stages and harvesting were analyzed using R statistical software version 4.4.2. Hybrids exhibited significant ($p < 0.05$) differences for grain yield, ear height, plant aspect, ear aspect, southern corn leaf blight, and foliar fall armyworm damage at 8 and 12 weeks after planting. Notably, five experimental hybrids exhibited superior performance, combining high yield with desirable agronomic traits and low foliar fall armyworm damage. FAWSYN-1/(TZLComp. 1 C6-W-39-1-1)-B-B, followed closely by FAWSYN-1/IITATZI2305, FAWSYN-2/(TZLComp. 1 C6-W-39-1-1)-B-B, FAWSYN-1/TZISTR1121, and FAWSYN-3/IITATZI2305 recorded the highest grain yields across environments. When further tested for performance stability across more diverse agro-ecologies, the selected hybrids may be released as maize cultivars to mitigate fall armyworm attacks among Nigerian farmers.
**Order of Authors:**	Olufemi Stephen Akande, M.Tech<br>Adesike Oladoyin Olayinka<br>Moses Adeolu Adebayo
**Response to Reviewers:**	We have revised the manuscript thoroughly as follows:<br>Comment 1: Please specify the type of fall armyworm damage evaluated (leaf, stalk, or ear damage).<br>Response: The study evaluated foliar fall armyworm damage (leaf damage) using the modified Davis 1–9 scale at 4, 8, and 12 weeks after planting. This has now been explicitly stated in the Abstract, Materials and Methods (Data Collection section), Results tables, and figure captions to ensure clarity and consistency throughout the manuscript.<br><br>Comment 2: The plant height values appear questionable for tropical hybrids. Please review and correct.<br>Response: Upon careful re-examination of the raw dataset and statistical outputs, we identified a unit-of-measurement handling error during statistical processing. Field measurements were correctly recorded in centimeters; however, an error occurred during data handling that affected the reported summaries. This error has now been corrected, and all affected tables and related results have been regenerated using the corrected dataset. The revised plant and ear height values are accurate and internally consistent.<br><br>Comment 3: The reported height of Oba Super II contradicts previous studies.<br>Response: Following correction of the unit-processing error described above, all genotype height values including Oba Super II were updated. The corrected values

	now reflect the properly processed field measurements for the 2024 growing season.<br><br>Comment 4: The ANOVA/mixed model description is not correct. The replicate effect is missing, and the nesting structure should reflect the alpha-lattice design.<br>Response: The Statistical Data Analysis section was revised to correctly reflect the alpha-lattice experimental design. Genotype and environment were treated as random effects. Replicates were modeled as nested within environment, and incomplete blocks were modeled as nested within replicate and environment. The genotype × environment interaction was retained within the mixed-model framework. The model description has been rewritten accordingly for accuracy and transparency.<br><br>Comment 5: Avoid interpretation in the Results section. Move explanatory statements to the Discussion.<br>Response: The Results section has been revised to present only descriptive statistical outputs, including means, ranges, significance levels, variance components, principal component loadings, and correlation coefficients. Interpretative statements and broader implications have been relocated to the Discussion section to maintain proper scientific structure.<br><br>Comment 6: Since all entries were obtained from IITA, the “Source” column in Table 1 is unnecessary.<br>Response: The “Source” column has been removed from Table 1. A clarifying statement has been added in the Materials and Methods section indicating that all hybrids and checks were obtained from the International Institute of Tropical Agriculture (IITA), Ibadan, Nigeria.<br><br>Comment 7: Avoid qualitative terms such as “highly significant.” Report significance clearly.<br>Response: The manuscript has been revised to report statistical significance strictly using probability levels ($p < 0.05$, $p < 0.01$, $p < 0.001$), without qualitative modifiers.<br><br>Comment 8: Clarify terminology (write terms in full at first use; avoid beginning sentences with acronyms).<br>Response: All statistical terms are now written in full at first mention, including Genotypic variance, Phenotypic variance, Broad-sense heritability, Genotypic Coefficient of Variation, and Phenotypic Coefficient of Variation. Sentences no longer begin with acronyms, and terminology has been standardized throughout.<br><br>Comment 9: Clarify what “Cumulative” refers to in the PCA table.<br>Response: The PCA table has been revised to read “Cumulative percentage of total variation explained” to improve clarity.<br><br>The discussion and conclusion section was also revised
**Additional Information:**	
**Question**	**Response**
**Financial Disclosure**<br><br>Enter a financial disclosure statement that describes the sources of funding for the work included in this submission. Review the submission guidelines for detailed requirements. View published research articles from *PLOS ONE* for specific examples.<br><br>This statement is required for submission and **will appear in the published article** if the submission is accepted. Please make	The author(s) received no specific funding for this work.

Powered by Editorial Manager® and ProduXion Manager® from Aries Systems Corporation

sure it is accurate.<br><br>**Funded studies**<br>Enter a statement with the following details:<br>• Initials of the authors who received each award<br>• Grant numbers awarded to each author<br>• The full name of each funder<br>• URL of each funder website<br>• Did the sponsors or funders play any role in the study design, data collection and analysis, decision to publish, or preparation of the manuscript?<br><br>Did you receive funding for this work?	
**Competing Interests**<br><br>Use the instructions below to enter a competing interest statement for this submission. On behalf of all authors, disclose any competing interests that could be perceived to bias this work—acknowledging all financial support and any other relevant financial or non-financial competing interests.<br><br>This statement is required for submission and **will appear in the published article** if the submission is accepted. Please make sure it is accurate and that any funding sources listed in your Funding Information later in the submission form are also declared in your Financial Disclosure statement.<br><br>View published research articles from *PLOS ONE* for specific examples.	The authors have declared that no competing interests exist.

**NO authors have competing interests**<br><br>Enter: *The authors have declared that no competing interests exist.*<br><br>**Authors with competing interests**<br><br>Enter competing interest details beginning with this statement:<br><br>*I have read the journal's policy and the authors of this manuscript have the following competing interests: [insert competing interests here]*<br><br>* typeset	
**Ethics Statement**<br><br>Enter an ethics statement for this submission. This statement is required if the study involved:<br><br>• Human participants<br>• Human specimens or tissue<br>• Vertebrate animals or cephalopods<br>• Vertebrate embryos or tissues<br>• Field research<br><br>Write "N/A" if the submission does not require an ethics statement.<br><br>General guidance is provided below. Consult the submission guidelines for detailed instructions. **Make sure that all information entered here is included in the Methods section of the manuscript.**	N/A

Powered by Editorial Manager® and ProduXion Manager® from Aries Systems Corporation

**Format for specific study types**<br><br>**Human Subject Research (involving human participants and/or tissue)**<br>• Give the name of the institutional review board or ethics committee that approved the study<br>• Include the approval number and/or a statement indicating approval of this research<br>• Indicate the form of consent obtained (written/oral) or the reason that consent was not obtained (e.g. the data were analyzed anonymously)<br><br>**Animal Research (involving vertebrate animals, embryos or tissues)**<br>• Provide the name of the Institutional Animal Care and Use Committee (IACUC) or other relevant ethics board that reviewed the study protocol, and indicate whether they approved this research or granted a formal waiver of ethical approval<br>• Include an approval number if one was obtained<br>• If the study involved *non-human primates*, add *additional details* about animal welfare and steps taken to ameliorate suffering<br>• If anesthesia, euthanasia, or any kind of animal sacrifice is part of the study, include briefly which substances and/or methods were applied<br><br>**Field Research**<br><br>Include the following details if this study involves the collection of plant, animal, or other materials from a natural setting:<br>• Field permit number<br>• Name of the institution or relevant body that granted permission	
**Data Availability**<br><br>Authors are required to make all data underlying the findings described fully available, without restriction, and from the time of publication. PLOS allows rare exceptions to address legal and ethical concerns. See the PLOS Data Policy and FAQ for detailed information.	Yes - all data are fully available without restriction

A Data Availability Statement describing where the data can be found is required at submission. Your answers to this question constitute the Data Availability Statement and **will be published in the article**, if accepted.<br><br>**Important:** Stating 'data available on request from the author' is not sufficient. If your data are only available upon request, select 'No' for the first question and explain your exceptional situation in the text box.<br><br>Do the authors confirm that all data underlying the findings described in their manuscript are fully available without restriction?	
**Describe where the data may be found in full sentences. If you are copying our sample text, replace any instances of XXX with the appropriate details.**<br><br>• If the data are **held or will be held in a public repository**, include URLs, accession numbers or DOIs. If this information will only be available after acceptance, indicate this by ticking the box below. For example: *All XXX files are available from the XXX database (accession number(s) XXX, XXX.*).<br>• If the data are all contained **within the manuscript and/or Supporting Information files**, enter the following: *All relevant data are within the manuscript and its Supporting Information files.*<br>• If neither of these applies but you are able to provide **details of access elsewhere**, with or without limitations, please do so. For example:<br><br>*Data cannot be shared publicly because of [XXX]. Data are available from the XXX Institutional Data Access / Ethics Committee (contact via XXX) for researchers who meet the criteria for access to confidential data.*<br><br>*The data underlying the results presented in the study are available from (include the name of the third party*	The data underlying the results presented in the study are available at https://doi.org/10.6084/m9.figshare.31385203

and contact information or URL).<br>• This text is appropriate if the data are owned by a third party and authors do not have permission to share the data.<br><br>* typeset	
Additional data availability information:	

Powered by Editorial Manager® and ProduXion Manager® from Aries Systems Corporation

Manuscript Click here to access/download;Manuscript;Manuscript.docx

Assessment of agronomic traits in fall armyworm (*Spodoptera frugiperda*) resistant maize (*Zea mays*) hybrids grown in the derived savanna agro-ecology of Nigeria

Akande, O.S.[1], Olayinka, A. O.[1*], Adebayo, M.A.[2]

[1]Crop Production and Soil Science Department, Ladoke Akintola University of Technology, PMB 4000, Ogbomoso, Oyo State, Nigeria.

[2]Department of Crop and Animal Science, Ajayi Crowther University, P.M.B. 1066, Oyo, Oyo State, Nigeria.

[*]Corresponding author

Email: aokolawole@lautech.edu.ng (AO)

Abstract

Maize production in Nigeria has increased in recent years, but fall armyworm infestation remains a major constraint to ~~sustainable~~ high productivity, particularly during the minor cropping season. Twenty-six fall armyworm-resistant maize hybrids and six commercial checks were evaluated for grain yield and other traits using an 8 × 4 alpha-lattice design with three replications. Data collected at key growth stages and harvesting were analyzed using R statistical software version 4.4.2. Hybrids exhibited significant ($p < 0.05$) differences for grain yield, ear height, plant aspect, ear aspect, southern corn leaf blight, and foliar fall armyworm damage at 8 and 12 weeks after planting. Notably, five experimental hybrids exhibited superior performance, combining high yield with desirable agronomic traits and low foliar fall armyworm damage. FAWSYN-1/(TZLComp. 1 C6-W-39-1-1)-B-B, followed closely by FAWSYN-1/IITATZI2305, FAWSYN-2/(TZLComp. 1 C6-W-39-1-1)-B-B, FAWSYN-1/TZISTR1121, and FAWSYN-3/IITATZI2305 recorded the highest grain yields across environments. When further tested for performance stability across more diverse agro-ecologies, the selected hybrids may be released as maize cultivars to mitigate fall armyworm attacks among Nigerian farmers.

Introduction

Maize (Zea mays) is one of the most widely cultivated and important cereal crops globally, serving as a staple food for millions of people and as a vital component of animal feed, forage and industrial applications (1,2). The annual global production has surpassed 1.147 billion tons (3). Its adaptability to diverse agro-climatic conditions enables cultivation across various regions, with leading producers being the United States, China, Brazil, Argentina, and India (4). However, achieving consistent productivity depends on the development and

systematic evaluation of improved hybrids, as grain yield and related agronomic traits can vary significantly among genotypes and across growing seasons, particularly under rainfed conditions (2). It remained a key source of food and economic livelihoods for approximately 300 million smallholder farmers across Sub-Saharan Africa (SSA) (5).

Maize production in Nigeria reached 12.75 million metric tons in 2021 (6). Despite its critical role in the country, the crop faces multiple constraints, particularly biotic stresses, including insect pests and diseases, which cause significant yield reductions. (7). Among these, *Spodoptera frugiperda*, commonly referred to as FAW, an invasive polyphagous pest, has become a major threat since its detection in West and Central Africa (WCA) in 2016 (8). The pest feeds voraciously on maize leaves, stalks, tassels, and ears, leading to weakened plants, increased vulnerability to secondary infections, and substantial yield losses (9). FAW infestations have been associated with annual economic losses estimated at US$ 2.5–6.2 billion in twelve affected African countries, and up to US$ 13 billion in maize, rice, sorghum, and sugarcane across SSA (10,11). For instance, Ethiopia experienced a 36% average annual yield loss between 2017 and 2019, attributed to the pest, resulting in an economic loss of US$ 200 million (12). Similarly, maize yields in Nigeria declined by over 40% due to foliar FAW damage. (13).

The invasion of FAW in Africa has led to increased pesticide use among smallholder farmers, raising concerns about environmental and health risks. Studies have shown that synthetic pesticides remain the most popular management option, with usage increasing up to 3-fold in some areas (14). However, the calamitous environmental impacts of pesticide use, coupled with the rising costs of the chemicals, which are becoming increasingly unaffordable by resource-poor farmers across SSA, have rendered this strategy unattractive (15,16). Breeding maize hybrids with enhanced resistance or tolerance to FAW has emerged as a sustainable strategy to mitigate crop losses and reduce reliance on pesticides (17,18).

Institutions such as the International Institute of Tropical Agriculture (IITA) and the International Maize and Wheat Improvement Centre (CIMMYT) have made substantial progress in developing FAW-resistant maize varieties using a combination of traditional breeding and molecular tools (19).

The derived savanna agro-ecology of southwestern Nigeria is an important maize-growing region characterized by bimodal rainfall patterns and moderate temperatures conducive to maize cultivation (2). However, its climatic conditions also favor FAW resurgence and proliferation, posing significant challenges to maize productivity in the zone. (20). While several resistant FAW maize hybrids have been developed and evaluated, their performance and adaptability within the derived savanna agro-ecology of Nigeria remain understudied (21–23). Systematic evaluation of these hybrids for yield, pest resistance, and agronomic traits is essential to guide the identification and selection of superior varieties. Thus, the objectives of this study were to: (i) assess the agronomic performance and variation among new, IITA-developed FAW-resistant maize hybrids in the minor cropping season, (ii) determine the association between grain yield and other desirable agronomic traits, (iii) identify key traits influencing hybrid performance, and (iv) select superior maize hybrids with enhanced yield and FAW tolerance.

Materials and methods

Genetic materials, experimental Site, and field establishment

The genetic materials evaluated in this study consisted of 26 FAW-resistant maize hybrids and 6 commercial checks obtained from the International Institute of Tropical Agriculture (IITA), Ibadan, Nigeria (Table 1). The 26 experimental hybrids represent genetically diverse materials bred specifically for tolerance or resistance to FAW. Field trials

were conducted in 2024 at two environments within the derived savanna agro-ecology of southwestern Nigeria at the Teaching and Research Farm of Ladoke Akintola University of Technology (LAUTECH), Ogbomoso (8.172°N, 4.274°E), and the Teaching and Research Farm of Ajayi Crowther University (ACU), Oyo (7.734°N, 4.064°E).

Table 1. List of the 26 FAW-resistant synthetic ~~females~~ top-cross maize hybrids and 6 commercial hybrid checks evaluated in two locations in the derived savanna agro-ecology ~~in the minor cropping seasons~~ of ~~2024 in~~ Nigeria.

Hybrid ID	Pedigree
**G1**	FAWSYN-1/(TZLComp. 1 C6-W-39-1-1)-B-B
**G2**	FAWSYN-1/TZISTR1869
**G3**	FAWSYN-1/TZISTR1878
**G4**	FAWSYN-1/TZISTR1121
**G5**	FAWSYN-1/TZISTR1129
**G6**	FAWSYN-1/TZISTR2024
**G7**	FAWSYN-1/TZISTR1305
**G8**	FAWSYN-1/TZISTR2042
**G9**	FAWSYN-2/(TZLComp. 1 C6-W-39-1-1)-B-B
**G10**	FAWSYN-2/TZISTR1878
**G11**	FAWSYN-2/TZISTR1129
**G12**	FAWSYN-2/TZISTR2024
**G13**	FAWSYN-2/TZISTR1305
**G14**	FAWSYN-2/TZISTR2129-2
**G15**	FAWSYN-3/TZISTR1869
**G16**	FAWSYN-3/TZISTR1872
**G17**	FAWSYN-3/TZISTR1878
**G18**	FAWSYN-3/IITATZI2300
**G19**	FAWSYN-3/IITATZI2305
**G20**	FAWSYN-1/IITATZI2300
**G21**	FAWSYN-2/IITATZI2300
**G22**	FAWSYN-1/IITATZI2305
**G23**	FAWSYN-2/IITATZI2305
**G24**	FAWSYN-1
**G25**	FAWSYN-2
**G26**	FAWSYN-3
**G27**	SAMMAZ 51
**G28**	Oba Super 11
**G29**	SC301
**G30**	Oba Super 7
**G31**	Oba Super 9
**G32**	Oba Super II

Both environments experience bimodal rainfall, with average annual precipitation ranging from 1,000 to 1,200 mm and mean temperatures of 28 to 30 °C du[illegible] the major and minor

maize-growing seasons (24). Six months of weather data observed at Ogbomoso during the study period from May – October 2024 are shown in Fi[illegible] These climatic conditions, typical of the derived savanna agro-ecological zone, provide an ideal environment for assessing the agronomic performance and pest resistance of maize hybrids under natural FAW pressure.

Fig 1. The weather data for May to October, 2024. Source: weather station, Faculty of Agricultural Sciences, LAUTECH, Ogbomoso.

The experimental field at each location was ploughed and harrowed two weeks before planting to optimize soil conditions. Experimental hybrids and checks were planted in July 2024 during the minor cropping season. Entries were laid out in an 8×4 alpha-lattice design with three replicates. Each plot was 4 m in length with row spacing of 0.75 m and intra-row spacing of 0.5 m. Three seeds were planted in each hill, and the plants were thinned to two per stand 2 weeks after planting (WAP) to attain a plant population density of 53,333 plants/ha. Standard agronomic practices, including fertilizer application and weed control, were done to ensure optimal crop growth. No pesticides were used to control FAW infestation throughout each experiment.

Data collection

Data on key agronomic traits, foliar diseases, and foliar fall armyworm damage at critical maize growth stages and at harvest were recorded in accordance with the conduct and management of maize field trials (25). Phenological traits, including days to pollen shed and days to silking, were recorded as the number of days from planting to when 50% of plants in each plot began shedding pollen and emerged with receptive silks, respectively. The anthesis-silking interval was calculated by subtracting the number of days to pollen from the number of

days to silking, providing insight into flowering synchrony, which affects grain yield (25). Plant and ear heights were measured using a meter rule from the base of the plant to the first tassel branch and to the first node bearing the upper ear, respectively. Measurements were taken on ten representative plants per plot to calculate average values. Plant aspect was rated visually on a scale of 1 to 5, where 1 = excellent overall phenotypic appearance and 5 = poor overall phenotypic appearance. Ear aspect was also scored visually on a scale of 1 to 5, where 1 = clean, uniform, large, and well-filled ears, and 5 = rotten, variable, small, and partially filled ears. Husk cover was assessed on a scale of 1 to 5, where 1 = husks tightly arranged and extended beyond the ear tip, and 5 = ear tips fully exposed. Ear rot was evaluated using a scale of 1 to 5, with 1 indicating no rot and 5 severe infection (22). Foliar diseases evaluated include maize streak virus (MSV), southern corn leaf rust (SCLR), southern corn leaf blight (SCLB), and *curvularia* leaf spot (CLS). Each disease was scored on a 1-5 scale based on symptom severity, with 1 indicating no visible symptoms and 5 indicating a severe infection (15). Foliar FAW damage was assessed at 4, 8, and 12 weeks after planting (WAP) using a modified Davis scale ranging between 1 (no visible damage) and 9 (severe damage with heavily destroyed leaves) (26). All ears were harvested per plot, field weight and grain weight were taken, and the percent grain moisture at harvest was determined using a moisture meter. Grain yield (Kg/ha) was computed using total field weight adjusted to 15% moisture content, and 80% shelling percentage(22).

$$GrainYield(kg/ha) = \left(\frac{earweight(kg/plot)}{\text{plot area (m}^2\text{)}} \times 0.80 \times \left(\frac{(100 - \text{MC})}{85}\right) \times 10{,}000\right)$$

Statistical data analysis

Analysis of variance (ANOVA) was performed using the Multi-environment Trials Analysis (metan) package in R version 4.4.2 (27) to determine the differences among the hybrids, and trait means were separated with Fisher's protected Least Significant Differences (LSD) test at

5% probability level. The ANOVA model treated all factors (genotype, environment, genotype × environment interaction, replicate within environment, and block within replicate and environment) as random effects, expressed as: $y_{ijkl} = \mu + g_i + e_j + (ge)_{ij} + r_{k(j)} + b_{l(kj)} + \varepsilon_{ijkl}$, where y_{ijkl} is the observed value of a response variable for the ith genotype evaluated in the jth environment, replicate k within environment j, and block l within replicate k and environment j; μ is the overall mean; g_i is the effect of the ith genotype; e_j is the random effect of the jth environment; $(ge)_{ij}$ is the effect of the genotype × environment interaction; $r_{k(j)}$ is the effect of replicate k nested within environment j; $b_{l(kj)}$ is the effect of block l nested within replicate k and environment j; and ε_{ijkl} is the residual error (28).

The genotypic variance σ_g^2, genotype × environment interaction variance (σ_{ge}^2), and residual error variance (σ_e^2) were estimated using META-R v6.0 (29). The Principal Component Analysis (PCA) was performed using the "prcomp" function in R version 4.4.2 to reduce the dimensionality and identify the most significant traits contributing to the variability of the hybrids. Those PCs with eigenvalues >1 were selected (30). Spearman's correlation analysis was computed to determine the associations among all measured traits using the "metan" package in R (27). Genotype by yield × trait (GYT) analysis and biplot were visualized in R 4.4.2 to evaluate multiple traits in relation to grain yield simultaneously and to rank hybrids for superiority based on multi-trait performance (31,32).

Results

Analysis of variance

Results of analysis of variance shown in Table 2 indicated significant hybrid effects ($p < 0.05$) for grain yield, plant aspect, ear aspect, southern corn leaf blight, *curvularia* leaf spot, and foliar FAW damage at 8 and 12 weeks after planting (WAP). Significant hybrid × environment

interaction effects ($p < 0.05$) were observed for plant aspect, ear aspect, southern corn leaf blight, and foliar FAW damage at 8 WAP. Environmental effects were significant ($p < 0.05$) for all trait[illegible]cept grain yield and ear aspect. The coefficients of variation (CV) were below 20% for plant aspect, ear aspect, ears per plant, and *curvularia* leaf spot. In contrast, grain yield, plant height, ear height, husk cover, ear rot, maize streak virus, southern corn leaf rust, southern corn leaf blight, and foliar FAW damage at 4, 8 and 12 WAP had CV values above 20%. The coefficients of determination (R^2) were generally moderate to high (0.54 – 0.91) for most of the traits

Mean performance

The mean performance of the evaluated hybrids is shown in Table 3. Grain yield ranged between 1,452.76 Kg/ha in FAWSYN-2 and 3,127.84 Kg/ha in FAWSYN-1/(TZLComp. 1 C6-W-39-1-1)-B-B, with a trial mean of 2,185.98 Kg/ha. Relative to the best commercial check, Oba Super 11 with 2,979.36 Kg/ha, the top-performing hybrid showed a 5% yield advantage. Plant height ranged from 139.77 cm in FAWSYN-2/TZISTR2129-2 to 309.83 cm in FAWSYN-2/IITATZI2305. Ear height ranged from 64.88 cm in FAWSYN-1 to 156.04 cm in FAWSYN-1/TZISTR1305. Husk cover scores (1–5) ranged from 2.50 in FAWSYN-1/TZISTR2042 to 3.58 in FAWSYN-1/(TZLComp. 1 C6-W-39-1-1)-B-B and FAWSYN-1/TZISTR1305. Plant aspect scores (1–5) ranged from 3.08 in FAWSYN-1/TZISTR1121 to 4.33 in FAWSYN-1/TZISTR1305. Ears per plant ranged from 0.73 in FAWSYN-1/TZISTR1129 to 1.05 in FAWSYN-3/IITATZI2300. Ear aspect scores (1–5) ranged from 2.75 in FAWSYN-1/IITATZI2305 to 4.00 in SC301. Ear rot scores (1–5) ranged from 2.58 in FAWSYN-2/TZISTR1129, FAWSYN-3/TZISTR1878, and FAWSYN-1/TZISTR1305 to 3.42 in FAWSYN-1/TZISTR2024, FAWSYN-2/TZISTR2024, SC301, and Oba Super 9. Maize streak virus scores (1–5) ranged from 1.17 in FAWSYN-3/TZISTR1869 and SAMMAZ 51 to 2.17 in

FAWSYN-1/TZISTR1121. Southern corn leaf rust scores (1–5) ranged from 1.33 in Oba Super 11 to 2.25 in FAWSYN-1/IITATZI2305 and FAWSYN-2/IITATZI2300. Southern corn leaf blight scores (1–5) ranged from 1.33 in FAWSYN-1/TZISTR2024 to 3.17 in FAWSYN-1/IITATZI2305. *Curvularia* leaf spot scores (1–5) ranged from 2.08 in Oba Super 11 to 4.50 in FAWSYN-3/TZISTR1878 and Oba Super II. Foliar FAW damage ratings (1–9) at 4 WAP ranged from 2.00 in FAWSYN-1/(TZLComp. 1 C6-W-39-1-1)-B-B, FAWSYN-1/TZISTR2042, Oba Super 11, and Oba Super 9 to 4.00 in FAWSYN-3/TZISTR1872 and SC301. At 8 WAP, it ranged from 2.67 in FAWSYN-1, FAWSYN-3/IITATZI2300, FAWSYN-1/TZISTR1305, FAWSYN-3/TZISTR1872, and Oba Super 7 to 4.00 in FAWSYN-1/TZISTR1878, SC301, and Oba Super II, while at 12 WAP, it ranged from 2.33 in FAWSYN-1/TZISTR2042 to 4.17 in FAWSYN-2.

Table 2. Mean squares of grain yield, other agronomic traits, foliar diseases, and foliar FAW damage of FAW-resistant maize hybrids and checks evaluated across two environments in the derived savanna agro-ecology of Nigeria during the minor season of 2024.

Source of Variation	df	GY (Kg/ha)	PH (cm)	EH (cm)	HC (1-5)	PASP (1-5)	EPP (count)	EASP (1-5)	EROT (1-5)
**Hybrid**	31	944986.05***	4872.88	1457.97	0.45	0.56*	0.03	0.45*	0.47
**Hybrid × Environment**	31	458714.73	5795.06	1433.73	0.58	0.50*	0.02	0.48*	0.43
**Environment**	1	157331.98	73310.09***	19528.27***	23.31***	6.75***	3.57***	0.29	2.88*
**Replicate (Environment)**	4	8640746.71***	10090.06	1118.13	2.80***	3.10***	0.14***	1.90***	1.30*
**Block (Environment × Replicate)**	42	749233.82**	5804.50	1772.87	0.80	0.6**	0.02	0.28	0.51
**Error**	82	361892.90	5417.27	1545.23	0.54	0.31	0.02	0.28	0.44
**R^2**		0.79	0.61	0.60	0.69	0.75	0.82	0.68	0.61
**CV (%)**		27.52	46.4	52.02	24.12	15.32	14.84	16.57	22.02

Source of Variation	df	MSV (1-5)	SCLR (1-5)	SCLB (1-5)	CLS (1-5)	FFAWD4 (1-9)	FFAWD8 (1-9)	FFAWD12 (1-9)
**Hybrid**	31	0.42	0.36	0.93**	0.63*	1.28	1.57*	1.42*
**Hybrid × Environment**	31	0.46	0.38	1.03**	0.48	1.39	1.59*	1.12
**Environment**	1	2.88*	1.88*	35.88***	232.98***	8.33**	180.19***	292.55***
**Replicate (Environment)**	4	0.08	0.77*	2.18**	0.10	8.60***	11.13***	3.29**
**Block (Environment × Replicate)**	42	0.48	0.33	0.75*	0.27	1.13	1.92**	1.62**
**Error**	82	0.52	0.29	0.45	0.33	1.01	0.93	0.86
**R^2**		0.54	0.64	0.79	0.91	0.68	0.84	0.87
**CV (%)**		44.49	30.2	29.18	17.29	28.49	32.85	29.09

*, **, *** = Significance at 0.05, 0.01, and 0.001 probability levels respectively; GY = grain yield; PH = plant height; EH = ear height; HC = husk cover; PASP = plant aspect; EPP = ears per plant; EASP = ear aspect; EROT = ear rot; MSV = maize streak virus; SCLR = southern corn leaf rust; SCLB = southern corn leaf blight; CLS = *curvularia* leaf spot; FFAWD4, FFAWD8, FFAWD12 = foliar FAW damage ratings at 4, 8, and 12 WAP; df = degrees of freedom; R^2 = coefficient of determination; CV (%) = coefficient of variation.

Table 3. Mean performance of FAW-resistant maize hybrids and checks for grain yield, other agronomic traits, foliar diseases, and foliar FAW damage across environments in 2024.

HYBRID	GY (Kg/ha)	PH (cm)	EH (cm)	HC (1-5)	PASP (1-5)	EPP (count)	EASP (1-5)	EROT (1-5)	MSV (1-5)	SCLR (1-5)	SCLB (1-5)	CLS (1-5)	FFAWD4 (1-9)	FFAWD8 (1-9)	FFAWD12 (1-9)
**FAWSYN-1/(TZLComp. 1 C6-W-39-1-1)-B-B**	3127.84	165.11	76.98	3.58	3.17	0.93	2.92	3.00	1.25	2.00	2.17	3.58	3.00	2.00	3.33
**FAWSYN-2/(TZLComp. 1 C6-W-39-1-1)-B-B**	2842.10	170.23	87.04	2.83	3.25	0.95	3.08	3.08	1.25	2.17	2.33	3.50	3.33	3.33	3.17
**FAWSYN-1/IITATZI2305**	2717.11	155.94	68.97	3.08	3.33	0.97	2.75	2.75	1.92	2.25	3.17	3.25	3.67	2.50	2.50
**FAWSYN-3/IITATZI2305**	2632.29	162.35	79.39	2.58	3.58	0.96	3.08	3.08	1.42	1.50	2.25	3.58	3.33	2.67	3.33
**FAWSYN-1/TZISTR1121**	2577.07	148.85	70.35	3.00	3.08	0.93	3.08	2.67	2.17	1.75	2.25	3.50	3.50	2.50	2.67
**FAWSYN-1/TZISTR2042**	2420.24	155.99	74.32	2.50	3.17	1.04	3.50	3.17	1.58	2.00	2.17	3.75	2.67	2.50	2.33
**FAWSYN-3/IITATZI2300**	2409.35	156.60	75.92	2.75	3.33	1.05	3.08	3.33	1.92	1.75	2.25	3.50	3.17	2.33	2.50
**FAWSYN-2/IITATZI2300**	2343.79	156.60	70.85	3.17	3.58	0.93	3.17	3.17	1.67	2.25	2.33	3.33	3.17	2.67	2.83
**FAWSYN-1/IITATZI2300**	2323.15	161.22	75.22	3.00	3.25	0.93	2.92	3.08	1.83	2.00	2.17	3.58	3.17	2.67	2.83
**FAWSYN-3/TZISTR1869**	2200.63	155.22	79.04	3.00	3.83	0.96	3.25	3.08	1.17	1.75	2.33	3.08	3.67	3.17	3.17
**FAWSYN-1/TZISTR2024**	2197.74	151.08	71.51	3.00	3.42	0.89	3.58	3.42	1.75	1.50	1.33	3.33	2.83	3.33	2.50
**FAWSYN-1/TZISTR1878**	2193.70	157.81	76.46	2.83	3.67	0.90	3.33	3.17	1.92	1.50	2.67	3.33	4.33	3.50	4.00
**FAWSYN-2/TZISTR1129**	2172.43	152.17	66.56	3.00	3.58	0.90	2.92	2.58	1.42	1.58	2.42	3.33	3.83	3.17	3.67
**FAWSYN-2/IITATZI2305**	2152.97	309.83	65.74	2.92	3.67	0.88	2.83	2.83	2.08	1.58	2.92	3.50	3.50	2.83	3.83
**FAWSYN-2/TZISTR1878**	2117.46	156.39	68.19	3.00	3.17	0.94	3.50	3.25	1.83	1.58	2.58	3.17	3.50	2.83	3.00
**FAWSYN-3**	2048.60	148.01	71.67	3.33	3.75	0.92	3.67	2.92	1.67	1.42	1.75	3.50	3.83	3.17	3.00
**FAWSYN-2/TZISTR2129-2**	2025.88	139.77	70.56	3.50	3.67	0.89	3.00	3.00	1.67	1.83	2.75	3.25	3.17	2.50	2.67
**FAWSYN-1/TZISTR1869**	1968.10	155.58	70.06	3.00	3.67	0.78	3.33	2.67	1.75	1.67	2.17	3.33	3.50	2.83	3.00
**FAWSYN-2/TZISTR2024**	1929.36	155.64	72.85	3.25	3.83	0.88	3.25	3.42	1.75	1.75	1.75	3.17	3.50	2.83	3.00
**FAWSYN-3/TZISTR1878**	1924.39	157.86	77.23	3.33	3.75	0.83	3.25	2.58	1.75	1.75	2.92	3.33	4.50	3.67	2.67
**FAWSYN-1**	1767.63	142.60	64.88	3.25	4.00	0.83	2.92	2.83	1.58	2.17	2.00	3.42	3.50	2.67	3.50
**FAWSYN-2/TZISTR1305**	1740.76	150.08	70.51	2.83	4.00	0.87	2.83	2.75	1.83	1.92	2.58	3.42	3.17	2.67	3.17
**FAWSYN-1/TZISTR1129**	1739.74	158.57	77.94	3.42	3.75	0.73	2.92	2.75	1.58	1.50	2.00	2.83	3.50	3.17	3.67
**FAWSYN-1/TZISTR1305**	1640.63	146.94	156.04	3.58	4.33	0.83	3.00	2.58	1.58	1.75	2.58	3.33	3.33	2.50	3.00
**FAWSYN-3/TZISTR1872**	1639.52	147.85	65.24	3.25	4.00	0.82	3.08	2.75	1.75	1.75	2.08	3.50	2.83	4.00	3.50
**FAWSYN-2**	1452.76	139.91	66.50	2.75	3.58	0.94	3.42	3.33	1.33	1.83	2.00	3.08	3.33	3.17	4.17
**Checks**															
**SAMMAZ 51**	2236.18	145.62	73.84	2.92	3.75	0.94	3.33	2.67	1.17	1.92	2.58	3.67	4.00	3.67	3.17

**Oba Super 11**	2979.36	154.74	77.28	2.83	3.25	0.93	3.25	3.33	1.83	1.33	1.92	2.08	3.67	2.00	3.00
**SC301**	1902.07	165.52	72.08	3.07	3.58	0.92	4.00	3.42	1.50	2.00	2.67	3.50	4.33	4.00	3.50
**Oba Super 7**	2161.48	152.77	77.84	2.92	3.83	0.99	3.17	3.08	1.33	1.58	2.50	3.42	3.83	2.67	4.00
**Oba Super 9**	2438.78	154.32	80.90	2.92	3.58	0.93	3.17	3.42	1.42	1.92	2.50	3.33	3.83	3.00	3.83
**Oba Super II**	1928.39	145.23	66.31	3.42	4.08	0.97	3.33	3.25	1.25	1.58	1.75	2.58	4.50	3.50	3.33
**Statistics**															
**Mean**	2185.98	158.64	75.57	3.06	3.61	0.91	3.18	3.01	1.62	1.78	2.31	3.32	3.53	2.94	3.18
**LSD(0.05)**	690.93	84.53	45.15	0.85	0.64	0.16	0.61	0.76	0.83	0.62	0.77	0.66	1.16	1.11	1.06

GY = grain yield; PH = plant height; EH = ear height; HC = husk cover; PASP = plant aspect; EPP = ears per plant; EASP = ear aspect; EROT = ear rot; MSV = maize streak virus; SCLR = southern corn leaf rust; SCLB = southern corn leaf blight; CLS = *curvularia* leaf spot; FFAWD4, FFAWD8, FFAWD12 = foliar FAW damage ratings at 4, 8, and 12 WAP; LSD(0.05) = Least Significant difference at 5% probability level.

Variance Components

Variance component analysis (Table 4) showed that the phenotypic variance exceeded the genotypic variance for all traits, with grain yield having the highest genotypic variance (44145.37) and phenotypic variance (387199.03). Grain yield had a Phenotypic Coefficient of Variation (PCV) of 14.56% and a Genotypic Coefficient of Variation (GCV) of 9.61%. Other traits, such as plant height, ear height, husk cover, plant and ear aspects, and ears per plant, often showed low genotypic variance, often close to zero. *Curvularia* leaf spot and foliar fall armyworm damage at 12 WAP resulted in GCV values of 4.74% and 4.21%, respectively. PCV values were higher than the GCV.

Table 4. Variance components for grain yield, agronomic traits, foliar disease scores, and foliar FAW damage across environments in 2024.

Trait	$\sigma^2 g$	$\sigma^2 ge$	$\sigma^2 e$	$\sigma^2 p$	GCV(%)	PCV(%)
**Grain yield (Kg/ha)**	44145.37	0.00	343053.66	387199.03	9.61	14.56
**Plant height (cm)**	0.00	0.00	5383.13	5383.13	0.00	46.25
**Ear height (cm)**	0.00	0.00	1554.13	1554.13	0.00	52.17
**Husk cover (1–5)**	0.00	0.00	0.55	0.55	0.00	24.20
**Plant aspect (1–5)**	0.00	0.03	0.31	0.34	0.00	15.39
**Ears per plant (count)**	0.00	0.00	0.02	0.02	1.91	6.77
**Ear aspect (1–5)**	0.00	0.05	0.26	0.31	0.00	15.86
**Ear rot (1–5)**	0.00	0.00	0.40	0.40	1.83	8.76
**Maize streak virus (1–5)**	0.00	0.00	0.47	0.47	0.00	42.10
**Southern corn leaf rust (1–5)**	0.00	0.02	0.30	0.32	0.00	30.92
**Southern corn leaf blight (1–5)**	0.00	0.16	0.47	0.63	0.00	29.73
***Curvularia* leaf spot (1–5)**	0.02	0.06	0.30	0.39	4.74	9.76
**Foliar FAW damage at 4 WAP (1–9)**	0.01	0.05	0.94	1.00	2.07	12.22
**Foliar FAW damage at 8 WAP (1–9)**	0.00	0.03	0.83	0.87	0.00	13.42
**Foliar FAW damage at 12 WAP (1–9)**	0.02	0.00	0.83	0.85	4.21	12.44

$\sigma^2 g$ = genotypic variance; $\sigma^2 p$ = phenotypic variance; $\sigma^2 e$ = environmental variance; GCV (%) = Genotypic Coefficient of Variation; PCV (%) = Phenotypic Coefficient of Variation.

Principal Component Analysis

Principal Component Analysis (PCA) showed the contribution of grain yield, agronomic traits, foliar diseases, and foliar FAW damage to variation among the maize hybrids and checks evaluated across environments (Table 5). The first five principal components (PC) cumulatively accounted for 69% of the total variation. The PC1 explained 22% of the variation with an eigenvalue of 3.28 and was characterised by high absolute loadings for plant aspect (PASP; 0.47), grain yield (GY; -0.43), ears per plant (EPP; -0.39), and husk cover (HC; 0.30). The PC2 explained 17% of the variation with an eigenvalue of 2.49 and was characterised by ear aspect (EASP; -0.50), ear rot (EROT; -0.46), and southern corn leaf blight (SCLB; 0.32). The PC3 accounted for 12% of the variation with an eigenvalue of 1.75 and was characterised by SCLB (-0.49), HC (0.31), *Curvularia* leaf spot (CLS; -0.33), and foliar FAW damage at 4, 8, and 12 WAP (FFAWD4, FFAWD8, and FFAWD12; -0.31, -0.32, and -0.32, respectively). The PC4 explained 11% of the variation with an eigenvalue of 1.59 and was characterised by southern corn leaf rust (SCLR; -0.52), maize streak virus (MSV; 0.49), CLS (-0.37), and plant height (PH; 0.43). The PC5 explained 9% of the total variation with an eigenvalue of 1.28 and was characterised by CLS (0.40), MSV (0.39), GY (-0.36), ear height (EH; -0.34), and FFAWD4 (-0.33).

The PCA biplot is illustrated in Fig 2. Along PC1, PASP, HC, MSV, FFAWD4, FFAWD8, and FFAWD12 were positioned on the positive side of the axis, whereas GY, EPP, PH, EH, EASP, EROT, SCLR, SCLB, and CLS were positioned on the negative side. Along PC2, PH, EH, PASP, EASP, EROT, FFAWD4, FFAWD8, and FFAWD12 were positioned on the positive side, whereas GY, HC, MSV, SCLR, SCLB, CLS, and EPP were positioned on the negative side. Genotypes G5, G7, G16, G17, and G24 were positioned on the positive side of PC1, whereas G1, G8, G9, G18, and G22 were located on the negative side. Genotypes G13,

G14, and G23 were positioned in the positive PC2 quadrant, while G3, G25, G26, and G29 were located in the negative PC2 quadrant.

Table 5. Principal component analysis of the contributions of grain yield, other agronomic traits, foliar diseases, and foliar FAW damage to total variation among the maize hybrids and checks evaluated across environments.

	Eigenvectors				
**Traits**	**PC1**	**PC2**	**PC3**	**PC4**	**PC5**
**Grain yield (kg ha^{-1})**	**-0.43**	0.03	-0.02	0.04	**-0.36**
**Plant height (cm)**	-0.05	0.17	**-0.38**	**0.43**	0.00
**Ear height (cm)**	0.12	0.20	0.22	-0.22	**-0.34**
**Husk cover (1–5)**	**0.30**	0.19	**0.31**	-0.09	-0.10
**Plant aspect (1–5)**	**0.47**	0.06	0.05	-0.11	-0.06
**Ears per plant (count)**	**-0.39**	-0.22	-0.15	-0.19	-0.22
**Ear aspect (1–5)**	0.03	**-0.50**	-0.04	-0.11	0.25
**Ear rot (1–5)**	-0.21	**-0.46**	0.07	0.01	-0.06
**Maize streak virus (1–5)**	-0.11	0.23	0.05	**0.49**	**0.39**
**Southern corn leaf rust (1–5)**	-0.15	0.23	-0.16	**-0.52**	0.06
**Southern corn leaf blight (1–5)**	0.00	**0.32**	**-0.49**	-0.03	-0.22
***Curvularia* leaf spot (1–5)**	-0.11	0.22	**-0.33**	**-0.37**	**0.40**
**Foliar FAW damage at 4 WAP (1–9)**	0.26	-0.20	**-0.31**	0.10	**-0.33**
**Foliar FAW damage at 8 WAP (1–9)**	**0.32**	-0.26	**-0.32**	-0.14	0.29
**Foliar FAW damage at 12 WAP (1–9)**	0.27	-0.14	**-0.32**	0.13	-0.27
**Eigenvalue**	3.28	2.49	1.75	1.59	1.28
**Proportion (%)**	22	17	12	11	9
**Cumulative Proportion (%)**	22	38	50	61	69

PC = principal component; bolded values (|loading| ≥ 0.30) contributed more to the variation.

Fig 2. The PCA biplot showing the relationship between PC1 and PC2 for contributions of grain yield, foliar diseases, and other agronomic traits to total variation among the maize hybrids and checks evaluated across environments. GY = grain yield; PH = plant height; EH = ear height; HC = husk cover; PASP = plant aspect; EPP = ears per plant; EASP = ear aspect; EROT = ear rot; MSV = maize streak virus; SCLR = southern corn leaf rust; SCLB = southern corn leaf blight; CLS = *curvularia* leaf spot; FFAWD4, FFAWD8, FFAWD12 =

foliar FAW damage ratings at 4, 8, and 12 WAP; G1-G32 = maize hybrids as presented in Table 1

Correlation Analysis

Several significant positive and negative Spearman's correlations were identified among the traits evaluated across environments (Fig 3). Grain yield had a strong negative correlation coefficient with plant aspect ($\rho = -0.71$, $p < 0.001$), a strong positive correlation coefficient with ears per plant ($\rho = 0.60$, $p < 0.001$), a moderate positive correlation coefficient with plant height ($\rho = 0.40$, $p < 0.05$) and ear height ($\rho = 0.38$, $p < 0.05$), and a moderate negative correlation coefficient with husk cover ($\rho = -0.38$, $p < 0.05$) and foliar fall armyworm damage at 8 WAP ($\rho = -0.43$, $p < 0.05$). Husk cover had a moderate negative correlation coefficient with ears per plant ($\rho = -0.47$, $p < 0.01$) but a moderate positive correlation coefficient with plant aspect ($\rho = 0.38$, $p < 0.05$). Ears per plant had a moderate positive correlation coefficient with ear rot ($\rho = 0.43$, $p < 0.05$) and a moderate negative correlation coefficient with plant aspect ($\rho = -0.44$, $p < 0.05$).

Ear aspect had a moderate positive correlation coefficient with ear rot ($\rho = 0.50$, $p < 0.01$) and a moderate positive correlation coefficient with foliar fall armyworm damage at 8 WAP ($\rho = 0.45$, $p < 0.05$). Foliar fall armyworm damage at 4 WAP had moderate positive correlation coefficients with damage at 8 WAP ($\rho = 0.45$, $p < 0.05$) and 12 WAP ($\rho = 0.36$, $p < 0.05$), while foliar fall armyworm damage at 8 WAP had a moderate positive correlation coefficient with damage at 12 WAP ($\rho = 0.40$, $p < 0.05$). Plant aspect had moderate positive correlation coefficients with foliar fall armyworm damage at 8 WAP ($\rho = 0.37$, $p < 0.05$) and 12 WAP ($\rho = 0.36$, $p < 0.05$), and a moderate negative correlation coefficient with plant height ($\rho = -0.44$, $p < 0.05$). Plant height had a moderate positive correlation coefficient with ear height

(ρ = 0.38, $p < 0.05$). Maize streak virus had a moderate negative correlation coefficient with foliar fall armyworm damage at 12 WAP (ρ = -0.40, $p < 0.05$).

Fig 3. Correlogram showing the relationship between traits of FAW-resistant maize hybrids and checks evaluated across environments. The cell value denotes correlation coefficient (ρ) values; *,**,*** = Significance at 0.05, 0.01, and 0.001 probability levels respectively; ns = non-significance; GY = grain yield, PH = plant height, EH = ear height. HC = husk cover, PASP = plant aspect, EPP = ears per plant, EASP = ear aspect, EROT = ear rot, MSV = maize streak virus, SCLR = southern corn leaf rust, SCLB = southern corn leaf blight, CLS = *curvularia* leaf spot, FFAWD4 = foliar FAW damage at 4 WAP, FFAWD8 = foliar FAW damage at 8 WAP, FFAWD12 = foliar FAW damage at 12 WAP.

Genotype by yield × trait analysis and superiority index

The genotype by yield × trait (GYT) analysis combined grain yield with the traits that had a significant hybrid effect, including plant aspect, ear aspect, southern corn leaf blight, *curvularia* leaf spot, and foliar fall armyworm damage at 8 and 12 WAP to rank the hybrids. The standardized genotype by yield × trait combinations and the corresponding superiority index are presented in Table ~~4.10~~. The GYT biplot explained 87.10% of the total variation among the yield × trait combinations, with PC1 accounting for 77.76% and PC2 accounting for 9.34% (Fig. 4). For clarity, entry numbers corresponding to each hybrid were used for ease of graphical presentation. Accordingly, G1 aligned with GY/PASP, GY/EASP, and GY/FFAWD8, G22 aligned with GY/FFAWD12, and G6 aligned with GY/SCLB, while the check Oba Super 11 (G28) aligned with GY/CLS. Based on the "which won where" analysis (Fig. 5), the perpendicular lines divided the polygon into eight sectors, and the yield × trait combinations were captured in sectors with vertex entries G1 (GY/PASP, GY/EASP, and

GY/FFAWD8), G22 (GY/FFAWD12), G6 (GY/SCLB), and the check G28 (GY/CLS). The Average Tester Coordination (ATC) view (Fig. 6) identified the top five hybrids as FAWSYN-1/(TZLComp. 1 C6-W-39-1-1)-B-B (G1), FAWSYN-1/IITATZI2305 (G22), FAWSYN-2/(TZLComp. 1 C6-W-39-1-1)-B-B (G9), FAWSYN-1/TZISTR1121 (G4), and FAWSYN-3/IITATZI2305 (G19), whereas the bottom five were FAWSYN-2 (G25), FAWSYN-3/TZISTR1872 (G16), FAWSYN-1/TZISTR1305 (G7), FAWSYN-2/TZISTR1305 (G13), and FAWSYN-1/TZISTR1129 (G5). Among the checks, Oba Super 11 (G28) had the highest superiority index.

Table 6: Standardized genotype by yield × trait and superiority index of FAW-resistant maize hybrids evaluated across environments in 2024.

Hybrid	Pedigree	GY/PASP	GY/EASP	GY/SCLB	GY/CLS	GY/FFAWD8	GY/FFAWD12	Superiority Index
**G1**	FAWSYN-1/(TZLComp. 1 C6-W-39-1-1)-B-B	2.38	2.56	1.87	1.19	3.01	1.22	2.04
**G22**	FAWSYN-1/IITATZI2305	1.28	1.99	-0.47	0.98	1.18	2.01	1.16
**G9**	FAWSYN-2/(TZLComp. 1 C6-W-39-1-1)-B-B	1.66	1.55	0.97	0.84	0.28	1.01	1.05
**G4**	FAWSYN-1/TZISTR1121	1.41	0.97	0.68	0.40	0.96	1.37	0.96
**G19**	FAWSYN-3/IITATZI2305	0.76	1.09	0.78	0.39	0.80	0.43	0.71
**G18**	FAWSYN-3/IITATZI2300	0.68	0.60	0.38	0.12	0.97	1.36	0.68
**G8**	FAWSYN-1/TZISTR2042	0.95	-0.01	0.57	-0.14	0.72	1.75	0.64
**G6**	FAWSYN-1/TZISTR2024	0.17	-0.54	2.69	-0.05	-0.46	0.91	0.45
**G20**	FAWSYN-1/IITATZI2300	0.63	0.70	0.39	-0.12	0.35	0.59	0.42
**G21**	FAWSYN-2/IITATZI2300	0.24	0.32	0.12	0.20	0.38	0.63	0.32
**G15**	FAWSYN-3/TZISTR1869	-0.27	-0.10	-0.13	0.26	-0.32	-0.07	-0.11
**G10**	FAWSYN-2/TZISTR1878	0.33	-0.59	-0.62	0.00	-0.12	-0.01	-0.17
**G11**	FAWSYN-2/TZISTR1129	-0.07	0.35	-0.30	-0.10	-0.36	-0.62	-0.18
**G14**	FAWSYN-2/TZISTR2129-2	-0.41	-0.12	-0.95	-0.26	0.12	0.27	-0.23
**G26**	FAWSYN-3	-0.45	-0.90	0.78	-0.49	-0.51	-0.13	-0.28
**G23**	FAWSYN-2/IITATZI2305	-0.19	0.45	-0.95	-0.31	-0.08	-0.78	-0.31
**G12**	FAWSYN-2/TZISTR2024	-0.73	-0.67	0.51	-0.35	-0.38	-0.35	-0.33
**G3**	FAWSYN-1/TZISTR1878	-0.12	-0.23	-0.61	-0.06	-0.59	-0.85	-0.41
**G2**	FAWSYN-1/TZISTR1869	-0.51	-0.69	-0.27	-0.46	-0.33	-0.28	-0.42
**G17**	FAWSYN-3/TZISTR1878	-0.66	-0.68	-1.26	-0.53	-0.98	0.07	-0.67
**G24**	FAWSYN-1	-1.12	-0.58	-0.36	-0.88	-0.45	-1.08	-0.75
**G5**	FAWSYN-1/TZISTR1129	-0.98	-0.65	-0.42	-0.32	-0.88	-1.24	-0.75
**G13**	FAWSYN-2/TZISTR1305	-1.16	-0.53	-1.20	-0.93	-0.49	-0.84	-0.86
**G7**	FAWSYN-1/TZISTR1305	-1.53	-0.98	-1.36	-1.03	-0.47	-0.86	-1.04
**G16**	FAWSYN-3/TZISTR1872	-1.33	-1.09	-0.75	-1.17	-1.42	-1.27	-1.17
**G25**	FAWSYN-2	-1.36	-1.80	-0.99	-1.16	-1.23	-1.91	-1.41
**Checks**								
**G28**	Oba Super 11	1.93	1.51	2.31	4.45	2.72	1.51	2.41
**G31**	Oba Super 9	0.41	0.52	0.00	0.37	0.13	-0.38	0.18
**G27**	SAMMAZ 51	-0.13	-0.15	-0.44	-0.34	-0.65	-0.01	-0.29
**G30**	Oba Super 7	-0.34	-0.07	-0.44	-0.21	0.12	-0.89	-0.31
**G32**	Oba Super II	-0.93	-0.77	0.51	0.46	-0.88	-0.69	-0.38
**G29**	SC301	-0.55	-1.46	-1.04	-0.73	-1.16	-0.88	-0.97
**Mean**		0.00	0.00	0.00	0.00	0.00	0.00	
**Standard deviation**		1.00	1.00	1.00	1.00	1.00	1.00	

GY = grain yield, DP = days to pollination, DS = days to silking, ASI = anthesis-silking interval, EH = ear height, HC = husk cover, PASP = plant aspect, SCLB = southern corn leaf blight, CLS = *curvularia* leaf spot, FFAWD8, FFAWD12 = foliar FAW damage at 8 and 12 WAP.

Fig 4. GYT biplot showing the relationships among the yield-trait combination of the 32 FAW-resistant maize hybrids and checks evaluated across environments in 2024. GY = grain yield, PASP = plant aspect, EASP = ear aspect, SCLB = southern corn leaf blight, CLS = *curvularia* leaf spot, FFAWD8, FFAWD12 = foliar FAW damage at 8 and 12 WAP, G1-G32 = maize hybrids as presented in Table 1.

Fig 5. Which-won-where view of the GYT biplot for the 32 FAW-resistant maize hybrids and checks evaluated across environments in 2024. GY = grain yield, PASP = plant aspect, EASP = ear aspect, SCLB = southern corn leaf blight, CLS = *curvularia* leaf spot, FFAWD8, FFAWD12 = foliar FAW damage at 8 and 12 WAP, G1-G32 = maize hybrids as presented in Table 1.

Fig 6. The Average Tester Coordination view of the genotype by yield × trait (GYT) biplot for the 32 FAW-resistant maize hybrids and checks evaluated across environments in 2024. GY = grain yield, PASP = plant aspect, EASP = ear aspect, SCLB = southern corn leaf blight, CLS = *curvularia* leaf spot, FFAWD8, FFAWD12 = foliar FAW damage at 8 and 12 WAP, G1-G32 = maize hybrids as presented in Table 1.

Discussion

Insufficient maize production in many regions is largely driven by FAW outbreaks, which could reduce maize yields and cause annual [illegible] that jeopardize the livelihoods of smallholder farmers (11). Cultivating resistant hybrids could reduce insecticide dependence, lower production costs, and contribute to sustainable food security in FAW-affected regions (22). Thirty-two FAW-resistant maize hybrids and checks were evaluated across two environments exposed to natural FAW pressure to determine resistance levels and yield

potential. Rainfall was highly irregular, and FAW pressure varied but remained sufficient at both experimental sites, allowing differentiation of hybrid responses.

The significant hybrid effect, along with hybrid × environment (G × E) and environment effects for several traits, indicated genetic variation whose expression depended on growing conditions. Similar patterns were reported in FAW-resistance studies in which genotype and G × E effects were significant for grain yield and FAW leaf and ear damage under artificial or natural infestation (33–35). The significant environmental effects observed further indicated that environmental conditions strongly influenced both productivity and resistance expression (22,33,34). The high R^2 values were consistent with reports of moderate-to-high heritability for FAW foliar damage and grain yield, which suggested that selection for resistance and agronomic performance could be effective when trials were appropriately replicated across environment (22,36). The coefficients of variation above 20% for grain yield and foliar FAW damage were consistent with FAW screening trials and likely reflected heterogeneous pest pressure and complex FAW damage–yield relationships (33–35).

Grain yield levels were lower than those reported in FAW-tolerant hybrid trials conducted under on-station, artificial infestation, or well-managed conditions, where tolerant hybrids frequently produced 4.6–7.1 t/ha, and some entries exceeded 7 t/ha under FAW pressure (22,36,37). However, comparable or lower yields were reported under natural infestation and smallholder-type stress conditions, such as in Mozambique, where the mean yield was approximately 0.8 t/ha, and the best entry reached about 1.3 t/ha (38). This contrast suggested that yield performance was strongly influenced by the intensity of environmental stress, particularly irregular rainfall, agronomic management, and pest pressure (22,36). Foliar FAW damage levels were consistent with ranges reported in hybrid and open-pollinated maize hybrids evaluated under artificial infestation, where damage scores commonly ranged from moderate to moderately high depending on genotype and infestation timing (33,35,37). Similar

studies reported that hybrids with lower foliar FAW damage tended to maintain higher grain yield, reflecting the negative relationship between foliar FAW damage and productivity (33,39). Ear aspect, husk cover, and ear rot responses aligned with reports describing FAW-tolerant hybrids as possessing acceptable ear quality, improved husk protection, and reduced secondary ear damage, thereby helping maintain yield under infestation (22,37,39). Comparable performance of commercial checks relative to trial entries was also reported in FAW screening studies, in which checks frequently fell within the overall range. Still, it was often surpassed by improved FAW-resistant hybrids (22,34,35).

The negligible or zero genotypic variance for most agronomic and disease traits and for several foliar FAW damage scores indicated that environmental factors played a dominant role in trait expression (40). This could be due to spatially heterogeneous factors or environmental variability, such as soil fertility or moisture, which may also have been absorbed into the residual term, thereby lowering the estimated genotypic variance. This is in contrast with other multi-environment studies that reported non-zero genotypic variance and moderate genotypic coefficients of variation for grain yield and foliar FAW damage traits (26,33,34). In contrast, some FAW studies that applied mixed-model approaches, including spatial or related adjustments, often reduced residual variance and recovered larger genotypic variance components (29,41). Genotype composition may also have contributed; closely related elite maize hybrids could have expressed genuinely smaller among-genotype variance for several traits than more diverse sets that included broad germplasm sources, landraces, or open-pollinated varieties (33,34).

The first two principal components were primarily associated with agronomic and ear-quality traits, whereas foliar FAW damage scores contributed more strongly to later components. This differed from other FAW studies, in which early principal components were dominated by foliar FAW-damage variables and their association with grain yield (36,42). This

could be due to the inclusion of broader agronomic traits, consistent with studies that predominantly focus on plant architecture and yield-related variables, such as plant height, ear traits, and grain yield (43–45). This pattern suggested that variation among maize hybrids was more strongly explained by plant morphology, yield components, and disease-related traits than by foliar FAW damage scores alone.

The Spearman's correlation pattern indicated that grain yield was closely associated with plant architecture and key yield components, consistent with FAW-infested multi-environment trials in which ears per plant, plant height, ear height, and ear-related traits were major contributors to yield variation under stress conditions (34,36,38). Negative associations between foliar FAW damage and grain yield were also consistent with findings from Mozambique, Nepal, and other FAW-affected environments, where foliar and ear damage reduced grain yield and ear development. However, the magnitude of these relationships depended on genotype and infestation intensity (46,47). Similar relationships were reported under both controlled infestation and field conditions, indicating that foliar FAW damage reduced productivity primarily through its effects on plant vigor and ear appeal (22,48,49). Consistent associations among foliar FAW damage scores across the growth stages and with agronomic traits were also reported, reflecting the tendency for susceptible maize hybrids to exhibit sustained injury and reduced agronomic performance. In contrast, tolerant hybrids maintained more favorable growth and yield characteristics under infestation (34,36).

Grain yield is a genetically complex trait influenced by multiple agronomic and environmental factors; therefore, direct selection for yield alone is often inefficient, particularly under variable stress conditions (50). In contrast, phenology, growth, and resistance-related traits often exhibited higher heritability and lower genotype × environment sensitivity, making them more reliable targets for indirect selection (51). Multi-trait indices such as GYT therefore provided a more robust basis for advancement because they ranked genotypes using yield–trait

combinations that jointly captured productivity, stress tolerance, and overall plant type (31,52). The high ranking of the commercial check, OBA Super 11, on the GYT superiority index indicated a favorable aggregate profile of grain yield, phenotypic appeal, foliar diseases, and foliar FAW damage, consistent with reports in which commercial checks or released cultivars occupied leading GYT positions because of broadly optimized trait packages (53–55). Advancing the ~~highest-ranked~~ experimental hybrids based on the GYT superiority index, therefore prioritized entries that combined yield with other traits with significant hybrid effects, supporting more efficient parent selection and cultivar advancement than selection based on grain yield alone (56,57).

Conclusions

Unpredictable rainfall patterns, including cessation of rainfall after planting and excessive rainfall after maize reached maturity, likely imposed moisture stress during early growth stages and increased lodging, thereby affecting yield evaluation. Despite these constraints, genetic variability was observed among the fall armyworm-resistant maize hybrids evaluated, and environmental conditions significantly influenced phenotypic expression, potentially affecting trait expression and hybrid rankings. A subset of hybrids nonetheless maintained comparatively higher grain yield across environments, indicating potential for selection under FAW pressure. Grain yield reductions associated with foliar FAW damage and unfavorable plant traits likely translated into economic losses for farmers. Cultivation of the identified fall armyworm-resistant maize hybrids could therefore improve profitability and support more sustainable production in the derived savanna agro-ecology. The top-performing topcross hybrids identified showed potential to reduce the impact of FAW damage; however, additional multi-environment testing and validation were recommended to confirm

performance across a wider range of environments and management conditions and to support release to smallholder farmers through location-specific hybrid targeting.

Acknowledgement

We thank the International Institute of Tropical Agriculture (IITA), Ibadan, Nigeria, for providing the fall armyworm-resistant maize hybrids used in this study.

References

1. Erenstein O, Jaleta M, Sonder K, Mottaleb K, Prasanna BM. Global maize production, consumption and trade: trends and R&D implications. Food Secur. 2022 Oct;14(5):1295–319. doi:10.1007/s12571-022-01288-7

2. Olayinka AO. Field Performance of Extra-Early Maturing Pro-Vitamin A Maize (Zea mays L.) Hybrids under Optimal Rainfed Conditions in the Derived Savanna Agroecology Zone. Crop Breed Genet Genomics. 2025 Aug;7(3). doi:10.20900/cbgg20250012

3. Yang N, Yan J. New genomic approaches for enhancing maize genetic improvement. Curr Opin Plant Biol. 2021 Apr;60:101977. doi:10.1016/j.pbi.2020.11.002

4. Klein HS, Luna FV. The Impact of the Rise of Modern Maize Production in Brazil and Argentina. Hist Agrar Rev Agric E Hist Rural. 2022 Apr 1. doi:10.26882/histagrar.086e09k

5. Badu-Apraku B, Fakorede MAB. Maize in Sub-Saharan Africa: Importance and Production Constraints. In: Advances in Genetic Enhancement of Early and Extra-Early Maize for Sub-Saharan Africa [Internet]. Cham: Springer International Publishing; 2017 [cited 2025 Jul 30]. p. 3–10. Available from: http://link.springer.com/10.1007/978-3-319-64852-1_1 doi:10.1007/978-3-319-64852-1_1

6. Sasu. Nigeria: Maize production quantity| Statista [Internet]. 2024. Available from: https://www.statista.com/statistics/1300743/production-volume-of-maize-in-nigeria/

7. Iseghohi I, Abe A, Meseka S, Mengesha W, Gedil M, Job A, et al. Reactions of provitamin-A-enriched maize to foliar diseases under field conditions in Nigeria. Cereal Res Commun. 2024 Jun 1;52(2):747–58. doi:10.1007/s42976-023-00395-1

8. Goergen G, Kumar PL, Sankung SB, Togola A, Tamò M. First Report of Outbreaks of the Fall Armyworm Spodoptera frugiperda (J E Smith) (Lepidoptera, Noctuidae), a New Alien

Invasive Pest in West and Central Africa. Luthe DS, editor. PLOS ONE. 2016 Oct 27;11(10):e0165632. doi:10.1371/journal.pone.0165632

9. Iseghohi I, Ogundare E, Fayemiro S, Obisesan O, Job A. Assessment of maize hybrids for agronomic and fall armyworm tolerant traits. J Cent Eur Agric. 2025;26(4):927–38. doi:10.5513/JCEA01/26.4.4631

10. Day R, Abrahams P, Bateman M, Beale T, Clottey V, Cock M, et al. Fall Armyworm: Impacts and Implications for Africa. Outlooks Pest Manag. 2017 Oct 1;28(5):196–201. doi:10.1564/v28_oct_02

11. Togola A, Beyene Y, Bocco R, Tepa-Yotto G, Gowda M, Too A, et al. Fall armyworm (Spodoptera frugiperda) in Africa: Insights into biology, ecology and impact on staple crops, food systems and management approaches. Frontiers in Agronomy [Internet]. Frontiers Media SA; 2025 [cited 2026 Feb 9]. p. 1538198. Available from: https://www.frontiersin.org/journals/agronomy/articles/10.3389/fagro.2025.1538198/full

12. Abro Z, Kimathi E, De Groote H, Tefera T, Sevgan S, Niassy S, et al. Socioeconomic and health impacts of fall armyworm in Ethiopia. Nagoshi RN, editor. PLOS ONE. 2021 Nov 4;16(11):e0257736. doi:10.1371/journal.pone.0257736

13. Utono IM, Adamu RS. Effect of belt expert (Flubendiamide + Thiacloprid), Imidacloprid, Thiamethoxam seed treatment and economic impact on fall armyworm (*Spodoptera frugiperda*) infestation on Maize in Nigeria. Cogent Food Agric. 2023 Dec 31;9(1):2164117. doi:10.1080/23311932.2022.2164117

14. Tambo JA, Kansiime MK, Mugambi I, Agboyi LK, Beseh PK, Day R. Economic impacts and management of fall armyworm (Spodoptera frugiperda) in smallholder agriculture: a panel data analysis for Ghana. CABI Agric Biosci. 2023 Oct 2;4(1):38. doi:10.1186/s43170-023-00181-3

15. Tambo JA, Liverpool-Tasie LSO. Are farm input subsidies a disincentive for integrated pest management adoption? Evidence from Zambia. J Agric Econ. 2024 Jun;75(2):740–63. doi:10.1111/1477-9552.12582

16. Kenyi JJ, Mengesha W, Abe A, Menkir A, Meseka S. Variation in grain yield losses due to fall armyworm infestation among elite open-pollinated maize varieties under different levels of insecticide application. Agriculture. 2024;14(7):984. doi:10.3390/agriculture14070984

17. Matova PM, Kamutando CN, Warburton ML, Williams WP, Magorokosho C, Shimelis H, et al. New techniques for breeding maize (*ZEA MAYS*) varieties with fall armyworm resistance and market-preferred traits for sub-Saharan Africa. Plant Breed. 2023 Feb;142(1):1–11. doi:10.1111/pbr.13063

18. Woolfolk SW, Jeffers D, Hawkins LK, Uhdre R, Ni X, Warburton ML. Integrated approaches to maximizing maize resistance to fall armyworm. CABI Rev. 2025 Feb 20;0013. doi:10.1079/cabireviews.2025.0013

19. Krishna VV, Lantican MA, Prasanna BM, Pixley K, Abdoulaye T, Menkir A, et al. Impact of CGIAR maize germplasm in Sub-Saharan Africa. Field Crops Res. 2023 Jan;290:108756. doi:10.1016/j.fcr.2022.108756

20. Adunola MP, Fayeun LS, Fadara AB. Impact of climate change on armyworm infestation on maize in Nigeria: A review. J Plant Breed Crop Sci. 2021 Sep 30;13(3):158–67. doi:10.5897/JPBCS2020.0895

21. Asare S, Kena A, Amoah S, Annor B, Osekre EA, Akromah R. Screening of maize inbred lines and evaluation of hybrids for their resistance to fall armyworm. Plant Stress. 2023;8:100148. doi:10.2139/ssrn.4206925

22. Amegbor IK, Adu GB, Nelimor C, Prasanna BM, Beyene Y, Chivasa W, et al. Assessment of fall armyworm tolerant maize hybrids for sustainable maize production in sub-Saharan Africa. Phytoparasitica. 2025 Feb;53(2):29. doi:10.1007/s12600-025-01253-y PubMed PMID: 39974150.

23. Ni X, Huffaker A, Schmelz EA, Xu W, Williams WP, Guo B, et al. Field evaluation of experimental maize hybrids for resistance to the fall armyworm (Lepidoptera: Noctuidae) in a warm temperate climate. Insects. 2024;15(4):289. doi:10.3390/insects15040289

24. Kolawole AO, Olayinka AF. Phenotypic performance of new pro-vitamin A maize (Zea mays L.) hybrids using three selection indices. Agric Pol Nohospodárstvo. 2022 Apr;68(1):1–12. doi:10.2478/agri-2022-0001

25. Badu-Apraku B, Fakorede MAB, Menkir A, Sanogo D. Conduct and management of maize field trials [Internet]. 2012 [cited 2025 Jul 30]. Available from: https://cgspace.cgiar.org/bitstreams/81fda619-5d11-4b6f-9885-bf75ba268cc7/download

26. Matova PM, Kamutando CN, Magorokosho C, Kutywayo D, Gutsa F, Labuschagne M. Fall-armyworm invasion, control practices and resistance breeding in Sub-Saharan Africa. Crop Sci. 2020 Nov;60(6):2951–70. doi:10.1002/csc2.20317

27. Olivoto T, Lúcio AD. metan: An R package for multi-environment trial analysis. Jarman S, editor. Methods Ecol Evol. 2020 Jun;11(6):783–9. doi:10.1111/2041-210X.13384

28. Abebe AT, Adewumi AS, Adebayo MA, Shaahu A, Mushoriwa H, Alabi T, et al. Genotype x environment interaction and yield stability of soybean (Glycine max l.) genotypes in multi-environment trials (METs) in Nigeria. Heliyon. 2024 Oct;10(19):e38097. doi:10.1016/j.heliyon.2024.e38097

29. Alvarado G, Rodríguez FM, Pacheco A, Burgueño J, Crossa J, Vargas M, et al. META-R: A software to analyze data from multi-environment plant breeding trials. Crop J. 2020;8(5):745–56. doi:10.1016/j.cj.2020.03.010

30. Jolliffe IT, Cadima J. Principal component analysis: a review and recent developments. Philos Trans R Soc Math Phys Eng Sci. 2016 Apr 13;374(2065):20150202. doi:10.1098/rsta.2015.0202

31. Yan W, Frégeau-Reid J. Genotype by yield* trait (GYT) biplot: a novel approach for genotype selection based on multiple traits. Sci Rep. 2018;8(1):8242.

32. Gholizadeh A, Ghaffari M. Genotype by yield* trait (GYT) biplot analysis: A novel approach for phenotyping sunflower single cross hybrids based on multiple traits. Food Sci Nutr. 2023 Oct;11(10):5928–37. doi:10.1002/fsn3.3524

33. Abubakar AM, Adejumobi II, Oyekunle M, Bonkoungou TO, Muhammad U, Udah O, et al. Screening for Fall Armyworm (*SPODOPTERA FRUGIPERDA* J. E. Smith) Resistance in Early-Maturing Tropical Maize Adapted to Sub-Saharan Africa. Plant Breed. 2025 Jun 4;pbr.13291. doi:10.1111/pbr.13291

34. Kamweru I, Beyene Y, Bruce AY, Makumbi D, Adetimirin VO, Pérez-Rodríguez P, et al. Genetic analyses of tropical maize lines under artificial infestation of fall armyworm and foliar diseases under optimum conditions. Front Plant Sci. 2023 Jan;14:1086757. doi:10.3389/fpls.2023.1086757 PubMed PMID: 36743507.

35. Job A, Iseghohi I, Abe A, Yahaya M, Olayiwola R, Akinwale R, et al. Genetic analysis of agronomic and fall armyworm-resistance traits in maize hybrids with varying levels of resistance to stem borers. Agronomy. 2022;12(12):3042. doi:10.3390/agronomy12123042

36. Wambi W, Makumbi D, Asea G, Zeleke H, Bruce A, Wakgari M, et al. Use of multi-trait principal component selection index to identify fall armyworm (Spodoptera frugiperda) resistant maize genotypes. Front Plant Sci. 2025 Mar 27;16. doi:10.3389/fpls.2025.1544010

37. Nesma AZM, Ayodeji A, Anthony OJ, Yinka OK, Amudalat BO. Evaluation of stem borer resistant maize genotypes for resistance to fall armyworm (Spodoptera frugiperda J.E. SMITH) infestation. J Plant Breed Crop Sci. 2023 Sep 30. doi:10.5897/jpbcs2023.1023

38. Mazive GA, Mubai NH, Magamba CP, Afo DIB, Boa AF. Grain yield and its relationship with yield components in selected maize (Zea mays L.) varieties under natural fall armyworm infestation in Mozambique. Contrib Las Cienc Soc. 2024 Dec;17(13). doi:10.55905/revconv.17n.13-228

39. Kasoma C, Shimelis H, Laing M, Shayanowako A, Mathew I. Screening of inbred lines of tropical maize for resistance to fall armyworm, and for yield and yield-related traits. Crop Prot. 2020 Oct 1;136:105218. doi:10.1016/j.cropro.2020.105218

40. Alam M, Rahman M, Ahmed S, Jahan N, Khan M, Islam M, et al. Genetic Variation and Genotype by Environment Interaction for Agronomic Traits in Maize (Zea mays L.) Hybrids. Plants. 2022 Jun 6;11(11):1522. doi:10.3390/plants11111522

41. Argaw T, Fenta B, Zegeye H, Azmach G, Funga A. Multi-environment trials data analysis: linear mixed model-based approaches using spatial and factor analytic models. Front Res Metr Anal. 2025 Apr 11;10. doi:10.3389/frma.2025.1472282

42. Stelinski L, Gre N, Ni X, Huffaker A, Schmelz E, Xu W, et al. Field Evaluation of Experimental Maize Hybrids for Resistance to the Fall Armyworm (Lepidoptera: Noctuidae) in a Warm Temperate Climate. Insects. 2024 Apr 1;15. doi:10.3390/insects15040289

43. Talefe W. Cluster and principal component analysis of maize inbred lines in low moisture stress areas in Ethiopia. Int J Agric Sci Food Technol. 2023 Jul 6. doi:10.17352/2455-815x.000194

44. Carnimeo ESG, Bertasello LET, Dutra SMF, Môro GV. Principal component analysis for selection of superior maize genotypes. 55292020V48N4P357-362. 2020. doi:10.15361/1984-

45. Singh D, Swapnil, Kushwaha N, Mohanty TA, Kumar A, Kumar R, et al. Multivariate analysis for yield and its component traits in Quality Protein Maize (QPM). Int J Adv Biochem Res. 2024 Jan 1. doi:10.33545/26174693.2024.v8.i2i.807

46. Bhandari GS, Bhandari P, Sah L. Evaluation of maize genotypes for resistance to fall armyworm under natural infestation conditions. Nepal Agric Res J. 2025 Jun 25. doi:10.3126/narj.v16i1.80465

47. Gadekar M, Das S, Shukla A, Sharma S, Patnaik S, Kakade S. Quantification of Yield Losses in Maize Attributed to Fall Armyworm (Spodoptera frugiperda) Infestation. J Sci Res Rep. 2025 Sep 18. doi:10.9734/jsrr/2025/v31i93516

48. Bakry M, Abdel-Baky N. Impact of the fall armyworm, Spodoptera frugiperda (Lepidoptera: Noctuidae) infestation on maize growth characteristics and yield loss. Braz J Biol Rev Brasleira Biol. 2023 Jul 21;84. doi:10.1590/1519-6984.274602

49. Anjorin F, Odeyemi OO, Adenike O, Kehinde A, Kareem T. Fall armyworm (Spodoptera frugiperda) (J. E. Smith) (Lepidoptera: Noctuidae) infestation: maize yield depression and physiological basis of tolerance. J Plant Prot Res. 2023 Mar 22. doi:10.24425/jppr.2022.140294

50. Fadhli N, Farid Muh, Azrai M, Nur A, Efendi R, Priyanto S, et al. Morphological parameters, heritability, yield component correlation, and multivariate analysis to determine secondary characters in selecting hybrid maize. Biodiversitas J Biol Divers. 2023 Jul 18. doi:10.13057/biodiv/d240712

51. Singamsetti A, Shahi J, Zaidi P, Seetharam K. Study on Applicability of Genotype × Yield × Trait (GYT) Biplots over Genotype × Trait (GT) Biplots in Selection of Maize Hybrids across Soil Moisture Regimes. Indian J Agric Res. 2022 Mar 4. doi:10.18805/ijare.a-5850

52. Hosseini SMS, Shiri M, Mostafavi K, Mohammadi A, Miri SM. Genetic analysis and association detection of agronomic traits in maize genotypes. Sci Rep. 2025 Jan 2;15. doi:10.1038/s41598-024-84471-4

53. Bakhshi B, Amiri Oghan H, Rameeh V, Zeinalzadeh Tabrizi H, Askari A, Faraji A, et al. Trait profiling and genotype selection in oilseed rape using genotype by trait and genotype by yield*trait approaches. Food Sci Nutr. 2023 Feb 28;11:3083–95. doi:10.1002/fsn3.3290

54. Shojaei SH, Mostafavi K, Khosroshahli M, Bihamta MR, Ramshini H. Assessment of genotype-trait interaction in maize (Zea mays L.) hybrids using GGT biplot analysis. Food Sci Nutr. 2020 Aug;8(10):5340–51. doi:10.1002/fsn3.1826 PubMed PMID: 33133537.

55. Yan W. Two types of biplots to integrate multi-trial and multi-trait information for genotype selection. Crop Sci. 2024 Mar 20. doi:10.1002/csc2.21231

56. Dang X, Lin Y, Hu X, Yanli L. Comprehensive evaluation of sugar beet varieties based on genotype × yield × trait (GYT) in different environments. Sci Rep. 2025 Mar 21;15. doi:10.1038/s41598-025-90126-9

57. Rad MRN, Bakhshi B, Ghasemi A, Ghasemi MM, Kohpalekani JA. Identification of High-Yielding and Drought-Tolerant Melon Genotypes Based on Yield–Trait Combinations: A

Comparison of Pcor Index and GYT Biplot Approaches. Food Sci Nutr. 2025 Feb 28;13. doi:10.1002/fsn3.70048

Fig 1 Click here to access/download;Figure;Fig 1.tif

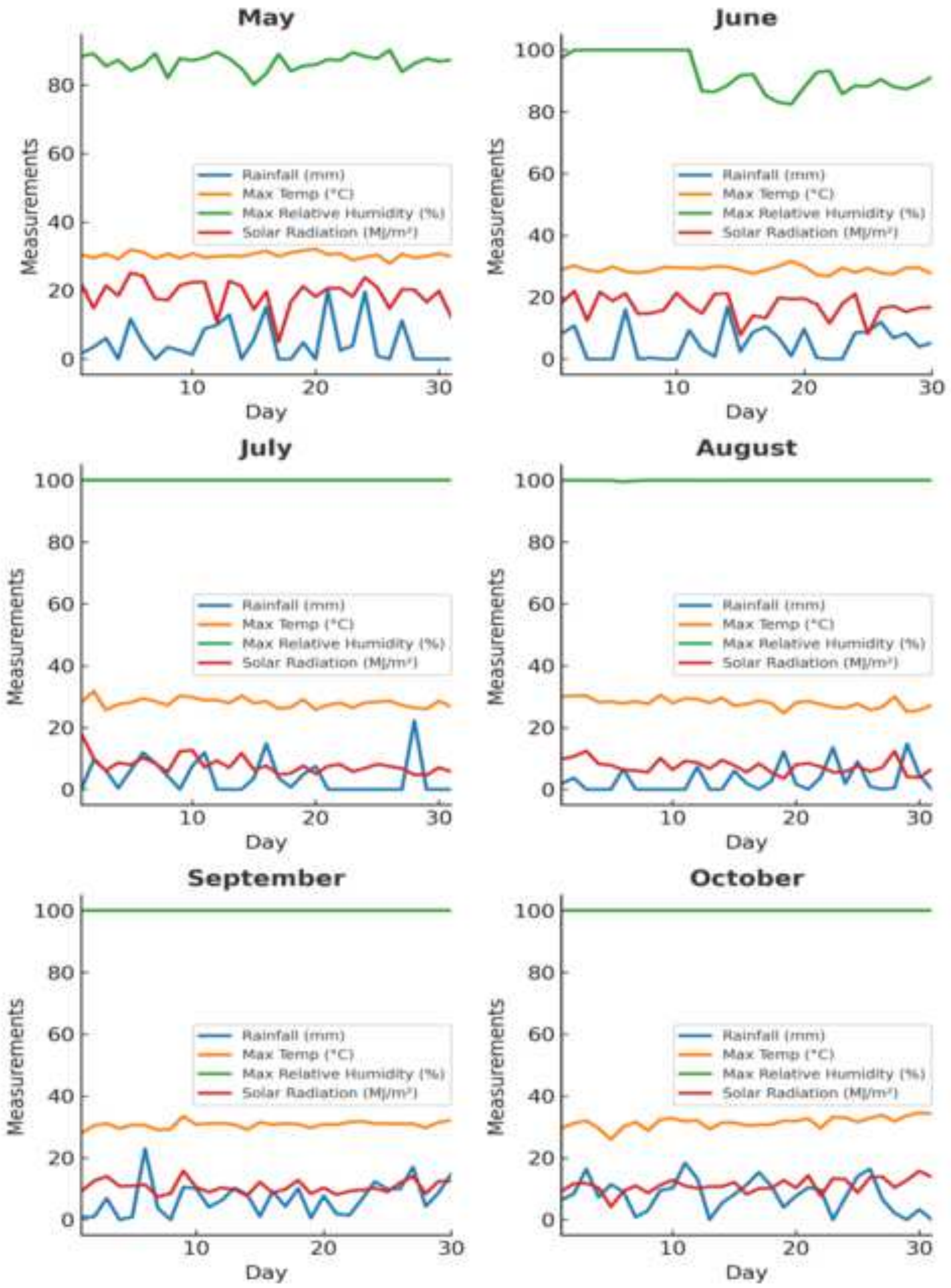

Fig 2

Click here to access/download;Figure;Fig 2.tif

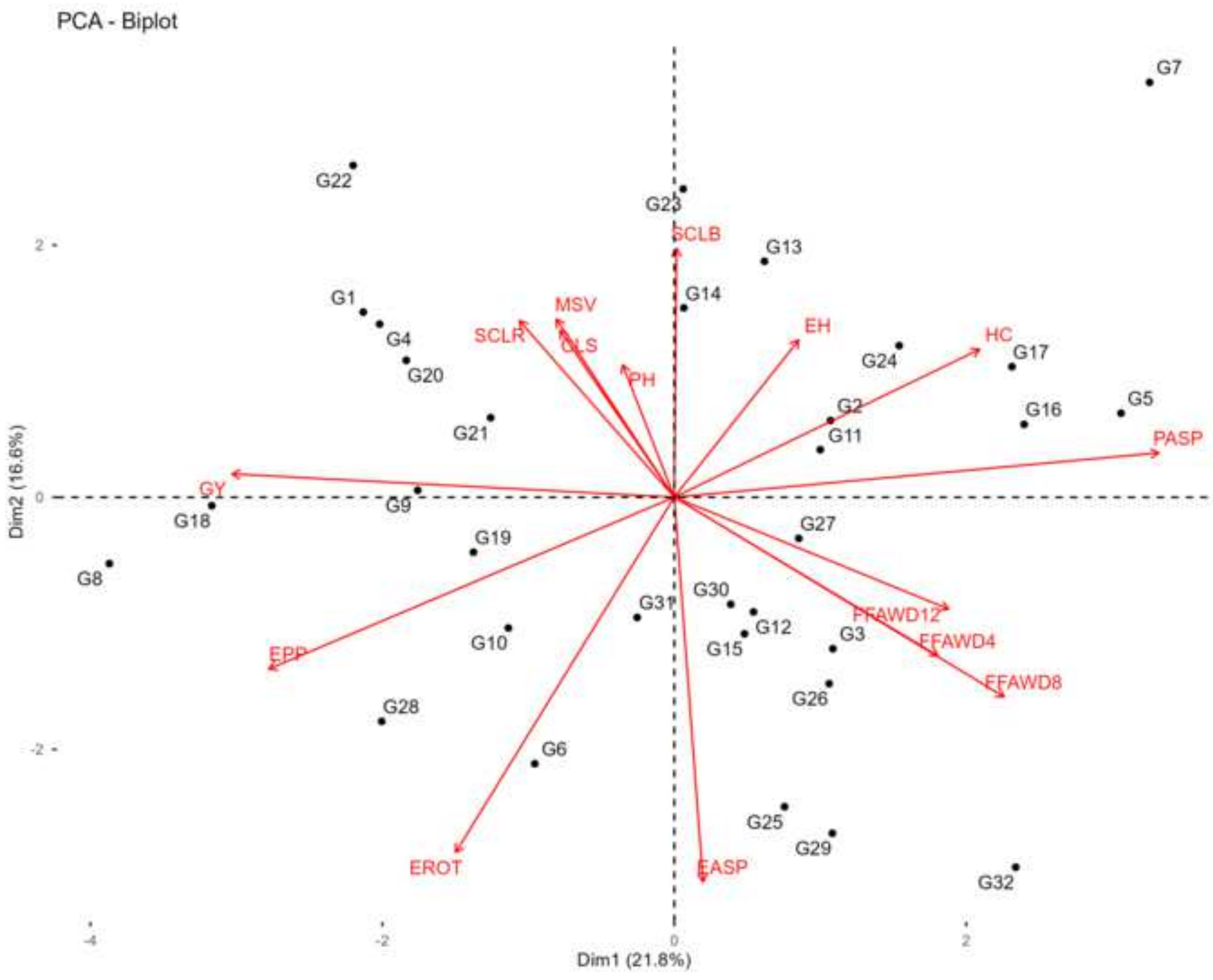

Fig 3

Click here to access/download;Figure;Fig 3.tif

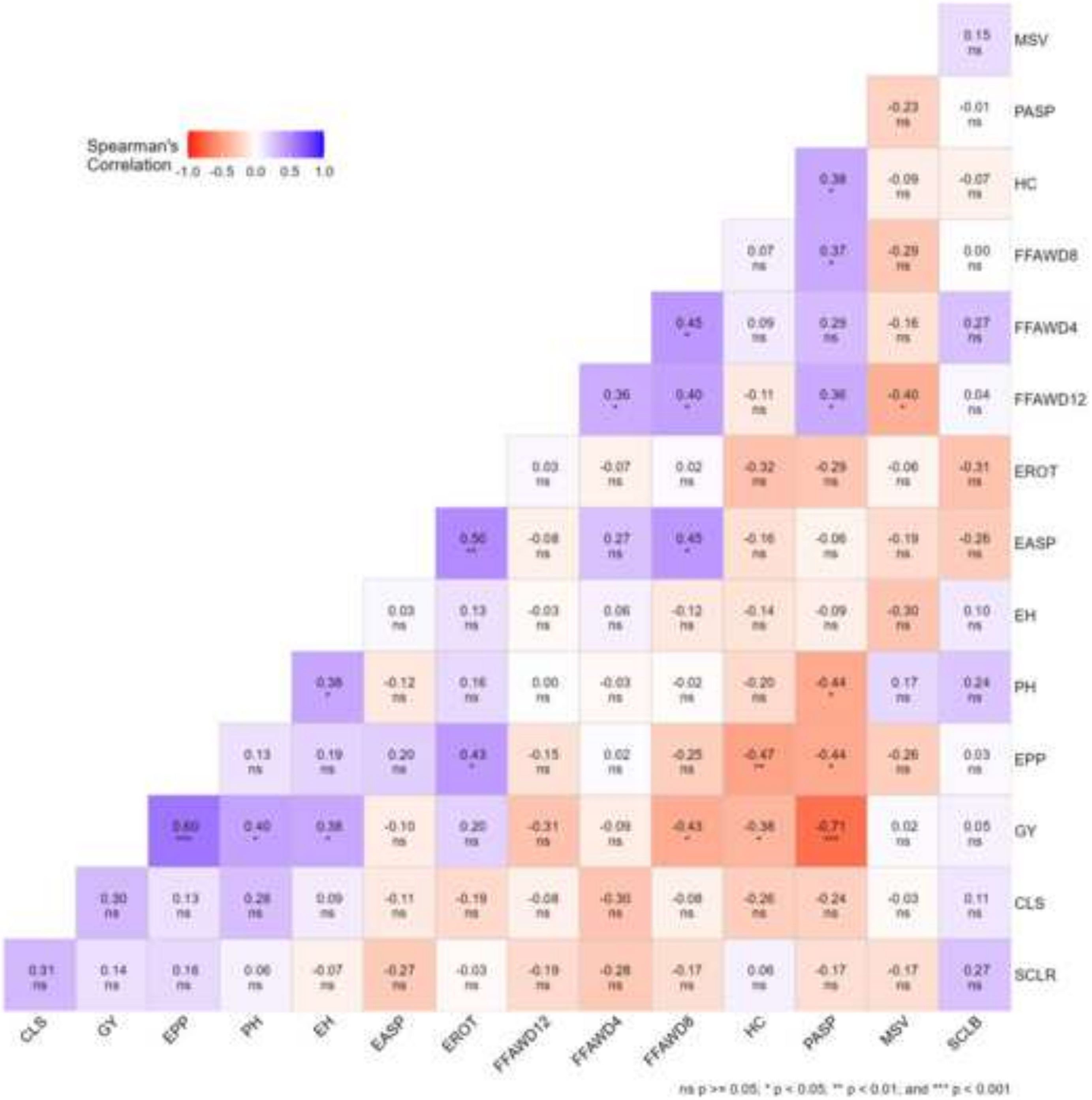

Fig 4

Click here to access/download;Figure;Fig 4.tif

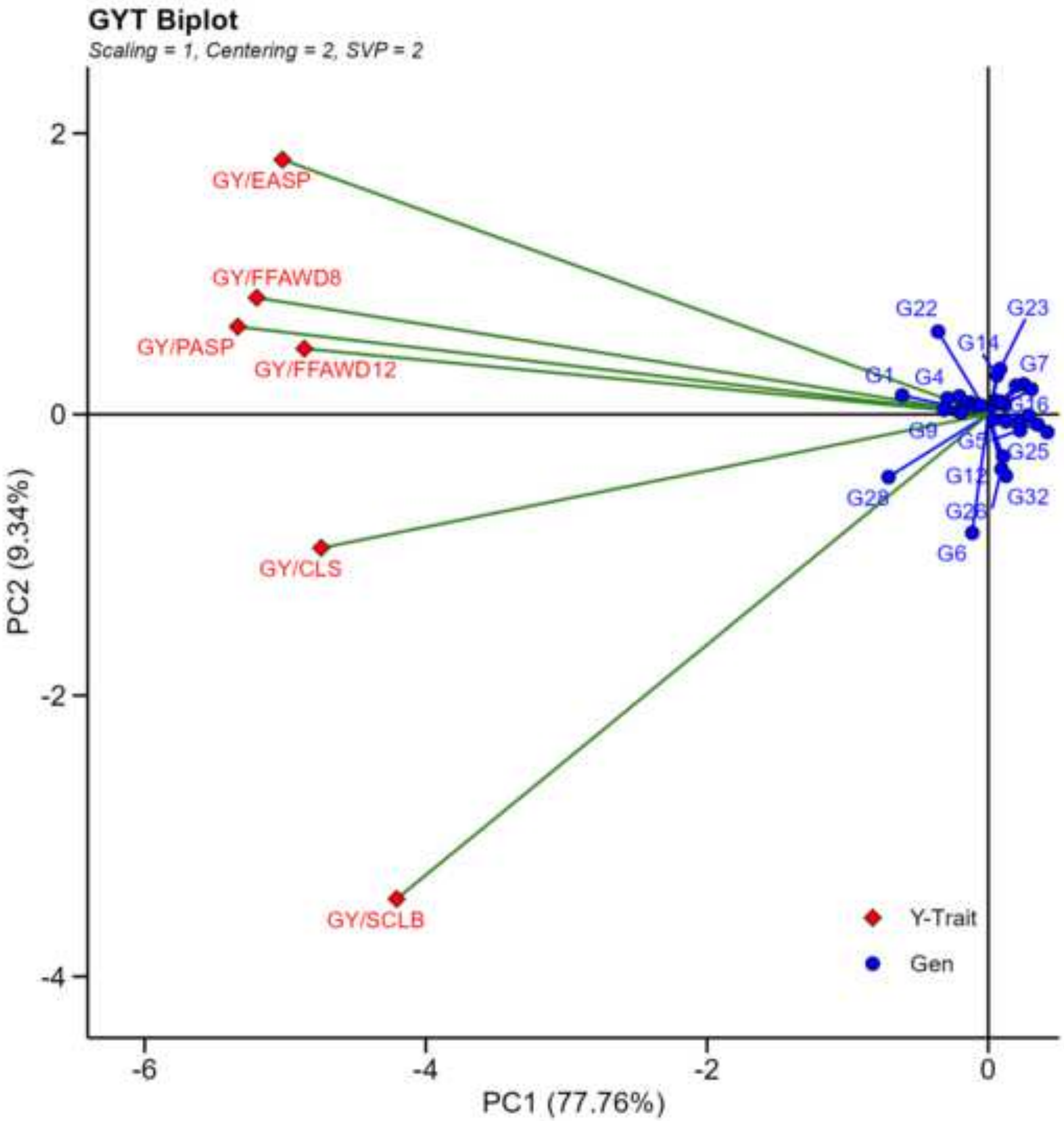

 Click here to access/download;Figure;Fig 5.tif

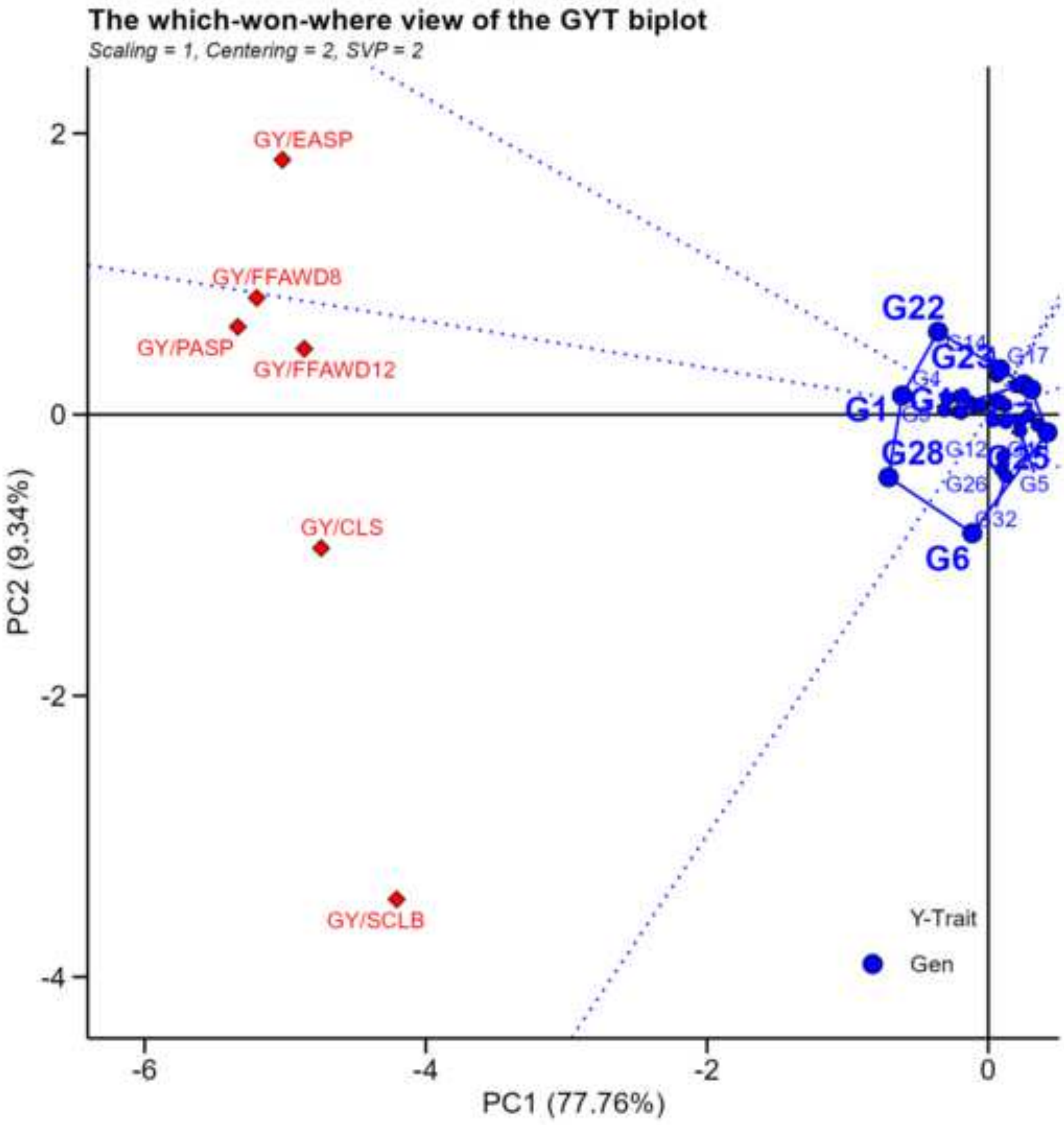

Fig 6

Click here to access/download;Figure;Fig 6.tif

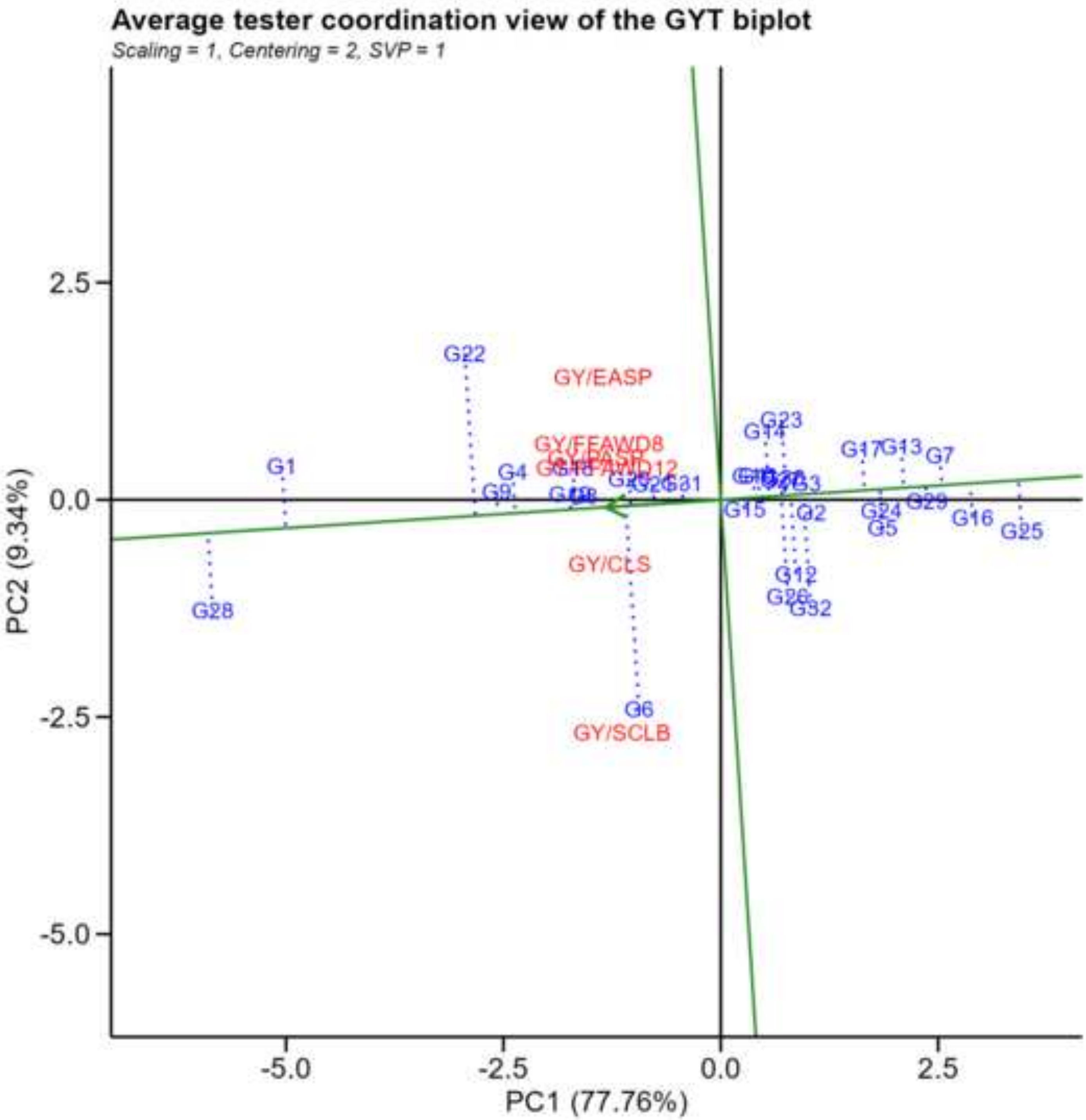

Style Definition: Caption: Font: Bold, English (United States), Line spacing: Double

Assessment of agronomic traits in fall armyworm (*Spodoptera frugiperda*) resistant maize (*Zea mays*) hybrids grown in the derived savanna agro-ecology of Nigeria

Formatted: Font: 14 pt

Formatted: Left, Line spacing: single

Formatted: Font: 14 pt

Akande, O.S.[1], Olayinka, A. O.[1*], Adebayo, M.A.[2]

[1]Crop Production and Soil Science Department, Ladoke Akintola University of Technology, PMB 4000, Ogbomoso, Oyo State, Nigeria.

Formatted: Space After: 12 pt, Line spacing: Multiple 1.15 li

[2]Department of Crop and Animal Science, Ajayi Crowther University, P.M.B. 1066, Oyo, Oyo State, Nigeria.

[*]Corresponding author

Email: aokolawole@lautech.edu.ng (AO)

Abstract

Maize production in Nigeria has increased ~~since 2010~~in recent years, but fall armyworm infestation remains a major constraint~~. To address this, 26~~ to sustainable high productivity, particularly during the minor cropping season. Twenty-six fall armyworm-resistant maize hybrids and six commercial checks were ~~obtained from the International Institute of Tropical Agriculture (IITA), Ibadan. Their agronomic performance was~~ evaluated for grain yield and other traits using an 8 × 4 ~~α~~alpha-lattice design with three replications~~ at teaching and research farms of Ladoke Akintola University of Technology, Ogbomoso, and Ajayi Crowther University, Oyo, Nigeria., within the derived savanna agroecological zone of southwest Nigeria. Agronomic data were~~. Data collected at key growth stages and harvesting were analyzed using R statistical software ~~v4~~version 4.4.2. ~~Significant~~Hybrids exhibited significant ($p < 0.$~~001) phenotypic~~05) differences ~~were observed among hybrids~~ for grain yield, ear height, plant aspect, ear aspect, southern corn leaf blight, and ~~FAW~~foliar fall armyworm damage at 8 and 12 weeks~~.~~ after planting. Notably, ~~FAWSYN-1/(TZLComp. 1 C6-W-39-1-1)-B-B), Oba Super 11, FAWSYN-2/(TZLComp. 1 C6-W-39-1-1)-B-B, FAWSYN-1/IITATZI2305, and FAWSYN-1/TZISTR1121~~five experimental hybrids exhibited superior performance, combining high yield with desirable agronomic traits and low ~~FAW~~foliar fall armyworm damage. FAWSYN-1/(TZLComp. 1 C6-W-39-1-1)-B-B~~)~~, followed closely by FAWSYN-1/IITATZI2305, FAWSYN-2/(TZLComp. 1 C6-W-39-1-1)-B-B, FAWSYN-1/TZISTR1121, and FAWSYN-~~1~~3/IITATZI2305 recorded the highest grain ~~yield~~yields across environments. ~~These superior hybrids are recommended for~~When further ~~multi-environment testing~~tested for performance stability across more diverse agro-ecologies~~ in Nigeria before their release to~~, the selected hybrids may be released as maize cultivars to mitigate fall armyworm attacks among Nigerian farmers.

Formatted: Font: Not Bold

Introduction

Maize (Zea mays~~ L.)~~) is one of the most widely cultivated and important cereal crops globally, serving as a staple food for millions of people and as a vital component ~~in~~of animal feed, forage and industrial applications (1~~). Annual~~,2). The annual global production has surpassed ~~1147 million~~1.147 billion tons (~~2~~3). Its adaptability to diverse agro-climatic conditions enables cultivation across various regions, with leading producers ~~including~~being the United States, China, Brazil, Argentina, and India (~~3).~~4). However, achieving consistent productivity depends on the development and systematic evaluation of improved hybrids, as grain yield and related agronomic traits can vary significantly among genotypes and across growing seasons, particularly under rainfed conditions (2). It remained a key ~~food~~ source of food and economic ~~livelihood~~livelihoods for approximately 300 million smallholder farmers across ~~sub~~Sub-Saharan Africa (~~4). Maize production in Nigeria reached 12.75 million metric tons in 2021~~SSA) (5).

Maize production in Nigeria reached 12.75 million metric tons in 2021 (6). Despite its critical role~~, maize production~~ in ~~Nigeria~~the country, the crop faces multiple constraints, ~~especially~~particularly biotic stresses~~ such as~~, including insect pests and diseases, which cause significant yield reductions. (7). Among these, ~~the fall armyworm (~~*Spodoptera frugiperda*~~)~~, commonly referred to as FAW, an invasive polyphagous pest, has become a major threat since its detection in West and Central Africa (WCA) in 2016 (~~6~~8). The pest feeds voraciously on maize leaves~~ and~~, stalks, tassels, and ears, leading to weakened plants, increased vulnerability to secondary infections, and substantial yield losses~~. Fall armyworm~~ (9). FAW infestations have been associated with annual economic losses estimated ~~between~~at US$ 2.5~~ billion and~~ – 6.2 billion in twelve affected African countries~~.~~, and up to US$ 13 billion in maize, rice, sorghum, and sugarcane across SSA (10,11).~~(7).~~ For instance, Ethiopia experienced a 36%

Formatted: English (United States)

Formatted: Font: Not Italic

Formatted: Ligatures: None

average annual yield loss between 2017 and 2019, attributed to ~~this~~the pest, ~~equating to a~~ resulting in an economic loss of US$ 200 million ~~economic setback (8).~~(12). Similarly, maize yields in Nigeria declined by over 40% due to ~~fall armyworm~~foliar FAW damage~~ (9) underscoring the global impact of this pest.~~. (13).

~~FAW~~The invasion of FAW in Africa has led to increased pesticide use among smallholder farmers, raising concerns about environmental and health risks. Studies ~~show~~have shown that synthetic pesticides remain the most popular management option, with usage increasing up to 3-fold in some areas (~~10). Consequently, breeding~~14). However, the calamitous environmental impacts of pesticide use, coupled with the rising costs of the chemicals, which are becoming increasingly unaffordable by resource-poor farmers across SSA, have rendered this strategy unattractive (15,16). Breeding maize hybrids with enhanced resistance or tolerance to ~~fall armyworm~~FAW has emerged as a sustainable strategy to mitigate crop losses and reduce ~~pesticide dependency.~~reliance on pesticides (17,18). Institutions such as the International Institute of Tropical Agriculture (IITA) and the International Maize and Wheat Improvement ~~Center~~Centre (CIMMYT) have made substantial progress in developing ~~fall armyworm~~FAW-resistant maize varieties using a combination of traditional breeding and molecular tools (~~11~~19).

The derived savanna agro-ecology of southwestern Nigeria is an important maize-growing region characterized by bimodal rainfall patterns and moderate temperatures conducive to maize cultivation~~.~~ (2). However, its climatic conditions also ~~favour fall armyworm~~favor FAW resurgence and proliferation, posing significant challenges to maize productivity in ~~this~~the zone~~ (12~~. (20). While several resistant FAW maize hybrids have been developed and evaluated, their performance and adaptability within the derived savanna ~~environment~~agro-ecology of Nigeria remain understudied~~.~~ (21–23). Systematic evaluation of

these hybrids for yield, pest resistance, and agronomic traits is essential to guide the identification and selection ~~and distribution.~~

of superior varieties. Thus, the objectives of this study ~~are~~were to: (i) assess the agronomic performance and variation among ~~fall armyworm~~new, IITA-developed FAW-resistant maize hybrids in the minor cropping season, (ii) ~~investigate~~determine the association between grain yield and other desirable agronomic traits, (iii) identify key traits influencing hybrid performance, and (iv) select superior maize hybrids with enhanced yield and ~~fall armyworm~~FAW tolerance.

Formatted: Ligatures: None

Formatted: Ligatures: None

Formatted: Indent: First line: 0"

Formatted: English (United States)

Materials and methods

~~Plant~~Genetic materials, experimental ~~site~~Site, and ~~cultivation~~field establishment

The ~~plant~~genetic materials evaluated in this study consisted of ~~twenty-six fall armyworm~~26 FAW-resistant maize hybrids and ~~six~~6 commercial checks obtained from the International Institute of Tropical Agriculture (IITA), Ibadan, Nigeria (Table 1). ~~These~~The 26 experimental hybrids represent genetically diverse materials bred specifically for tolerance or resistance to ~~fall armyworm (*Spodoptera frugiperda*), alongside commonly used commercial varieties for comparative purposes.~~FAW. Field trials were conducted in 2024 at two environments within the derived savanna agro-ecology of southwestern Nigeria at the Teaching and Research Farm of Ladoke Akintola University of Technology (LAUTECH), Ogbomoso (8.172°N, 4.274°E), and the Teaching and Research Farm of Ajayi Crowther University (ACU), Oyo (7.734°N, 4.064°E).

Table 1. ~~Fall armyworm~~List of the 26 FAW-resistant synthetic females top-cross maize hybrids and 6 commercial hybrid ~~and~~ checks evaluated in two locations in the derived savanna agro-ecology in the minor cropping seasons of 2024 in Nigeria.

Formatted: Font: Not Bold

~~Entry~~Hybrid ID	~~Genotype~~Pedigree	~~Source~~
**G1**	FAWSYN-1/(TZLComp. 1 C6-W-39-1-1)-B-B	~~IITA~~
**G2**	FAWSYN-1/TZISTR1869	~~IITA~~
**G3**	FAWSYN-1/TZISTR1878	~~IITA~~
**G4**	FAWSYN-1/TZISTR1121	~~IITA~~
**G5**	FAWSYN-1/TZISTR1129	~~IITA~~
**G6**	FAWSYN-1/TZISTR2024	~~IITA~~
**G7**	FAWSYN-1/TZISTR1305	~~IITA~~
**G8**	FAWSYN-1/TZISTR2042	~~IITA~~
**G9**	FAWSYN-2/(TZLComp. 1 C6-W-39-1-1)-B-B	~~IITA~~
**G10**	FAWSYN-2/TZISTR1878	~~IITA~~
**G11**	FAWSYN-2/TZISTR1129	~~IITA~~
**G12**	FAWSYN-2/TZISTR2024	~~IITA~~
**G13**	FAWSYN-2/TZISTR1305	~~IITA~~
**G14**	FAWSYN-2/TZISTR2129-2	~~IITA~~
**G15**	FAWSYN-3/TZISTR1869	~~IITA~~
**G16**	FAWSYN-3/TZISTR1872	~~IITA~~
**G17**	FAWSYN-3/TZISTR1878	~~IITA~~
**G18**	FAWSYN-3/IITATZI2300	~~IITA~~
**G19**	FAWSYN-3/IITATZI2305	~~IITA~~
**G20**	FAWSYN-1/IITATZI2300	~~IITA~~
**G21**	FAWSYN-2/IITATZI2300	~~IITA~~
**G22**	FAWSYN-1/IITATZI2305	~~IITA~~
**G23**	FAWSYN-2/IITATZI2305	~~IITA~~
**G24**	FAWSYN-1	~~IITA~~
**G25**	FAWSYN-2	~~IITA~~
**G26**	FAWSYN-3	~~IITA~~
**G27**	SAMMAZ 51	~~IITA~~
**G28**	Oba Super 11	~~IITA~~
**G29**	SC301	~~IITA~~
**G30**	Oba Super 7	~~IITA~~
**G31**	Oba Super 9	~~IITA~~
**G32**	Oba Super II	~~IITA~~

~~Field trials were conducted in 2024 at two environments within the Derived savanna agro-ecology of southwestern Nigeria, the teaching and research farms of Ladoke Akintola University of Technology (LAUTECH), Ogbomoso (8.172°N, 4.274°E), and Ajayi Crowther University (ACU), Oyo (7.734°N, 4.064°E).~~ Both environments experience bimodal rainfall, with ~~an~~ average annual precipitation ranging ~~between~~from 1,000 ~~and~~to 1,200 mm and mean temperatures of 28 to 30 °C during the major and minor maize~~-~~-growing ~~season.~~seasons (24). Six months of weather data observed at Ogbomoso during the study period from May – October~~,~~ 2024 ~~is~~are shown in Fig 1. These climatic conditions, typical of the ~~Derived~~derived savanna agro-ecological zone, provide an ideal environment for assessing the ~~adaptability~~agronomic performance and pest resistance of maize hybrids under natural ~~fall armyworm~~FAW pressure.

Fig 1. The weather data for May to October, 2024. Source: weather station, Faculty of Agricultural Sciences, LAUTECH, Ogbomoso.

The experimental ~~fields were prepared by ploughing~~field at each location was ploughed and ~~harrowing~~harrowed two weeks before planting to optimize soil conditions. ~~The maize~~Experimental hybrids and checks were planted in July 2024 during the ~~main rainy~~minor cropping season ~~under rainfed conditions. The trials employed~~. Entries were laid out in an ~~8x4~~8×4 alpha-lattice design with three replicates. Each plot ~~measured~~was 4 ~~meters~~m in length with row spacing of 0.75 ~~meters~~m and intra-row spacing of 0.5 ~~meters, establishing~~m. Three seeds were planted in each hill, and the plants were thinned to two per stand 2 weeks after planting (WAP) to attain a plant population density of ~~approximately~~ 53,333 plants ~~per hectare after thinning.~~/ha. Standard agronomic practices, including fertilizer application and weed

Formatted: Indent: First line: 0"

Formatted: Font: Not Bold

control, were ~~followed~~done to ensure optimal crop growth. No pesticides were used to control FAW infestation throughout each experiment.

Data collection

Data ~~collection focused~~ on key agronomic traits, foliar diseases, and foliar fall armyworm damage ~~(FAWD)~~ at critical maize growth stages ~~to evaluate~~ and at harvest were recorded in accordance with the ~~performance~~conduct and management of ~~the~~ maize ~~hybrids under study.~~field trials (25). Phenological traits, including days to pollen shed and days to silking, were recorded as the number of days from planting ~~until~~to when 50% of plants ~~within~~in each plot ~~reached these reproductive stages.~~ began shedding pollen and emerged with receptive silks, respectively. The anthesis-silking interval was calculated by subtracting the number of days to pollen from the number of days to silking, providing insight into flowering synchrony, which affects grain yield (~~13).~~

25). Plant ~~height~~ and ear ~~height~~heights were measured using a meter rule from the base of the plant to the first tassel branch and to the first node bearing the upper ear, respectively. Measurements were taken on ten representative plants per plot to calculate average values ~~(13).~~. Plant ~~and ear aspects were scored~~ aspect was rated visually on a scale of 1 to 5 ~~scale~~, where ~~a lower score indicates better visual quality and plant health.~~ 1 = excellent overall phenotypic appearance and 5 = poor overall phenotypic appearance. Ear aspect was also scored visually on a scale of 1 to 5, where 1 = clean, uniform, large, and well-filled ears, and 5 = rotten, variable, small, and partially filled ears. Husk cover was ~~similarly scored, assessing the protection of~~assessed on a scale of 1 to 5, where 1 = husks tightly arranged and extended beyond the ear ~~from pests~~tip, and ~~environmental damage~~5 = ear tips fully exposed. Ear rot was evaluated using a scale of 1 to 5 ~~scale~~, with 1 indicating no rot and 5 severe infection (~~13).~~

Formatted: Ligatures: Standard + Contextual
Formatted: Ligatures: Standard + Contextual
Formatted: Ligatures: Standard + Contextual
Formatted: Ligatures: Standard + Contextual
Formatted: Ligatures: Standard + Contextual
Formatted: Ligatures: Standard + Contextual
Formatted: Ligatures: Standard + Contextual
Formatted: Ligatures: Standard + Contextual
Formatted: Ligatures: Standard + Contextual
Formatted: Ligatures: Standard + Contextual
Formatted: Ligatures: Standard + Contextual
Formatted: Ligatures: Standard + Contextual
Formatted: Ligatures: Standard + Contextual
Formatted: Ligatures: Standard + Contextual
Formatted: Ligatures: Standard + Contextual
Formatted: Ligatures: Standard + Contextual
Formatted: Ligatures: Standard + Contextual
Formatted: Ligatures: Standard + Contextual
Formatted: Ligatures: Standard + Contextual
Formatted: Ligatures: Standard + Contextual
Formatted: Ligatures: Standard + Contextual
Formatted: Ligatures: Standard + Contextual
Formatted: Ligatures: Standard + Contextual
Formatted: Ligatures: Standard + Contextual
Formatted: Ligatures: Standard + Contextual
Formatted: Ligatures: Standard + Contextual
Formatted: Ligatures: Standard + Contextual
Formatted: Ligatures: Standard + Contextual
Formatted: Ligatures: Standard + Contextual
Formatted

22). Foliar diseases evaluated ~~included Maize Streak Virus~~include maize streak virus (MSV), ~~Southern Corn Leaf Rust~~southern corn leaf rust (SCLR), ~~Southern Corn Leaf Blight~~southern corn leaf blight (SCLB), and ~~Curvularia Leaf Spot~~*curvularia* leaf spot (CLS). Each disease was scored on a 1~~ to ~~-5 scale based on symptom severity, ~~where~~with 1 ~~represented~~indicating no visible symptoms and 5 indicating a severe infection (~~13). Fall armyworm~~15). Foliar FAW damage was assessed at 4, 8, and 12 weeks after ~~sowing~~planting (WAP) using a modified Davis scale ranging ~~from~~between 1 (no visible damage) ~~to~~and 9 (severe damage with heavily destroyed leaves) (~~14). This allowed monitoring of pest pressure progression during critical vegetative and reproductive growth stages.~~

26). All ears were harvested per plot, field weight and grain weight were taken, and the percent grain moisture at harvest was determined using a moisture meter. Grain yield ~~data were obtained by harvesting ears from each plot, calculating~~(Kg/ha) was computed using total ~~grain~~field weight~~, and adjusting~~ adjusted to ~~a standard~~15% moisture content~~ of 15%. A~~, and 80% shelling percentage~~ of 80% was assumed to convert ear weight to grain yield per hectare. Grain moisture content was measured using a digital moisture meter (13~~(22).

$$GrainYield(kg/ha) = \left(\frac{earweight(kg/plot)}{\text{plot area (m}^2\text{)}}\right) \times 0.80 \times \left(\frac{(100 - \text{MC})}{85}\right) \times 10{,}000$$

Statistical data analysis

~~All collected data was entered into Microsoft Excel 2019 before analysis.~~ Analysis of variance (ANOVA) was performed using the Multi-environment Trials Analysis ~~-~~(metan) package in R version 4.4.2 (~~15~~27) to ~~examine~~ determine the differences among the hybrids, and ~~significant differences~~trait means were ~~verified~~separated with~~ the~~ Fisher's protected Least Significant Differences (LSD) test at 5% probability level. The ANOVA model ~~included fixed effects for genotypes and environments,~~treated all factors (genotype, environment, genotype × environment interaction, replicate within environment, and block within replicate and

Formatted: Ligatures: Standard + Contextual
Formatted: Ligatures: Standard + Contextual
Formatted: Ligatures: Standard + Contextual
Formatted: Ligatures: Standard + Contextual
Formatted: Ligatures: Standard + Contextual
Formatted: Ligatures: Standard + Contextual
Formatted: Ligatures: Standard + Contextual
Formatted: Ligatures: Standard + Contextual
Formatted: Ligatures: Standard + Contextual
Formatted
Formatted: Ligatures: Standard + Contextual
Formatted: Ligatures: Standard + Contextual
Formatted: Ligatures: Standard + Contextual
Formatted: Ligatures: Standard + Contextual
Formatted: Ligatures: Standard + Contextual
Formatted: Ligatures: Standard + Contextual
Formatted: Ligatures: Standard + Contextual
Formatted: Ligatures: Standard + Contextual
Formatted: Ligatures: Standard + Contextual
Formatted: Ligatures: Standard + Contextual
Formatted
Formatted: Ligatures: Standard + Contextual
Formatted: Ligatures: Standard + Contextual
Formatted: Indent: First line: 0"
Formatted: English (United States)
Formatted: Indent: First line: 0", Space After: 8 pt
Formatted: Ligatures: Standard + Contextual
Formatted: Ligatures: Standard + Contextual
Formatted: Ligatures: Standard + Contextual
Formatted: Ligatures: Standard + Contextual
Formatted: Ligatures: Standard + Contextual

environment) as random effects~~ for blocks and replicates nested within environments,~~ expressed as~~ yijk = μ + ek + bj(k) + αi + (eα)ik + eijk,~~: $y_{ijkl} = \mu + g_i + e_j + (ge)_{ij} + r_{k(j)} + b_{l(kj)} + \varepsilon_{ijkl}$, where ~~yijk~~$y_{ijkl}$ is the observed value of a response variable for the ~~i-th hybrid, measured~~ ith genotype evaluated in the ~~j-th block within the k-th environment,~~ jth environment, replicate k within environment j, and block l within replicate k and environment j; μ is the overall mean~~, bj(k)~~; g_i is the effect of the ith genotype; e_j is the ~~block within~~random effect of the jth environment~~, αi~~; $(ge)_{ij}$ is the effect of the ~~i-th hybrid, (eα)ik is the~~ genotype × environment interaction~~,~~; $r_{k(j)}$ is the effect ~~between~~of replicate k nested within environment j; $b_{l(kj)}$ is the ~~i-th hybrid~~effect of block l nested within replicate k and ~~the k-th~~ environment~~,~~ j; and ~~eijk~~ε_{ijkl} is the residual error ~~term.~~ (28).

~~Variance components for~~The genotypic variance ~~σ_g^2~~σ_g^2, genotype~~-by-~~ × environment interaction variance (~~σ_{ge}^2),~~ σ_{ge}^2), and residual error variance (~~σ_e^2), and broad-sense heritability (H²)~~σ_e^2) were ~~analysed~~estimated using META-R v6.0 (~~16).~~ 29). The Principal Component Analysis (PCA) was ~~calculated~~performed using the "prcomp" function in R version 4.4.2 to reduce the dimensionality and identify the most significant traits contributing to ~~genetic variation among~~the variability of the hybrids. Those PCs with ~~Eigenvalues~~eigenvalues >1 were selected (~~17).~~

~~Pearson's~~30). Spearman's correlation analysis was computed to determine the associations among all measured traits ~~measured~~ using the "metan" package in R (~~15).~~ 27). Genotype-by-yield* × trait (GYT) analysis and biplot ~~was analysed~~were visualized in R 4.4.2 to ~~simultaneously~~ evaluate multiple traits in relation to grain yield simultaneously and to rank hybrids for superiority ~~index ranking of hybrids~~ based on multi-trait performance (~~18~~31,32).

Formatted: Ligatures: Standard + Contextual

Formatted: Ligatures: Standard + Contextual

Formatted: Ligatures: Standard + Contextual

Formatted: Ligatures: Standard + Contextual

Formatted: Ligatures: Standard + Contextual

Formatted: Ligatures: Standard + Contextual

Formatted: Ligatures: Standard + Contextual

Formatted: Ligatures: Standard + Contextual

Formatted: Ligatures: Standard + Contextual

Formatted: Ligatures: Standard + Contextual

Formatted: Ligatures: Standard + Contextual

Formatted: Ligatures: Standard + Contextual

Formatted: Ligatures: Standard + Contextual

Formatted: Ligatures: Standard + Contextual

Formatted: Ligatures: Standard + Contextual

Formatted: Ligatures: Standard + Contextual

Formatted: Ligatures: Standard + Contextual

Formatted: Ligatures: Standard + Contextual

Formatted: Ligatures: Standard + Contextual

Formatted: Font: Not Italic

Results

Analysis of variance

~~The~~Results of analysis of variance ~~is presented~~shown in Table 2~~, which~~ indicated ~~highly~~ significant hybrid effects ($p < 0.05$) for grain yield ~~($p < 0.001$), ear height ($p < 0.01$),~~, plant aspect, ~~and ear aspect ($p < 0.05$). No significant variation was observed for plant height, husk cover, ears per plant, and ear rot. Among the foliar diseases, only~~ear aspect, southern corn leaf blight ~~demonstrated a significant hybrid effect ($p < 0.01$). Fall armyworm~~, *curvularia* leaf spot, and foliar FAW damage ~~exhibited significant variation among hybrids~~ at 8 and 12 weeks after ~~sowing~~planting (WAP). Significant hybrid × environment interaction effects ($p < 0.05$~~), but not at 4 weeks.~~) were observed for plant aspect, ear aspect, southern corn leaf blight, and foliar FAW damage at 8 WAP. Environmental effects were significant ($p < 0.05$) for all traits except grain yield and ear aspect. The coefficients of variation (CV) were below 20% for ~~traits such as~~ plant ~~height (14.07%) and~~ aspect, ear aspect, ears per plant ~~(14.78%), indicating data consistency~~, and *curvularia* leaf spot. In contrast, grain yield, plant height, ear height, husk cover, ~~and~~ ear rot ~~displayed CVs exceeding~~, maize streak virus, southern corn leaf rust, southern corn leaf blight, and foliar FAW damage at 4, 8 and 12 WAP had CV values above 20~~%, suggesting greater variability, potentially attributable to environmental factors or measurement subjectivity.~~%. The ~~coefficient~~coefficients of determination (R^2) ~~was~~ were generally moderate to high (0.54 – 0.91) for most of the traits

Mean performance

The mean performance of~~, including 0.78 for grain yield, indicating that~~ the ~~model effectively captured~~evaluated hybrids is shown in Table 3. Grain yield ranged between

Formatted: English (United States)

Formatted: English (United States)

Formatted: Ligatures: Standard + Contextual (×7)

Formatted: Ligatures: Standard + Contextual (×3)

Formatted: Font: Bold, Ligatures: Standard + Contextual

Formatted: Ligatures: Standard + Contextual (×7)

Formatted: Ligatures: Standard + Contextual (×5)

Formatted: Ligatures: Standard + Contextual

1,452.76 Kg/ha in FAWSYN-2 and 3,127.84 Kg/ha in FAWSYN-1/(TZLComp. 1 C6-W-39-1-1)-B-B, with a trial mean of 2,185.98 Kg/ha. Relative to the observed variability.

Formatted: Ligatures: Standard + Contextual

best commercial check, Oba Super 11 with 2,979.36 Kg/ha, the top-performing hybrid showed a 5% yield advantage. Plant height ranged from 139.77 cm in FAWSYN-2/TZISTR2129-2 to 309.83 cm in FAWSYN-2/IITATZI2305. Ear height ranged from 64.88 cm in FAWSYN-1 to 156.04 cm in FAWSYN-1/TZISTR1305. Husk cover scores (1–5) ranged from 2.50 in FAWSYN-1/TZISTR2042 to 3.58 in FAWSYN-1/(TZLComp. 1 C6-W-39-1-1)-B-B and FAWSYN-1/TZISTR1305. Plant aspect scores (1–5) ranged from 3.08 in FAWSYN-1/TZISTR1121 to 4.33 in FAWSYN-1/TZISTR1305. Ears per plant ranged from 0.73 in FAWSYN-1/TZISTR1129 to 1.05 in FAWSYN-3/IITATZI2300. Ear aspect scores (1–5) ranged from 2.75 in FAWSYN-1/IITATZI2305 to 4.00 in SC301. Ear rot scores (1–5) ranged from 2.58 in FAWSYN-2/TZISTR1129, FAWSYN-3/TZISTR1878, and FAWSYN-1/TZISTR1305 to 3.42 in FAWSYN-1/TZISTR2024, FAWSYN-2/TZISTR2024, SC301, and Oba Super 9. Maize streak virus scores (1–5) ranged from 1.17 in FAWSYN-3/TZISTR1869 and SAMMAZ 51 to 2.17 in FAWSYN-1/TZISTR1121. Southern corn leaf rust scores (1–5) ranged from 1.33 in Oba Super 11 to 2.25 in FAWSYN-1/IITATZI2305 and FAWSYN-2/IITATZI2300. Southern corn leaf blight scores (1–5) ranged from 1.33 in FAWSYN-1/TZISTR2024 to 3.17 in FAWSYN-1/IITATZI2305. *Curvularia* leaf spot scores (1–5) ranged from 2.08 in Oba Super 11 to 4.50 in FAWSYN-3/TZISTR1878 and Oba Super II. Foliar FAW damage ratings (1–9) at 4 WAP ranged from 2.00 in FAWSYN-1/(TZLComp. 1 C6-W-39-1-1)-B-B, FAWSYN-1/TZISTR2042, Oba Super 11, and Oba Super 9 to 4.00 in FAWSYN-3/TZISTR1872 and SC301. At 8 WAP, it ranged from 2.67 in FAWSYN-1, FAWSYN-3/IITATZI2300, FAWSYN-1/TZISTR1305, FAWSYN-3/TZISTR1872, and Oba Super 7 to 4.00 in FAWSYN-1/TZISTR1878, SC301, and Oba Super II, while at 12 WAP, it ranged from 2.33 in FAWSYN-1/TZISTR2042 to 4.17 in FAWSYN-2.

Table 2. Mean squares of grain yield, ~~foliar diseases and~~ other agronomic ~~trats of fall armyworm~~ traits, foliar diseases, and foliar FAW damage of FAW-resistant maize ~~hybrid~~hybrids and checks evaluated across two environments in the derived savanna agro-ecology of Nigeria during the minor season of 2024.

Formatted: Indent: Left: 0", Line spacing: single

Formatted: Font: Not Bold

Source of Variation	Dfdf	GY (kgKg/ha)	PH (cm)	EH (cm)	HC (1-5)	PASP (1-5)	EPP (count)	EASP (1-5)	EROT (1-5)
HybridEnvironment	131	944986.05***157331.98	905055.83***4872.88	200494.29***1457.97	0.4523.31***	0.56*6.75***	0.033.57***	0.2945*	2.88*0.47
Replicate (Hybrid × Environment)	431	8640746.71***458714.73	3377.46***5795.06	500.18***1433.73	2.80***0.58	3.10***0.50*	0.14***02	0.48*1.90***	1.30*0.43
Block(Environment*Replicate)	421	157331.987492 34**	262.6373310.09***	111.8319528.27***	0.8023.31***	0.60**6.75***	3.57***0.02	0.2829	0.512.88*
HybridReplicate (Environment)	314	9449868640746.71***	15410090.06	77**1118.13	0.442.80***	3.10***0.56*	0.0314***	1.90**0.45*	0.471.30*
Block (Hybrid*Environment × Replicate)	3142	458715749233.82**	163*5804.50	77**1772.87	0.5880	0.50*6**	0.0302	0.48*28	0.4351
Error	82	361893361892.90	98.205417.27	36.441545.23	0.54	0.31	0.02	0.28	0.44
R^2		0.7879	0.9961	0.9960	0.7069	0.7375	0.82	0.68	0.6561
CV (%)		27.6752	14.0746.4	18.2052.02	23.7224.12	16.1615.32	14.7884	16.5057	2122.02

Source of Variation	Dfdf	MSV (1-5)	SCLR (1-5)	SCLB (1-5)	CLS (1-5)	FAW D4FF AWD4 (1-9)	FAW D8FF AWD8 (1-9)	FAW D12 FFA WD12 (1-9)		
HybridEnvironment	131	2.88*0.42	1.88*0.36	0.93**35.88***	0.63*232.98***	8.33** 1.28	1.57*18 0.19***	1.42* 292.5 5***		
Replicate (Hybrid × Environment)	431	0.0846	0.77*38	2.181.03**	0.1048	8.60** *1.39	11.13* **1.59*	3.29** 1.12		
Block(Environment*Replicate)	421	0.482.88*	1.88*	35.88***	232.98***	08.33**	180.19* **0.75*	0.27 292.5 5***	1.13	1.92** 1.62**
HybridReplicate (Environment)	314	0.4208	0.3677*	0.932.18**	0.6310	1.288.60***	1.57*1 1.13***	3.29** 1.42*		
Block (Hybrid*Environment × Replicate)	3142 0.46 0.38 1.03**	0.48	1.390.33	1.560.75*	1.120.27	1.13	1.92**	1.62**		
Error	82	0.52	0.29	0.45	0.33	1.01	0.93	0.86		
R^2		0.5354	0.6564	0.79	0.6491	0.68	0.84	0.9187		
CV (%)		45.1744.49	30.2	29.9418	17.29.24 80.98	28.0849	32.6585	23.6929.09		

*, **, *** = Significance at 0.05, 0.01, and 0.001 probability levels respectively; GY = Graingrain yield (kg/ha);; PH = Plantplant height (cm);; EH = Earear height (cm);; HC = Huskhusk cover; PASP = Plantplant aspect; EPP = Earsears per plant; EASP = Earear aspect; EROT = Earear rot; MSV = Maizemaize streak virus severity; SCLR = Southernsouthern corn leaf rust; SCLB = Southernsouthern corn leaf blight; CLS = Curvularia*curvularia* leaf spot; FAWD4, FAWD8, FAWD12 = Fall armywormFFAWD4, FFAWD8, FFAWD12 = foliar FAW damage ratings at 4, 8, and 12 weeks,WAP; df = degreedegrees of freedom,; R^2 = coefficient of determination,; CV (%) = coefficient of variation.

Formatted: Indent: Left: 0.06", Space Before: 12 pt, Line spacing: Double

Mean performance

The mean performance of the evaluated hybrids is shown in Table 3. Grain yield ranged from 1,452.76 kg/ha in FAWSYN-2 to 3,331.71 kg/ha in FAWSYN-1/(TZLComp. 1 C6-W-39-1-1)-B-B (G1). The commercial check Oba Super 11 (G28) yielded 2,979.36 kg/ha, comparable to several resistant hybrids. Plant height varied from 62.08 cm to 81.12 cm, with G1 tallest, while ear height ranged from 27.04 cm in Oba Super II (G32) to 41.00 cm in G9. Husk cover scores (1–5) ranged from 2.50 to 4.33, with FAWSYN-2/TZISTR2042 (G8) showing the best cover, while ears per plant ranged from 0.73 to 1.05, highest in FAWSYN-3/IITATZI2300 (G18). Maize streak virus scores ranged from 1.17 in FAWSYN-3/TZISTR1869 and Oba Super 11 (G28) to 2.08 in FAWSYN-2/IITATZI2305 (G23). Southern corn leaf rust and blight were lowest in Oba Super 11 and FAWSYN-3/TZISTR1872, respectively. Curvularia leaf spot varied from 2.08 in Oba Super II to 3.75 in G8. Fall armyworm damage ranged from 2.00 in G1 and G28 to 4.50 in Oba Super II at 4 weeks; at 8 weeks it was lowest in G1 and G28 (2.00) and highest in SC301 (G29) (4.00), while at 12 weeks it ranged from 2.50 in FAWSYN-1/TZISTR2024 to 4.17 in FAWSYN-2.

Table 3. Mean performance of FAW-resistant maize hybrids and checks for grain yield, other agronomic traits, foliar diseases, and foliar FAW infestation scoresdamage across environments in 2024.

HYBRID	GY (kgKg/ha)	PH (cm)	EH (cm)	HC (1-5)	PASP (1-5)	EPP (count)	EASP (1-5)	EROT (1-5)	MSV (1-5)	SCLR (1-5)	SCLB (1-5)	CLS (1-5)	FAWD4 FFAWD4 (1-9)	FAWD8 FFAWD8 (1-9)	FAWD12F FAWD12 (1-9)
FAWSYN-1/(TZLComp. 1 C6-W-39-1-1)-B-B	3127.84	81.12165.11	40.8576.98	3.58	3.17	0.93	2.92	3.00	1.25	2.00	2.17	3.58	3.00	2.00	3.33
FAWSYN-2/(TZLComp. 1/TZISTR1869 C6-W-39-1-1)-B-B	1968.11 2842.10	75.06170.23	33.2787.04	3.002.83	3.6725	0.7895	3.3308	3.082.67	1.7525	1.672.17	2.1733	3.3350	3.5033	3.332.83	3.0017
FAWSYN-1/TZISTR1878IITATZI2305	2717.11 2193.70	74.98155.94	36.5368.97	2.833.08	3.6733	0.9097	3.332.75	3.172.75	1.92	2.251.50	2.673.17	3.3325	4.333.67	32.50	4.002.50
FAWSYN-3/IITATZI2305	2632.29	162.35	79.39	2.58	3.58	0.96	3.08	3.08	1.42	1.50	2.25	3.58	3.33	2.67	3.33
FAWSYN-1/TZISTR1121	2577.07	68.17148.85	30.9170.35	3.00	3.08	0.93	3.08	2.67	2.17	1.75	2.25	3.50	3.50	2.50	2.67
FAWSYN-1/TZISTR1129TZISTR2042	2420.24 1739.74	63.53155.99	30.9274.32	3.422.50	3.7517	0.731.04	3.502.92	2.753.17	1.58	1.502.00	2.0017	2.833.75	2.673.50	3.172.50	3.672.33
FAWSYN-1/TZISTR20243/IITATZI2300	2409.35 2197.74	66156.60	28.9475.92	2.753.00	3.4233	1.050.89	3.5808	3.4233	1.7592	1.5075	1.332.25	3.3350	2.833.17	32.33	2.50
FAWSYN-1/TZISTR13052/IITATZI2300	2343.79 1640.63	63.78156.60	70.8533.78	3.5817	4.333.58	0.8393	3.0017	3.172.58	1.5867	2.251.75	2.5833	3.33	3.3317	2.5067	2.834.00
FAWSYN-1/TZISTR2042IITATZI2300	2323.15 2420.24	161.22 70.85	37.6975.22	2.503.00	3.1725	1.040.93	3.502.92	3.1708	1.5883	2.00	2.17	3.7558	3.172.67	2.5067	2.3383
FAWSYN-2/(TZLComp. 1 C6-W-39-1-1)-B-B3/TZISTR1869	2200.63 2842.11	78.49155.22	41.0079.04	3.002.83	3.2583	0.9596	3.0825	3.08	1.2517	2.171.75	2.33	3.5008	3.3367	3.3317	3.17
FAWSYN-1/TZISTR2024	2197.74	151.08	71.51	3.00	3.42	0.89	3.58	3.42	1.75	1.50	1.33	3.33	2.83	3.33	2.50
FAWSYN-21/TZISTR1878	2193.70 2117.46	74.06157.81	34.6976.46	2.833.00	3.1767	0.9490	3.5033	3.2517	1.8392	1.5850	2.5867	3.1733	4.333.50	3.502.83	34.00
FAWSYN-2/TZISTR1129	2172.43	62.08152.17	27.4566.56	3.00	3.58	0.90	2.92	2.58	1.42	1.58	2.42	3.33	3.83	3.17	3.67
FAWSYN-2/TZISTR2024IITATZI2305	2152.97 1929.36	74.46309.83	35.0665.74	3.252.92	3.8367	0.88	2.833.25	2.833.42	1.752.08	1.7558	2.921.75	3.1750	3.50	2.83	3.0083
FAWSYN-2/TZISTR1305TZISTR1878	2117.46 1740.76	65.10156.39	32.7368.19	3.002.83	4.003.17	0.8794	3.502.83	2.753.25	1.83	1.9258	2.58	3.4217	3.1750	2.6783	3.1750
FAWSYN-3	2048.60	148.01	71.67	3.33	3.75	0.92	3.67	2.92	1.67	1.42	1.75	3.50	3.83	3.17	3.00
FAWSYN-2/TZISTR2129-2	2025.88	67.01139.77	34.5970.56	3.50	3.67	0.89	3.00	3.00	1.67	1.83	2.75	3.25	3.17	2.50	2.67
FAWSYN-31/TZISTR1869	1968.10 2200.63	69.75155.58	35.4870.06	3.00	3.8367	0.9678	3.2533	2.673.08	1.1775	1.7567	2.3317	3.0833	3.6750	2.833.17	3.1750
FAWSYN-3/TZISTR18722/TZISTR2024	1929.36 1639.52	63.86155.64	28.6172.85	3.25	3.834.00	0.8288	3.0825	2.753.42	1.75	1.75	2.081.75	3.5017	3.502.83	2.834.00	3.5000

FAWSYN-3/TZISTR1878	1924.39	72.89157.86	35.9877.23	3.33	3.75	0.83	3.25	2.58	1.75	1.75	2.92	3.33	4.50	3.67	2.67
FAWSYN-3/IITATZI23001	1767.632409.35	74.27142.60	33.3564.88	3.252.75	3.334.00	1.050.83	3.082.92	3.332.83	1.9258	1.752.17	2.2500	3.5042	3.1750	2.3367	23.50
FAWSYN-3/IITATZI23052/TZISTR1305	1740.762632.29	73.75150.08	35.9970.51	2.5883	4.003.58	0.9687	2.833.08	2.753.08	1.4283	1.5092	2.2558	3.5842	3.3317	2.67	3.3317
FAWSYN-1/IITATZI2300TZISTR1129	1739.742323.15	75.75158.57	31.9977.94	3.0042	3.2575	0.9373	2.92	2.753.08	1.8358	2.001.50	2.1700	3.582.83	3.1750	2.673.17	3.672.83
FAWSYN-2/IITATZI23001/TZISTR1305	1640.632343.79	75.09146.94	31.42156.04	3.1758	3.584.33	0.9383	3.1700	3.172.58	1.6758	1.752.25	2.3358	3.33	3.1733	2.6750	2.833.00
FAWSYN-1/IITATZI23053/TZISTR1872	1639.522717.11	68.82147.85	30.8665.24	3.0825	4.003.33	0.9782	3.082.75	2.75	1.9275	2.251.75	3.172.08	3.2550	2.833.67	2.504.00	23.50
FAWSYN-2/IITATZI2305	2152.97	64.47	28.95	2.92	3.67	0.88	2.83	2.83	2.08	1.58	2.92	3.50	3.50	2.83	3.83	
FAWSYN-1	1767.63	67.19	30.07	3.25	4.00	0.83	2.92	2.83	1.58	2.17	2.00	3.42	3.50	2.67	3.50	
FAWSYN-2	1452.76	63.35139.91	29.0566.50	2.75	3.58	0.94	3.42	3.33	1.33	1.83	2.00	3.08	3.33	3.17	4.17
FAWSYN-3Checks	2048.60	69.47	34.54	3.33	3.75	0.92	3.67	2.92	1.67	1.42	1.75	3.50	3.83	3.17	[illegible]
SAMMAZ 51	2236.18	68.57145.62	33.9173.84	2.92	3.75	0.94	3.33	2.67	1.17	1.92	2.58	3.67	4.00	3.67	3.17
Oba Super 11	2979	73.5	32.9077.28	2.83	3.25	0.93	3.25	3.33	1.83	1.33	1.92	2.08	3.67	2.00	3.00

	.36	~~6~~154.74													
**SC301**	1902.07	~~75.76~~165.52	~~29.51~~72.08	3.07	3.58	0.92	4.00	3.42	1.50	2.00	2.67	3.50	4.33	4.00	3.50
**Oba Super 7**	2161.48	~~75.38~~152.77	~~35.76~~77.84	2.92	3.83	0.99	3.17	3.08	1.33	1.58	2.50	3.42	3.83	2.67	4.00
**Oba Super 9**	2438.78	~~71.99~~154.32	~~37.67~~80.90	2.92	3.58	0.93	3.17	3.42	1.42	1.92	2.50	3.33	3.83	3.00	3.83
**Oba Super II**	1928.	~~6~~4~~.87~~1	~~27.04~~66.31	3.42	4.08	0.97	3.33	3.25	1.25	1.58	1.75	2.58	4.50	3.50	3.33

Formatted: English (United States)
Formatted: English (United States)
Formatted: Normal
Formatted: Font: Not Bold, English (United States)
Formatted: English (United States)
Formatted: English (United States)
Formatted: English (United States)
Formatted: English (United States)
Formatted: Normal
Formatted: Font: Not Bold, English (United States)
Formatted: English (United States)
Formatted: English (United States)
Formatted: English (United States)
Formatted: English (United States)
Formatted: Font: Not Bold, English (United States)
Formatted: English (United States)
Formatted: English (United States)
Formatted: Normal
Formatted: English (United States)
Formatted: English (United States)
Formatted: Normal
Formatted: Font: Not Bold, English (United States)
Formatted: English (United States)
Formatted: English (United States)

	39	45.23												
**Statistics**														
**Mean**	2185.98	158.64	75.57	3.06	3.61	0.91	3.18	3.01	1.62	1.78	2.31	3.32	3.53	2.94	3.18
**LSD ~~(0.05)~~**	~~9~~4~~5.77~~690.93	~~87.52~~84.53	~~41.56~~45.15	~~1.03~~0.85	0.~~83~~64	0.~~25~~16	0.~~68~~61	0.~~80~~76	0.~~81~~83	0.~~66~~62	~~1.09~~0.77	~~1.53~~0.66	1.~~32~~16	1.~~87~~11	1.~~97~~06

GY = ~~Grain~~grain yield ~~(kg/ha);~~; PH = ~~Plant~~plant height ~~(cm);~~; EH = ~~Ear~~ear height ~~(cm);~~; HC = ~~Husk~~husk cover; PASP = ~~Plant~~plant aspect; EPP = ~~Ears~~ears per plant; EASP = ~~Ear~~ear aspect; EROT = ~~Ear~~ear rot; MSV = ~~Maize~~maize streak virus ~~severity~~; SCLR = ~~Southern~~southern corn leaf rust; SCLB = ~~Southern~~southern corn leaf blight; CLS = ~~Curvularia~~*curvularia* leaf spot; ~~FAWD4, FAWD8, FAWD12 = Fall armyworm~~ FFAWD4, FFAWD8, FFAWD12 = foliar FAW damage ratings at 4, 8, and 12 ~~weeks,~~WAP; LSD ~~(0.05)~~) = Least Significant difference at 5% probability level

297

Variance Components ~~and Heritability~~

Variance component analysis (Table 4) ~~revealed~~showed that the phenotypic variance exceeded the genotypic variance ~~across~~for all traits, with grain yield ~~showing~~having the highest genotypic variance (~~44,159.79~~44145.37) and phenotypic variance (~~387,203.12), indicating moderate genetic variability among hybrids.~~387199.03). Grain yield had ~~moderate broad-sense heritability (44%), with~~ a Phenotypic Coefficient of Variation (PCV) of 14.56% and a Genotypic Coefficient of Variation (GCV) of 9.61%. Other traits, such as plant height, ear height, husk cover, plant and ear aspects, and ears per plant, often showed low ~~or negligible~~ genotypic variance ~~and heritability~~, often close to zero. *Curvularia* leaf spot and foliar fall armyworm damage at 12 ~~weeks recorded~~WAP resulted in GCV values of 4.74% and 4.21%, respectively. PCV values were ~~consistently~~ higher than the GCV~~, confirming strong environmental effects~~.

Formatted: English (United States)
Formatted: English (United States)
Formatted: Ligatures: Standard + Contextual
Formatted: Ligatures: Standard + Contextual
Formatted: Ligatures: Standard + Contextual
Formatted: Ligatures: Standard + Contextual
Formatted: Ligatures: Standard + Contextual
Formatted: Ligatures: Standard + Contextual
Formatted: Ligatures: Standard + Contextual
Formatted: Ligatures: Standard + Contextual
Formatted: Ligatures: Standard + Contextual
Formatted: Ligatures: Standard + Contextual
Formatted: Ligatures: Standard + Contextual
Formatted: Ligatures: Standard + Contextual
Formatted: Ligatures: Standard + Contextual
Formatted: Font: Italic, Ligatures: Standard + Contextual
Formatted: Ligatures: Standard + Contextual
Formatted: Ligatures: Standard + Contextual
Formatted: Ligatures: Standard + Contextual
Formatted: Ligatures: Standard + Contextual
Formatted: Ligatures: Standard + Contextual
Formatted: Ligatures: Standard + Contextual

Table 4. Variance components ~~of~~for grain yield, ~~foliar diseases, FAW ratings, and other~~ agronomic traits, foliar disease scores, and foliar FAW damage across environments in 2024.

Formatted: Caption, Keep with next

Trait	σ²g	σ²ge	σ²e	σ²p	GCV %(%)	PCV %(%)	H²
**Grain yield (kgKg/ha)**	44159.7944145.37	0.00	343043.3334 3053.66	387203.1238 7199.03	9.61	14.56	0.44
**Plant height (cm)**	0.00 7.36 116.41 123.76	0.00	6.825383.13	5383.13	0.00	46.25	
**Ear height (cm)**	0.0600 7.97 38.87 46.90	0.7500	9.781554.13	1554.13	0.0100	52.17	
**Husk cover (1–5)**	0.00	0.00	0.55	0.55	0.00	9.8824.20	0.00
**Plant aspect (1–5)**	0.00	0.03	0.31	0.34	0.00	7.0815.39	0.00
**Ears per plant (count)**	0.00	0.00	0.02	0.02	1.91	6.77	0.08
**Ear aspect (1–5)**	0.00	0.05	0.26	0.31	0.00	8.1915.86	0.00
**Ear rot (1–5)**	0.00	0.00	0.40	0.40	1.83	8.76	0.04
**Maize streak virus (1–5)**	0.00	0.00	0.47	0.47	0.00	17.1942.10	0.00
**Southern corn leaf rust (1–5)**	0.00	0.02	0.30	0.32	0.00	14.0030.92	0.00
**Southern corn leaf blight (1–5)**	0.00	0.16	0.47	0.63	0.00	17.2229.73	0.00
***Curvularia* leaf spot (1–5)**	0.02	0.06	0.30	0.39	4.74	9.76	0.24
**Foliar FAW damage at 4 weeksWAP (1–9)**	0.01	0.05	0.94	1.00	2.07	12.22	0.03
**Foliar FAW damage at 8 weeksWAP (1–9)**	0.00	0.03	0.83	0.87	0.00	13.42	0.00
**Foliar FAW damage at 12 weeksWAP (1–9)**	0.02	0.00	0.83	0.85	4.21	12.44	0.11

σ²g: **Genotypic** = genotypic variance; σ²p: **Phenotypic** = phenotypic variance; σ²e: **Environmental** = environmental variance; GCV %:(%) = Genotypic Coefficient of Variation; PCV %:(%) = Phenotypic Coefficient of Variation; **H²: Heritability (broad-sense).**.

Formatted: footnote, Indent: First line: 0"

Principal Component Analysis

Principal component analysisComponent Analysis (PCA) revealed keyshowed the contribution of grain yield, agronomic traits driving variability, foliar diseases, and foliar FAW damage to variation among the maize hybrids and checks evaluated across environments (Table 5). The first five principal components (PC) cumulatively accounted for 69% of the total variation. The PC1 explained 7122% of the variation. PC1 (26%) was associated with positive an eigenvalue of 3.28 and was characterised by high absolute loadings for plant aspect, (PASP; 0.47), grain yield (GY; -0.43), ears per plant (EPP; -0.39), and husk cover, and fall armyworm (HC; 0.30). The PC2 explained 17% of the variation with an eigenvalue of 2.49 and was characterised by ear aspect (EASP; -0.50), ear rot (EROT; -0.46), and southern corn leaf blight (SCLB; 0.32). The PC3 accounted for 12% of the variation with an eigenvalue of 1.75 and was characterised by SCLB (-0.49), HC (0.31), *Curvularia* leaf spot (CLS; -0.33), and foliar FAW damage at 8 weeks, and 4, 8, and 12 WAP (FFAWD4, FFAWD8, and FFAWD12; -0.31, -0.32, and -0.32, respectively). The PC4 explained 11% of the variation with an eigenvalue of 1.59 and was characterised by southern corn leaf rust (SCLR; -0.52), maize streak virus (MSV; 0.49), CLS (-0.37), and plant height (PH; 0.43). The PC5 explained 9% of the total variation with an eigenvalue of 1.28 and was characterised by CLS (0.40), MSV (0.39), GY (-0.36), ear height (EH; -0.34), and FFAWD4 (-0.33).

The PCA biplot is illustrated in Fig 2. Along PC1, PASP, HC, MSV, FFAWD4, FFAWD8, and FFAWD12 were positioned on the positive side of the axis, whereas GY, EPP, PH, EH, EASP, EROT, SCLR, SCLB, and CLS were positioned on the negative loadings for grain yield, plant height, and ears per plant. PC2 (16%) showed side. Along PC2, PH, EH, PASP, EASP, EROT, FFAWD4, FFAWD8, and FFAWD12 were positioned on the positive contributions from ear aspect, ear rot, and fall armyworm damage, butside, whereas GY, HC,

MSV, SCLR, SCLB, CLS, and EPP were positioned on the negative ~~loadings for maize streak virus, southern corn leaf blight, and rust. PC3 (12%) was dominated by southern corn leaf blight, curvularia leaf spot, and ear height, with maize streak virus~~side. Genotypes G5, G7, G16, G17, and G24 were positioned on the positive side of PC1, whereas G1, G8, G9, G18, and G22 were located on the negative~~. PC4 (9%) and PC5 (8%) highlighted curvularia leaf spot, husk cover, and rust.~~ side. Genotypes G13, G14, and G23 were positioned in the positive PC2 quadrant, while G3, G25, G26, and G29 were located in the negative PC2 quadrant.

Table 5. Principal component analysis of the contributions of grain yield, ~~foliar diseases~~other agronomic traits, foliar diseases, and foliar FAW damage to total variation among the maize hybrids and checks evaluated across environments.

	**Eigenvectors**				
**Traits**	**PC1**	**PC2**	**PC3**	**PC4**	**PC5**
**Grain yield (kg ha^{-1})**	**-0.43**	0.03	-0.02	0.04	**-0.36**
**Plant height (cm)**	-0.05	0.17	**-0.38**	**0.43**	0.00
**Ear height (cm)**	0.12	0.20	0.22	-0.22	**-0.34**
**Husk cover (1–5)**	**0.30**	0.19	**0.31**	-0.09	-0.10
**Plant aspect (1–5)**	**0.47**	0.06	0.05	-0.11	-0.06
**Ears per plant (count)**	**-0.39**	-0.22	-0.15	-0.19	-0.22
**Ear aspect (1–5)**	0.03	**-0.50**	-0.04	-0.11	0.25
**Ear rot (1–5)**	-0.21	**-0.46**	0.07	0.01	-0.06
**Maize streak virus (1–5)**	-0.11	0.23	0.05	**0.49**	**0.39**
**Southern corn leaf rust (1–5)**	-0.15	0.23	-0.16	**-0.52**	0.06
**Southern corn leaf blight (1–5)**	0.00	**0.32**	**-0.49**	-0.03	-0.22
***Curvularia* leaf spot (1–5)**	-0.11	0.22	**-0.33**	**-0.37**	**0.40**
**Foliar FAW damage at 4 WAP (1–9)**	0.26	-0.20	**-0.31**	0.10	**-0.33**
**Foliar FAW damage at 8 WAP (1–9)**	**0.32**	-0.26	**-0.32**	-0.14	0.29
**Foliar FAW damage at 12 WAP (1–9)**	0.27	-0.14	**-0.32**	0.13	-0.27
**Eigenvalue**	3.28	2.49	1.75	1.59	1.28
**Proportion (%)**	22	17	12	11	9
**Cumulative Proportion (%)**	22	38	50	61	69

PC = principal component; bolded values (|loading| ≥ 0.30) contributed more to the variation.

Fig 2. The PCA biplot showing the relationship between PC1 and PC2 for contributions of grain yield, foliar diseases, and other agronomic traits to total variation among the maize hybrids and checks evaluated across environments. GY = grain yield; PH = plant height; EH = ear height; HC = husk cover; PASP = plant aspect; EPP = ears per plant; EASP = ear aspect; EROT = ear rot; MSV = maize streak virus; SCLR = southern corn leaf rust; SCLB = southern corn leaf blight; CLS = *curvularia* leaf spot; FFAWD4, FFAWD8, FFAWD12 = foliar FAW damage ratings at 4, 8, and 12 WAP; G1-G32 = maize hybrids as presented in Table 1

Formatted: Font: Not Bold

-	Eigenvectors				
**Traits**	**PC1**	**PC2**	**PC3**	**PC4**	**PC5**
**Grain yield**	**-0.42**	-0.04	-0.01	**-0.29**	-0.09
**Plant height (cm)**	**-0.37**	0.13	0.22	-0.21	0.07
**Ear height (cm)**	**-0.31**	0.00	**0.31**	**-0.28**	0.22
**Husk cover (1-5)**	0.24	-0.17	0.02	**-0.34**	**0.38**
**Plant aspect (1-5)**	**0.41**	-0.01	0.15	-0.06	0.20
**Ears per plant (count)**	**-0.36**	0.20	0.04	0.14	-0.12
**Ear aspect (1-5)**	-0.01	**0.51**	0.02	0.27	0.00
**Ear rot (1-5)**	-0.21	**0.45**	-0.16	0.05	0.11
**Maize streak virus (1-5)**	-0.02	**-0.26**	**-0.36**	0.13	**-0.47**
**Southern corn leaf rust (1-5)**	-0.16	**-0.25**	**0.33**	**0.26**	**0.25**
**Southern corn leaf blight (1-5)**	-0.02	**-0.27**	**0.44**	-0.06	**-0.50**
**Curvularia leaf spot (1-5)**	-0.12	-0.21	**0.36**	**0.51**	0.09
**FAW damage at 4 weeks (1-9)**	0.18	**0.27**	**0.31**	**-0.32**	**-0.42**
**FAW damage at 8 weeks (1-9)**	**0.27**	**0.29**	**0.29**	**0.30**	-0.08
**FAW damage at 12 weeks (1-9)**	0.22	0.21	0.24	-0.17	-0.07
**Eigenvalue**	1.97	1.55	1.36	1.16	1.13
**Proportion (%)**	26	16	12	9	8
**Cumulative**	26	42	54	63	71

PC = principal component, Bolded values contributed higher to variation.

~~The biplot is visualized in Fig 2, showing clustering of related traits, such as fall armyworm damage at different weeks, indicating consistent behaviour across genotypes. Grain yield was positioned opposite to pest damage traits, reaffirming their negative correlation. Hybrids like G1, G8, and G9 clustered near the grain yield vector, identifying them as high-performing genotypes with favourable agronomic and resistance traits.~~

~~**Figure 2. PCA biplot illustrating the relationship between PC1 and PC2 for contributions of grain yield, foliar diseases and other agronomic traits to total variation among the maize hybrids and checks evaluated across environments.**~~

Correlation Analysis

Formatted: English (United States)

~~The strength and direction of linear relationships between traits showed that grain yield had a strong~~ Several significant positive ~~correlation with plant height ($r = 0.60$, $p < 0.001$) and moderate positive~~ and negative Spearman's correlations ~~with ear height ($r = 0.50$, $p < 0.01$) and ears per plant ($r = 0.53$, $p < 0.01$)~~were identified among the traits evaluated across environments (Fig~~.~~ 3). ~~Plant height correlated strongly with ear height ($r = 0.68$, $p < 0.001$) and moderately negatively~~Grain yield had a strong negative correlation coefficient with plant aspect (~~$r = -0.49$, $p < 0.01$). Ears per plant correlated positively with ear rot ($r = 0.51$, $p < 0.01$) but negatively with husk cover ($r = -0.52$, $p < 0.01$) and plant aspect ($r = -0.48$, $p < 0.01$). Grain yield was strongly and negatively associated with plant aspect ($r = -0.73$, $p < 0.001$) and moderately with fall armyworm damage at 8 weeks ($r = -0.49$, $p < 0.01$). Husk cover showed~~$\rho = -0.71$, $p < 0.001$), a strong positive correlation ~~with plant aspect ($r = 0.42$, $p < 0.05$), while ear aspect correlated positively~~coefficient with ears per plant ($\rho = 0.60$, $p < 0.001$), a moderate positive correlation coefficient with plant height ($\rho = 0.40$, $p < 0.05$) and ear height ($\rho = 0.38$, $p < 0.05$), and a moderate negative correlation coefficient with husk cover ($\rho = -0.38$, $p < 0.05$) and foliar fall armyworm damage at 8 WAP ($\rho = -0.43$, $p < 0.05$). Husk cover had a moderate

negative correlation coefficient with ears per plant (ρ = -0.47, p < 0.01) but a moderate positive correlation coefficient with plant aspect (ρ = 0.38, p < 0.05). Ears per plant had a moderate positive correlation coefficient with ear rot (ρ = 0.43, p < 0.05) and a moderate negative correlation coefficient with plant aspect (ρ = -0.44, p < 0.05).

Ear aspect had a moderate positive correlation coefficient with ear rot (~~r~~ρ = 0.50, p < 0.01) and a moderate positive correlation coefficient with foliar fall armyworm damage at 8 ~~weeks (r~~WAP (ρ = 0.~~47~~45, p < 0.~~01). Fall~~05). Foliar fall armyworm damage at 4 ~~weeks correlated positively~~WAP had moderate positive correlation coefficients with damage at 8 ~~weeks (r~~WAP (ρ = 0.~~46~~45, p < 0.~~01~~05) and 12 ~~weeks (r~~WAP (ρ = 0.~~34~~36, p < 0.05), while foliar fall armyworm damage at ~~12 weeks correlated negatively~~8 WAP had a moderate positive correlation coefficient with ~~maize~~damage at 12 WAP (ρ = 0.40, p < 0.05). Plant aspect had moderate positive correlation coefficients with foliar fall armyworm damage at 8 WAP (ρ = 0.37, p < 0.05) and 12 WAP (ρ = 0.36, p < 0.05), and a moderate negative correlation coefficient with plant height (ρ = -0.44, p < 0.05). Plant height had a moderate positive correlation coefficient with ear height (ρ = 0.38, p < 0.05). Maize streak virus ~~(r~~had a moderate negative correlation coefficient with foliar fall armyworm damage at 12 WAP (ρ = -0.~~35~~40, p < 0.05).

Fig 3. Correlogram showing the relationship between traits of ~~fall armyworm~~FAW-resistant maize hybrids and checks evaluated across environments. The cell value denotes correlation coefficient (~~r~~ρ) values~~.~~; *, **, *** = Significance at 0.05, 0.01, and 0.001 probability levels respectively; ns = non-significance; **GY = grain yield** ~~(kg/ha),~~, **PH = plant height** ~~(cm),~~, **EH = ear height** ~~(cm).~~, **HC = husk cover** ~~(1-5),~~, **PASP = plant aspect** ~~(1-5),~~, **EPP = ears per plant** ~~(count),~~, **EASP = ear aspect** ~~(1-5),~~, **EROT = ear rot** ~~(1-5),~~, **MSV = maize streak virus** ~~(1-5),~~, **SCLR = southern corn leaf rust** ~~(1-5),~~, **SCLB = southern corn leaf blight** ~~(1-5),~~, **CLS =** ***curvularia*** **leaf spot** ~~(1-5), FAWD4 = fall armyworm~~, FFAWD4 = foliar FAW **damage**

Formatted: Indent: First line: 0.5"
Formatted: Normal (Web), Line spacing: Double
Formatted: Font: Not Bold
Formatted: footnote Char, Font: Not Bold
Formatted: footnote Char, Font: Not Bold
Formatted: footnote Char, Font: Not Bold
Formatted: footnote Char, Font: Not Bold
Formatted: footnote Char, Font: Not Bold
Formatted: footnote Char, Font: Not Bold
Formatted: footnote Char, Font: Not Bold
Formatted: footnote Char, Font: Not Bold
Formatted: footnote Char, Font: Not Bold
Formatted: footnote Char, Font: Not Bold
Formatted: footnote Char, Font: Not Bold
Formatted: footnote Char, Font: Not Bold
Formatted: footnote Char, Font: Not Bold
Formatted: footnote Char, Font: Not Bold, Italic
Formatted: footnote Char, Font: Not Bold
Formatted: footnote Char, Font: Not Bold

at 4 ~~weeks (1-9), FAWD8 = fall armyworm~~WAP, FFAWD8 = foliar FAW **damage at 8** ~~weeks (1-9), FAWD12 = fall armyworm~~WAP, FFAWD12 = foliar FAW **damage at 12** ~~weeks (1-9).~~WAP.

Formatted: footnote Char, Font: Not Bold

Formatted: footnote Char, Font: Not Bold

Genotype by yield~~*~~ × trait analysis and superiority index

Formatted: English (United States)

Formatted: English (United States)

The genotype by yield~~*~~ × trait (GYT) analysis ~~integrated~~combined grain yield with ~~agronomic~~the traits ~~to rank maize hybrids based on performance across productivity and pest resistance traits. Standardized GYT values (Table 6) and the visualized GYT biplot (Fig 4) assessed yield and traits~~that had a significant hybrid effect, including ~~ear height,~~ plant aspect, ~~husk cover, and~~ear aspect, southern corn leaf blight, *curvularia* leaf spot, and foliar fall armyworm damage at 8 and 12 ~~weeks. Five~~WAP to rank the hybrids. The standardized genotype by yield × trait combinations and the corresponding superiority index are presented in Table 4.10. The GYT biplot explained 87.10% of the total variation among the yield × trait combinations, with PC1 accounting for 77.76% and PC2 accounting for 9.34% (Fig. 4). For clarity, entry numbers corresponding to each hybrid were used for ease of graphical presentation. Accordingly, G1 aligned with GY/PASP, GY/EASP, and GY/FFAWD8, G22 aligned with GY/FFAWD12, and G6 aligned with GY/SCLB, while the check Oba Super 11 (G28) aligned with GY/CLS. Based on the "which won where" analysis (Fig. 5), the perpendicular lines divided the polygon into eight sectors, and the yield × trait combinations were captured in sectors with vertex entries G1 (GY/PASP, GY/EASP, and GY/FFAWD8), G22 (GY/FFAWD12), G6 (GY/SCLB), and the check G28 (GY/CLS). The Average Tester Coordination (ATC) view (Fig. 6) identified the top five hybrids ~~emerged~~ as ~~top performers based on superiority indices (SI):~~ FAWSYN-1/(TZLComp. 1 C6-W-39-1-1)-B-B (G1), ~~Oba Super 11 (G28),~~ FAWSYN-1/IITATZI2305 (G22), FAWSYN-2/(TZLComp. 1 C6-W-39-1-1)-B-B (G9), FAWSYN-1/~~IITATZI2305 (G22), and FAWSYN-1/~~TZISTR1121 (G4~~). G1 achieved~~

Formatted: Indent: First line: 0.5"

the highest SI of 2.32, showing superiority across yield traits. The), and FAWSYN-3/IITATZI2305 (G19), whereas the bottom hybrids:five were FAWSYN-2 (G25), FAWSYN-3/TZISTR1872 (G16), FAWSYN-1/TZISTR1129 (G5), FAWSYN-1/TZISTR1305 (G7), FAWSYN-2/TZISTR1305 (G13), and SC301 (G29) underperformed in yield trait combinationsFAWSYN-1/TZISTR1129 (G5). Among the checks, Oba Super 11 (G28) had the highest superiority index.

Table 6: Standardized genotype by yield* × trait and superiority index of FAWRFAW-resistant maize hybrids evaluated across environments in 2024.

Hybrid	Pedigree	GY*EH/P ASP	GY/PASP EASP	GY/EASP SCLB	GY/SCLB CLS	GY/FAWD8F FAWD8	GY/FAWD12FF AWD12	SISuperiority Index	
G1	FAWSYN-1/(TZLComp. 1 C6-W-39-1-1)-B-B	2.8738	2.3856	2.561.87	1.8719	3.01	1.22	2.3204	
G28G22	Oba Super 11FAWSYN-1/IITATZI2305	1.3128	1.9399	1.51-0.47	2.310.98	1.182.72	2.011.51	1.8816	
G9	FAWSYN-2/(TZLComp. 1 C6-W-39-1-1)-B-B	2.281.66	1.6655	1.550.97	0.9784	0.28	1.01	1.2905	
G22	FAWSYN-1/IITATZI2305		0.56	1.28	1.99	-0.47	1.18	2.01	1.09
**G4**	**FAWSYN-1/TZISTR1121**	**0.341.41**	**1.410.97**	**0.9768**	**0.6840**	**0.96**	**1.37**	**0.9596**	
G19	FAWSYN-3/IITATZI2305	1.130.76	0.761.09	1.090.78	0.7839	0.80	0.43	0.8371	
G8G18	FAWSYN-1/TZISTR20423/IITATZI2300	0.9568	0.9560	-0.0138	0.5712	0.7297	1.7536	0.8268	
G18	FAWSYN-3/IITATZI2300		0.38	0.68	0.60	0.38	0.97	1.36	0.73
**G20G8**	**FAWSYN-1/IITATZI2300TZISTR2042**	**0.0695**	**-0.6301**	**0.7057**	**-0.3914**	**0.3572**	**0.591.75**	**0.4564**	
G6	FAWSYN-1/TZISTR2024	-0.5117	-0.1754	-0.542.69	2.69-0.05	-0.46	0.91	0.3845	
G20	FAWSYN-1/IITATZI2300	0.63	0.70	0.39	-0.12	0.35	0.59	0.42	
G21	FAWSYN-2/IITATZI2300	0.0224	0.2432	0.3212	0.1220	0.38	0.63	0.2932	
G31	Oba Super 9		0.98	0.41	0.52	0.00	0.13	-0.38	0.28
**G15**	**FAWSYN-3/TZISTR1869**	**-0.2627**	**-0.2710**	**-0.1013**	**-0.1326**	**-0.32**	**-0.07**	**-0.11**	
G10	FAWSYN-2/TZISTR1878	0.0133	-0.3359	-0.5962	-0.6200	-0.12	-0.01	-0.17	
G27G11	SAMMAZ 51FAWSYN-2/TZISTR1129	-0.1407	-0.1335	-0.1530	-0.4410	-0.6536	-0.0162	-0.2118	
G14	FAWSYN-2/TZISTR2129-2	-0.1641	-0.4112	-0.1295	-0.9526	0.12	0.27	-0.2123	
G26	FAWSYN-3	-0.1345	-0.4590	-0.9078	-0.7849	-0.51	-0.13	-0.2228	
G30	Oba Super 7		0.22	-0.34	-0.07	-0.44	0.12	-0.89	-0.23
**G11G23**	**FAWSYN-2/TZISTR1129IITATZI2305**	**-0.7119**	**-0.0745**	**-0.3595**	**-0.3031**	**-0.3608**	**-0.6278**	**-0.2831**	
G12	FAWSYN-2/TZISTR2024	-0.2973	-0.7367	-0.6751	-0.5135	-0.38	-0.35	-0.3233	
G3	FAWSYN-1/TZISTR1878	-0.3712	-0.1223	-0.2361	-0.6106	-0.59	-0.85	-0.3441	
G23	FAWSYN-2/IITATZI2305		-0.57	-0.19	0.45	-0.95	-0.08	-0.78	-0.35
**G2**	**FAWSYN-1/TZISTR1869**	**-0.4151**	**-0.5169**	**-0.6927**	**-0.2746**	**-0.33**	**-0.28**	**-0.4142**	
G17	FAWSYN-3/TZISTR1878	-0.2166	-0.6668	-0.681.26	-1.260.53	-0.98	0.07	-0.6267	
G32	Oba Super II		-1.11	-0.93	-0.77	0.51	-0.88	-0.69	-0.64
**G24**	**FAWSYN-1**	**-1.0612**	**-1.120.58**	**-0.5836**	**-0.3688**	**-0.45**	**-1.08**	**-0.7875**	
G13	FAWSYN-2/TZISTR1305		-0.85	-1.16	-0.53	-1.20	-0.49	-0.84	-0.85

**G5**	**FAWSYN-1/TZISTR1129**	**-~~1.02~~0.98**	**-0.~~98~~65**	**-0.~~65~~42**	**-0.~~42~~32**	**-0.88**	**-1.24**	**-0.~~87~~75**
~~**G29**~~**G13**	~~SC301~~FAWSYN-2/TZISTR1305	-~~0.90~~1.16	-0.~~55~~53	-1.~~46~~20	-~~1.04~~0.93	-~~1.16~~0.49	-0.~~88~~84	-~~1.00~~0.86
**G7**	FAWSYN-1/TZISTR1305	-~~0.94~~1.53	-~~1.53~~0.98	-~~0.98~~1.36	-1.~~36~~03	-0.47	-0.86	-1.~~02~~04
**G16**	FAWSYN-3/TZISTR1872	-1.~~38~~33	-1.~~33~~09	-~~1.09~~0.75	-~~0.75~~1.17	-1.42	-1.27	-1.~~21~~17
**G25**	FAWSYN-2	-1.~~63~~36	-1.~~36~~80	-~~1.80~~0.99	-~~0.99~~1.16	-1.23	-1.91	-1.~~49~~41
**Checks**								
**G28**	Oba Super 11	1.93	1.51	2.31	4.45	2.72	1.51	2.41
**G31**	Oba Super 9	0.41	0.52	0.00	0.37	0.13	-0.38	0.18
**G27**	SAMMAZ 51	-0.13	-0.15	-0.44	-0.34	-0.65	-0.01	-0.29
**G30**	Oba Super 7	-0.34	-0.07	-0.44	-0.21	0.12	-0.89	-0.31
**G32**	Oba Super II	-0.93	-0.77	0.51	0.46	-0.88	-0.69	-0.38
**G29**	SC301	-0.55	-1.46	-1.04	-0.73	-1.16	-0.88	-0.97
**Mean**		0.00	0.00	0.00	0.00	0.00	0.00	
**Standard deviation**		1.00	1.00	1.00	1.00	1.00	1.00	

GY~~:~~ = grain yield~~;~~, DP~~:~~ = days to pollination~~;~~, DS~~:~~ = days to silking~~;~~, ASI~~:~~ = anthesis-silking interval~~;~~, EH~~:~~ = ear height~~;~~, HC~~:~~ = husk-~~ ~~cover~~;~~, PASP~~:~~ = plant aspect~~;~~, SCLB~~:~~ = southern corn leaf blight~~;~~, CLS~~:~~ = *curvularia* leaf spot~~; **FAWD8: fall armyworm**~~, FFAWD8, FFAWD12 = foliar FAW damage at 8 **week**

and 12 WAP.

Fig 4. The Average Tester Coordination viewGYT biplot showing the relationships among the yield-trait combination of the FAWR32 FAW-resistant maize hybrids and checks evaluated across environments. The biplot was based on the singular value decomposition of trait-standardized data (“Scaling=1, Centering=2”) and genotype-focused singular value partition (“SVP=2”). The trait codes are: YLD: in 2024. GY = grain yield (kg/ha), **EH: ear height (cm);**, PASP: = plant aspect (1-5);, EASP: **ear aspect (1-5); FAWD8: fall armyworm damage at 8 weeks (1-9); FAWD12: fall armyworm damage at 12 weeks (1-9);** = ear aspect, SCLB: = southern corn leaf blight (1-5)., CLS = *curvularia* leaf spot, FFAWD8, FFAWD12 = foliar FAW damage at 8 and 12 WAP, G1-G32 = maize hybrids as presented in Table 1.

Fig 5. Which-won-where view of the GYT biplot for the 32 FAW-resistant maize hybrids and checks evaluated across environments in 2024. GY = grain yield, PASP = plant aspect, EASP = ear aspect, SCLB = southern corn leaf blight, CLS = *curvularia* leaf spot, FFAWD8, FFAWD12 = foliar FAW damage at 8 and 12 WAP, G1-G32 = maize hybrids as presented in Table 1.

Fig 6. The Average Tester Coordination view of the genotype by yield × trait (GYT) biplot for the 32 FAW-resistant maize hybrids and checks evaluated across environments in 2024. GY = grain yield, PASP = plant aspect, EASP = ear aspect, SCLB = southern corn leaf blight, CLS = *curvularia* leaf spot, FFAWD8, FFAWD12 = foliar FAW damage at 8 and 12 WAP, G1-G32 = maize hybrids as presented in Table 1.

Formatted: Font: Not Bold

Formatted: Font: Not Bold

Formatted: PCA TABLE Char, Font: Not Bold

Formatted: PCA TABLE Char, Font: Not Bold

Formatted: PCA TABLE Char, Font: Not Bold

Formatted: PCA TABLE Char, Font: Not Bold

Formatted: PCA TABLE Char, Font: Not Bold

Discussion

Formatted: English (United States)

The 32 FAWR maize hybrids evaluated in this study performed differently in grain yield, ear height, plant aspect, ear aspect, southern corn leaf blight, and fall armyworm damage at 8 and 12 weeks across environments. Maize hybrids and open-pollinated varieties are more vulnerable to FAW damage at early growth stages but can recover in later stages, while inbred lines show increasing damage over time (19). Traits such as husk cover, ear rot resistance, and plant height correlate with FAW tolerance (20).

The significant hybrid differences observed in traits such as grain yield, ear height, plant aspect, and ear aspect underscore the importance of these characteristics in maize breeding. Specifically, ear aspect has been consistently identified as a major trait contributing directly to grain yield, alongside ears per plant, which also impacts yield positively (21–23). In contrast, the absence of significant differences in traits like plant height, husk cover, and ears per plant among fall armyworm resistance hybrids suggests that these traits may not exhibit the variability necessary for selection, indicating a potential limitation in trait diversity for breeding purposes (24,25). This aligns with findings that show positive associations between yield and certain traits, while others do not exhibit such relationships (26,27).

Variance component analysis revealed that environmental factors play a dominant role in trait expression, as indicated by the consistently higher phenotypic compared to genotypic variance across most traits (28). A broad-sense heritability of 0.44 for maize grain yield classified as moderate (29) suggests that approximately 44% of the variation in maize grain yield is due to genetic differences among the plants, and the remaining 56% is influenced by environmental factors (like rainfall, soil fertility, temperature) and other non-genetic factors (30). For most other traits, low heritability estimates highlight the challenge of achieving genetic gains through conventional selection alone, emphasizing the need for multi-location testing and the integration of genomic selection approaches.

Principal component analysis (PCA) further clarified the relationships among traits, with the first five components explaining a substantial 71% proportion of total variation. The negative association between grain yield and pest damage traits in PC1 reflects the classic trade-off between productivity and stress tolerance, reinforcing the challenge of breeding for both high yield and resilience (18).

Correlation analysis revealed that key yield components, such as plant height and ears per plant, are positively associated with grain yield (31), while negative correlations between yield and both plant aspect and FAW damage highlight the detrimental effects of poor plant architecture and pest pressure (32). These relationships underscore the importance of multi-trait selection indices that can balance yield improvement with enhanced resistance and optimal plant type (33).

The genotype by yield*trait (GYT) analysis provided a holistic ranking of hybrids, integrating yield with agronomic and resistance traits (18). The identification of superior hybrids such as G1 and G28 demonstrates the success of both experimental and commercial breeding efforts in delivering genotypes with balanced performance.

Insufficient maize production in many regions is largely driven by FAW outbreaks, which could reduce maize yields and cause annual losses that jeopardize the livelihoods of smallholder farmers (11). Cultivating resistant hybrids could reduce insecticide dependence, lower production costs, and contribute to sustainable food security in FAW-affected regions (22). Thirty-two FAW-resistant maize hybrids and checks were evaluated across two environments exposed to natural FAW pressure to determine resistance levels and yield potential. Rainfall was highly irregular, and FAW pressure varied but remained sufficient at both experimental sites, allowing differentiation of hybrid responses.

The significant hybrid effect, along with hybrid × environment (G × E) and environment effects for several traits, indicated genetic variation whose expression depended on growing conditions. Similar patterns were reported in FAW-resistance studies in which genotype and G × E effects were significant for grain yield and FAW leaf and ear damage under artificial or natural infestation (33–35). The significant environmental effects observed further indicated that environmental conditions strongly influenced both productivity and resistance expression (22,33,34). The high R^2 values were consistent with reports of moderate-to-high heritability for FAW foliar damage and grain yield, which suggested that selection for resistance and agronomic performance could be effective when trials were appropriately replicated across environment (22,36). The coefficients of variation above 20% for grain yield and foliar FAW damage were consistent with FAW screening trials and likely reflected heterogeneous pest pressure and complex FAW damage–yield relationships (33–35).

Grain yield levels were lower than those reported in FAW-tolerant hybrid trials conducted under on-station, artificial infestation, or well-managed conditions, where tolerant hybrids frequently produced 4.6–7.1 t/ha, and some entries exceeded 7 t/ha under FAW pressure (22,36,37). However, comparable or lower yields were reported under natural infestation and smallholder-type stress conditions, such as in Mozambique, where the mean yield was approximately 0.8 t/ha, and the best entry reached about 1.3 t/ha (38). This contrast suggested that yield performance was strongly influenced by the intensity of environmental stress, particularly irregular rainfall, agronomic management, and pest pressure (22,36). Foliar FAW damage levels were consistent with ranges reported in hybrid and open-pollinated maize hybrids evaluated under artificial infestation, where damage scores commonly ranged from moderate to moderately high depending on genotype and infestation timing (33,35,37). Similar studies reported that hybrids with lower foliar FAW damage tended to maintain higher grain yield, reflecting the negative relationship between foliar FAW damage and productivity

(33,39). Ear aspect, husk cover, and ear rot responses aligned with reports describing FAW-tolerant hybrids as possessing acceptable ear quality, improved husk protection, and reduced secondary ear damage, thereby helping maintain yield under infestation (22,37,39). Comparable performance of commercial checks relative to trial entries was also reported in FAW screening studies, in which checks frequently fell within the overall range. Still, it was often surpassed by improved FAW-resistant hybrids (22,34,35).

The negligible or zero genotypic variance for most agronomic and disease traits and for several foliar FAW damage scores indicated that environmental factors played a dominant role in trait expression (40). This could be due to spatially heterogeneous factors or environmental variability, such as soil fertility or moisture, which may also have been absorbed into the residual term, thereby lowering the estimated genotypic variance. This is in contrast with other multi-environment studies that reported non-zero genotypic variance and moderate genotypic coefficients of variation for grain yield and foliar FAW damage traits (26,33,34). In contrast, some FAW studies that applied mixed-model approaches, including spatial or related adjustments, often reduced residual variance and recovered larger genotypic variance components (29,41). Genotype composition may also have contributed; closely related elite maize hybrids could have expressed genuinely smaller among-genotype variance for several traits than more diverse sets that included broad germplasm sources, landraces, or open-pollinated varieties (33,34).

The first two principal components were primarily associated with agronomic and ear-quality traits, whereas foliar FAW damage scores contributed more strongly to later components. This differed from other FAW studies, in which early principal components were dominated by foliar FAW-damage variables and their association with grain yield (36,42). This could be due to the inclusion of broader agronomic traits, consistent with studies that predominantly focus on plant architecture and yield-related variables, such as plant height, ear

traits, and grain yield (43–45). This pattern suggested that variation among maize hybrids was more strongly explained by plant morphology, yield components, and disease-related traits than by foliar FAW damage scores alone.

The Spearman's correlation pattern indicated that grain yield was closely associated with plant architecture and key yield components, consistent with FAW-infested multi-environment trials in which ears per plant, plant height, ear height, and ear-related traits were major contributors to yield variation under stress conditions (34,36,38). Negative associations between foliar FAW damage and grain yield were also consistent with findings from Mozambique, Nepal, and other FAW-affected environments, where foliar and ear damage reduced grain yield and ear development. However, the magnitude of these relationships depended on genotype and infestation intensity (46,47). Similar relationships were reported under both controlled infestation and field conditions, indicating that foliar FAW damage reduced productivity primarily through its effects on plant vigor and ear appeal (22,48,49). Consistent associations among foliar FAW damage scores across the growth stages and with agronomic traits were also reported, reflecting the tendency for susceptible maize hybrids to exhibit sustained injury and reduced agronomic performance. In contrast, tolerant hybrids maintained more favorable growth and yield characteristics under infestation (34,36).

Grain yield is a genetically complex trait influenced by multiple agronomic and environmental factors; therefore, direct selection for yield alone is often inefficient, particularly under variable stress conditions (50). In contrast, phenology, growth, and resistance-related traits often exhibited higher heritability and lower genotype × environment sensitivity, making them more reliable targets for indirect selection (51). Multi-trait indices such as GYT therefore provided a more robust basis for advancement because they ranked genotypes using yield–trait combinations that jointly captured productivity, stress tolerance, and overall plant type (31,52). The high ranking of the commercial check, OBA Super 11, on the GYT superiority index

indicated a favorable aggregate profile of grain yield, phenotypic appeal, foliar diseases, and foliar FAW damage, consistent with reports in which commercial checks or released cultivars occupied leading GYT positions because of broadly optimized trait packages (53–55). Advancing the highest-ranked experimental hybrids based on the GYT superiority index, therefore prioritized entries that combined yield with other traits with significant hybrid effects, supporting more efficient parent selection and cultivar advancement than selection based on grain yield alone (56,57).

Conclusions

Formatted: English (United States)

The results from this study emphasize the extent of variability among fall armyworm resistant maize hybrids, especially on grain yield, ear height, and plant and ear aspects. These differences offer valuable opportunities for breeders to select hybrids with strong overall performance. While some traits like ears per plant and ear aspect show clear connections to higher yield, others, such as plant height and husk cover, didn't vary as much among the hybrids, which may limit their inclusion in selection criteria. The moderate heritability of grain yield suggests that both genetics and the environment play major roles, indicating that selection efforts need to account for performance across different environments. Interestingly, the conflicts observed between yield and pest resistance reinforce the ongoing challenge of breeding for both productivity and resilience. The principal component analysis and GYT analysis clarified hybrids G1 and G28 performed excellently for their balance of agronomic strength and stress tolerance, showing that it is possible to breed maize that performs well without compromising on resilience. Going forward, breeding programs will benefit from combining traditional selection with genomic tools and multi-trait approaches to develop more adaptable and high-performing maize varieties.

Unpredictable rainfall patterns, including cessation of rainfall after planting and excessive rainfall after maize reached maturity, likely imposed moisture stress during early growth stages and increased lodging, thereby affecting yield evaluation. Despite these constraints, genetic variability was observed among the fall armyworm-resistant maize hybrids evaluated, and environmental conditions significantly influenced phenotypic expression, potentially affecting trait expression and hybrid rankings. A subset of hybrids nonetheless maintained comparatively higher grain yield across environments, indicating potential for selection under FAW pressure. Grain yield reductions associated with foliar FAW damage and unfavorable plant traits likely translated into economic losses for farmers. Cultivation of the identified fall armyworm-resistant maize hybrids could therefore improve profitability and support more sustainable production in the derived savanna agro-ecology. The top-performing topcross hybrids identified showed potential to reduce the impact of FAW damage; however, additional multi-environment testing and validation were recommended to confirm performance across a wider range of environments and management conditions and to support release to smallholder farmers through location-specific hybrid targeting.

Acknowledgement

We ~~appreciate~~thank the International Institute of Tropical Agriculture~~,~~ (IITA~~)~~), Ibadan, Nigeria, for providing the fall armyworm~~-~~-resistant maize hybrids used in this study.

References

Formatted: English (United States)

1. Erenstein O, Jaleta M, Sonder K, Mottaleb K, Prasanna BM. Global maize production, consumption and trade: trends and R&D implications. Food Secur. 2022 Oct;14(5):1295–319. doi:10.1007/s12571-022-01288-7

22. Olayinka AO. Field Performance of Extra-Early Maturing Pro-Vitamin A Maize (Zea mays L.) Hybrids under Optimal Rainfed Conditions in the Derived Savanna Agroecology Zone. Crop Breed Genet Genomics. 2025 Aug;7(3). doi:10.20900/cbgg20250012

3. Yang N, Yan J. New genomic approaches for enhancing maize genetic improvement. Curr Opin Plant Biol. 2021 Apr;60:101977. doi:10.1016/j.pbi.2020.11.002

3. Klein HS, Luna FV. Maize. In: Brazilian Crops in the Global Market [Internet]. Cham: Springer Nature Switzerland; 2023 [cited 2025 July 30]. p. 107–29. (Palgrave Studies in Economic History). Available from: https://link.springer.com/10.1007/978-3-031-38589-6_5

44. Klein HS, Luna FV. The Impact of the Rise of Modern Maize Production in Brazil and Argentina. Hist Agrar Rev Agric E Hist Rural. 2022 Apr 1. doi:10.26882/histagrar.086e09k

5. Badu-Apraku B, Fakorede MAB. Maize in Sub-Saharan Africa: Importance and Production Constraints. In: Advances in Genetic Enhancement of Early and Extra-Early Maize for Sub-Saharan Africa [Internet]. Cham: Springer International Publishing; 2017 [cited 2025 JulyJul 30]. p. 3–10. Available from: http://link.springer.com/10.1007/978-3-319-64852-1_1 doi:10.1007/978-3-319-64852-1_1

5. Statista. Statista. 2023 [cited 2025 Mar 2].6. Sasu. Nigeria: maizeMaize production quantity| Statista [Internet]. 2024. Available from: https://www.statista.com/statistics/1300743/production-volume-of-maize-in-nigeria/

67. Iseghohi I, Abe A, Meseka S, Mengesha W, Gedil M, Job A, et al. Reactions of provitamin-A-enriched maize to foliar diseases under field conditions in Nigeria. Cereal Res Commun. 2024 Jun 1;52(2):747–58. doi:10.1007/s42976-023-00395-1

8. Goergen G, Kumar PL, Sankung SB, Togola A, Tamò M. First Report of Outbreaks of the Fall Armyworm Spodoptera frugiperda (J E Smith) (Lepidoptera, Noctuidae), a New Alien Invasive Pest in West and Central Africa. Luthe DS, editor. PLOS ONE. 2016 Oct 27;11(10):e0165632. doi:10.1371/journal.pone.0165632

79. Iseghohi I, Ogundare E, Fayemiro S, Obisesan O, Job A. Assessment of maize hybrids for agronomic and fall armyworm tolerant traits. J Cent Eur Agric. 2025;26(4):927–38. doi:10.5513/JCEA01/26.4.4631

10. Day R, Abrahams P, Bateman M, Beale T, Clottey V, Cock M, et al. Fall Armyworm: Impacts and Implications for Africa. Outlooks Pest Manag. 2017 Oct 1;28(5):196–201. doi:10.1564/v28_oct_02

811. Togola A, Beyene Y, Bocco R, Tepa-Yotto G, Gowda M, Too A, et al. Fall armyworm (Spodoptera frugiperda) in Africa: Insights into biology, ecology and impact on staple crops, food systems and management approaches. Frontiers in Agronomy [Internet].

Frontiers Media SA; 2025 [cited 2026 Feb 9]. p. 1538198. Available from: https://www.frontiersin.org/journals/agronomy/articles/10.3389/fagro.2025.1538198/full

12. Abro Z, Kimathi E, De Groote H, Tefera T, Sevgan S, Niassy S, et al. Socioeconomic and health impacts of fall armyworm in Ethiopia. Nagoshi RN, editor. PLOS ONE. 2021 Nov 4;16(11):e0257736. doi:10.1371/journal.pone.0257736

~~9~~13. Utono IM, Adamu RS. Effect of belt expert (Flubendiamide + Thiacloprid), Imidacloprid, Thiamethoxam seed treatment and economic impact on fall armyworm (*Spodoptera frugiperda*) infestation on Maize in Nigeria. Cogent Food Agric. 2023 Dec 31;9(1):2164117. doi:10.1080/23311932.2022.2164117

~~10~~14. Tambo JA, Kansiime MK, Mugambi I, Agboyi LK, Beseh PK, Day R. Economic impacts and management of fall armyworm (Spodoptera frugiperda) in smallholder agriculture: a panel data analysis for Ghana. CABI Agric Biosci. 2023 Oct 2;4(1):38. doi:10.1186/s43170-023-00181-3

~~11~~15. Tambo JA, Liverpool-Tasie LSO. Are farm input subsidies a disincentive for integrated pest management adoption? Evidence from Zambia. J Agric Econ. 2024 Jun;75(2):740–63. doi:10.1111/1477-9552.12582

16. Kenyi JJ, Mengesha W, Abe A, Menkir A, Meseka S. Variation in grain yield losses due to fall armyworm infestation among elite open-pollinated maize varieties under different levels of insecticide application. Agriculture. 2024;14(7):984. doi:10.3390/agriculture14070984

17. Matova PM, Kamutando CN, Warburton ML, Williams WP, Magorokosho C, Shimelis H, et al. New techniques for breeding maize (*Zea mays*) varieties with fall armyworm resistance and market-preferred traits for sub-Saharan Africa. Plant Breed. 2023 Feb;142(1):1–11. doi:10.1111/pbr.13063

18. Woolfolk SW, Jeffers D, Hawkins LK, Uhdre R, Ni X, Warburton ML. Integrated approaches to maximizing maize resistance to fall armyworm. CABI Rev. 2025 Feb 20;0013. doi:10.1079/cabireviews.2025.0013

19. Krishna VV, Lantican MA, Prasanna BM, Pixley K, Abdoulaye T, Menkir A, et al. Impact of CGIAR maize germplasm in Sub-Saharan Africa. Field Crops Res. 2023 Jan;290:108756. doi:10.1016/j.fcr.2022.108756

~~12~~20. Adunola MP, Fayeun LS, Fadara AB. Impact of climate change on armyworm infestation on maize in Nigeria: A review. J Plant Breed Crop Sci. 2021 ~~Sept~~Sep 30;13(3):158–67. doi:10.5897/JPBCS2020.0895

~~13~~21. Asare S, Kena A, Amoah S, Annor B, Osekre EA, Akromah R. Screening of maize inbred lines and evaluation of hybrids for their resistance to fall armyworm. Plant Stress. 2023;8:100148. doi:10.2139/ssrn.4206925

22. Amegbor IK, Adu GB, Nelimor C, Prasanna BM, Beyene Y, Chivasa W, et al. Assessment of fall armyworm tolerant maize hybrids for sustainable maize production in sub-Saharan Africa. Phytoparasitica. 2025 Feb;53(2):29. doi:10.1007/s12600-025-01253-y PubMed PMID: 39974150.

23. Ni X, Huffaker A, Schmelz EA, Xu W, Williams WP, Guo B, et al. Field evaluation of experimental maize hybrids for resistance to the fall armyworm (Lepidoptera: Noctuidae) in a warm temperate climate. Insects. 2024;15(4):289. doi:10.3390/insects15040289

24. Kolawole AO, Olayinka AF. Phenotypic performance of new pro-vitamin A maize (Zea mays L.) hybrids using three selection indices. Agric Pol Nohospodárstvo. 2022 Apr;68(1):1–12. doi:10.2478/agri-2022-0001

25. Badu-Apraku B, Fakorede MAB, Menkir A, Sanogo D. Conduct and management of maize field trials. [Internet]. 2012 [cited 2025 JulyJul 30];]. Available from: https://cgspace.cgiar.org/bitstreams/81fda619-5d11-4b6f-9885-bf75ba268cc7/download

1426. Matova PM, Kamutando CN, Magorokosho C, Kutywayo D, Gutsa F, Labuschagne M. Fall-armyworm invasion, control practices and resistance breeding in Sub-Saharan Africa. Crop Sci. 2020 Nov;60(6):2951–70. doi:10.1002/csc2.20317

1527. Olivoto T, Lúcio AD. metan: An R package for multi-environment trial analysis. Jarman S, editor. Methods Ecol Evol. 2020 JuneJun;11(6):783–9. doi:10.1111/2041-210X.13384

1628. Abebe AT, Adewumi AS, Adebayo MA, Shaahu A, Mushoriwa H, Alabi T, et al. Genotype x environment interaction and yield stability of soybean (Glycine max l.) genotypes in multi-environment trials (METs) in Nigeria. Heliyon. 2024 Oct;10(19):e38097. doi:10.1016/j.heliyon.2024.e38097

29. Alvarado G, Rodríguez FM, Pacheco A, Burgueño J, Crossa J, Vargas M, et al. META-R: A software to analyze data from multi-environment plant breeding trials. Crop J. 2020;8(5):745–56. doi:10.1016/j.cj.2020.03.010

1730. Jolliffe IT, Cadima J. Principal component analysis: a review and recent developments. Philos Trans R Soc Math Phys Eng Sci. 2016 Apr 13;374(2065):20150202. doi:10.1098/rsta.2015.0202

1831. Yan W, Frégeau-Reid J. Genotype by yield* trait (GYT) biplot: a novel approach for genotype selection based on multiple traits. Sci Rep. 2018;8(1):8242.

19. Kenyi JJ, Mengesha W, Abe A, Menkir A, Meseka S. Variation in grain yield losses due to fall armyworm infestation among elite open-pollinated maize varieties under different levels of insecticide application. Agriculture. 2024;14(7):984.

20. Matova PM, Kamutando CN, Kutywayo D, Magorokosho C, Labuschagne M. Fall armyworm tolerance of maize parental lines, experimental hybrids, and commercial cultivars in Southern Africa. Agronomy. 2022;12(6):1463.

21. Badu-Apraku B, Magorokosho C, Fasanmade Y, Fakorede MAB, Talabi AO, Asiedu R, et al. Yield gains and associated changes in an early yellow bi-parental maize population following genomic selection for Striga resistance and drought tolerance. BMC Plant Biol. 2019 Apr 5;19(1).

22. Nasser LM, Badu-Apraku B, Gracen VE, Mafouasson HNA. Combining Ability of Early-Maturing Yellow Maize Inbreds under Combined Drought and Heat Stress and Well-Watered Environments.32. Gholizadeh A, Ghaffari M. Genotype by yield* trait

(GYT) biplot analysis: A novel approach for phenotyping sunflower single cross hybrids based on multiple traits. Food Sci Nutr. 2023 Oct;11(10):5928–37. doi:10.1002/fsn3.3524

33. Abubakar AM, Adejumobi II, Oyekunle M, Bonkoungou TO, Muhammad U, Udah O, et al. Agronomy. 2020 Oct 16;10(10):1585.

23. Amegbor IK, Van Biljon A, Shargie N, Tarekegne A, Labuschagne MT. Heritability and Associations among Grain Yield and Quality Traits in Quality Protein Maize (QPM) and Non-QPM Hybrids. Plants. 2022 Mar 8;11(6):713.

24. Darshan R, Prasanna P M, Hegde JN. Screening of Popular Maize Hybrids against fall for Fall Armyworm, ((SPODOPTERA FRUGIPERDA. J Exp Agric Int. 2024 Mar 21;46(5):306–12. . E. Smith) Resistance in Early-Maturing Tropical Maize Adapted to Sub-Saharan Africa. Plant Breed. 2025 Jun 4;pbr.13291. doi:10.1111/pbr.13291

34. Kamweru I, Beyene Y, Bruce AY, Makumbi D, Adetimirin VO, Pérez-Rodríguez P, et al. Genetic analyses of tropical maize lines under artificial infestation of fall armyworm and foliar diseases under optimum conditions. Front Plant Sci. 2023 Jan;14:1086757. doi:10.3389/fpls.2023.1086757 PubMed PMID: 36743507.

35. Job A, Iseghohi I, Abe A, Yahaya M, Olayiwola R, Akinwale R, et al. Genetic analysis of agronomic and fall armyworm-resistance traits in maize hybrids with varying levels of resistance to stem borers. Agronomy. 25. Sultana R. MOLECULAR AND MORPHOLOGICAL CHARACTERIZATION OF MAIZE GENOTYPES FOR OPAQUE 2 GENE AND YIELD CONTRIBUTING TRAITS. Big Data Agric. 2019 Jan 12;1(1):07–14.

26. Bhadmus OA, Badu-Apraku B, Adeyemo OA, Ogunkanmi AL. Genetic Analysis of Early White Quality Protein Maize Inbreds and Derived Hybrids under Low-Nitrogen and Combined Drought and Heat Stress Environments. Plants. 2021 Nov 26;10(12):2596.

27. Badu-Apraku B, Adu GB, Yacoubou AM, Toyinbo J, Adewale S. Gains in Genetic Enhancement of Early Maturing Maize Hybrids Developed during Three Breeding Periods under Striga-Infested and Striga-Free Environments. Agronomy. 2020 Aug 13;10(8):1188.

282022;12(12):3042. doi:10.3390/agronomy12123042

36. Wambi W, Makumbi D, Asea G, Zeleke H, Bruce A, Wakgari M, et al. Use of multi-trait principal component selection index to identify fall armyworm (Spodoptera frugiperda) resistant maize genotypes. Front Plant Sci. 2025 Mar 27;16. doi:10.3389/fpls.2025.1544010

37. Nesma AZM, Ayodeji A, Anthony OJ, Yinka OK, Amudalat BO. Evaluation of stem borer resistant maize genotypes for resistance to fall armyworm (Spodoptera frugiperda J.E. SMITH) infestation. J Plant Breed Crop Sci. 2023 Sep 30. doi:10.5897/jpbcs2023.1023

38. Mazive GA, Mubai NH, Magamba CP, Afo DIB, Boa AF. Grain yield and its relationship with yield components in selected maize (Zea mays L.) varieties under natural fall armyworm infestation in Mozambique. Contrib Las Cienc Soc. 2024 Dec;17(13). doi:10.55905/revconv.17n.13-228

Formatted: Small caps

Formatted: Font: Italic, Small caps

39. Kasoma C, Shimelis H, Laing M, Shayanowako A, Mathew I. Screening of inbred lines of tropical maize for resistance to fall armyworm, and for yield and yield-related traits. Crop Prot. 2020 Oct 1;136:105218. doi:10.1016/j.cropro.2020.105218

40. Alam M, Rahman M, Ahmed S, Jahan N, Khan M, Islam M, et al. Genetic Variation and Genotype by Environment Interaction for Agronomic Traits in Maize (Zea mays L.) Hybrids. Plants. 2022 ~~June~~Jun 6;11(11):1522. doi:10.3390/plants11111522

~~29. Johnson HW, Robinson HF, Comstock RE. Estimates of genetic and environmental variability in soybeans. 1955 [cited 2025 July 31]; Available from: https://www.cabidigitallibrary.org/doi/full/10.5555/19561600791~~

~~30. Khajoane TJ. Genotype and environmental effects on maize grain yield, nutritional value and milling quality. 2022 [cited 2025 July 31]; Available from: https://scholar.ufs.ac.za/items/8cbafbac-5082-46f7-946f-da3878877362~~

~~31. Aman J, Bantte K, Alamerew S, Sbhatu DB. Correlation and Path Coefficient Analysis of Yield and Yield Components of Quality Protein Maize (*Zea mays* L.) Hybrids at Jimma, Western Ethiopia. Int J Agron. 2020 June 8;2020:1–7.~~

~~32. Chisonga C, Chipabika G, Sohati PH, Harrison RD. Understanding the impact~~41. Argaw T, Fenta B, Zegeye H, Azmach G, Funga A. Multi-environment trials data analysis: linear mixed model-based approaches using spatial and factor analytic models. Front Res Metr Anal. 2025 Apr 11;10. doi:10.3389/frma.2025.1472282

42. Stelinski L, Gre N, Ni X, Huffaker A, Schmelz E, Xu W, et al. Field Evaluation of Experimental Maize Hybrids for Resistance to the Fall Armyworm (Lepidoptera: Noctuidae) in a Warm Temperate Climate. Insects. 2024 Apr 1;15. doi:10.3390/insects15040289

43. Talefe W. Cluster and principal component analysis of maize inbred lines in low moisture stress areas in Ethiopia. Int J Agric Sci Food Technol. 2023 Jul 6. doi:10.17352/2455-815x.000194

44. Carnimeo ESG, Bertasello LET, Dutra SMF, Môro GV. Principal component analysis for selection of superior maize genotypes. 55292020V48N4P357-362. 2020. doi:10.15361/1984-

45. Singh D, Swapnil, Kushwaha N, Mohanty TA, Kumar A, Kumar R, et al. Multivariate analysis for yield and its component traits in Quality Protein Maize (QPM). Int J Adv Biochem Res. 2024 Jan 1. doi:10.33545/26174693.2024.v8.i2i.807

46. Bhandari GS, Bhandari P, Sah L. Evaluation of maize genotypes for resistance to fall armyworm under natural infestation conditions. Nepal Agric Res J. 2025 Jun 25. doi:10.3126/narj.v16i1.80465

47. Gadekar M, Das S, Shukla A, Sharma S, Patnaik S, Kakade S. Quantification of Yield Losses in Maize Attributed to Fall Armyworm (Spodoptera frugiperda ~~JE~~) Infestation. J Sci Res Rep. 2025 Sep 18. doi:10.9734/jsrr/2025/v31i93516

48. Bakry M, Abdel-Baky N. Impact of the fall armyworm, Spodoptera frugiperda (Lepidoptera: Noctuidae) infestation on maize growth characteristics and yield loss. Braz J Biol Rev Brasleira Biol. 2023 Jul 21;84. doi:10.1590/1519-6984.274602

49. Anjorin F, Odeyemi OO, Adenike O, Kehinde A, Kareem T. Fall armyworm (Spodoptera frugiperda) (J. E. Smith) ~~leaf damage~~(Lepidoptera: Noctuidae) infestation: maize yield depression and physiological basis of tolerance. J Plant Prot Res. 2023 Mar 22. doi:10.24425/jppr.2022.140294

50. Fadhli N, Farid Muh, Azrai M, Nur A, Efendi R, Priyanto S, et al. Morphological parameters, heritability, yield component correlation, and multivariate analysis to determine secondary characters in selecting hybrid maize. Biodiversitas J Biol Divers. 2023 Jul 18. doi:10.13057/biodiv/d240712

51. Singamsetti A, Shahi J, Zaidi P, Seetharam K. Study on ~~maize yields. PLoS One. 2023;18(6):e0279138.~~ Applicability of Genotype × Yield × Trait (GYT) Biplots over Genotype × Trait (GT) Biplots in Selection of Maize Hybrids across Soil Moisture Regimes. Indian J Agric Res. 2022 Mar 4. doi:10.18805/ijare.a-5850

~~33. Moeinizade S, Kusmec A, Hu G, Wang L, Schnable PS. Multi-trait Genomic Selection Methods for Crop Improvement. Genetics. 2020 Aug 1;215(4):931–45.~~ 52. Hosseini SMS, Shiri M, Mostafavi K, Mohammadi A, Miri SM. Genetic analysis and association detection of agronomic traits in maize genotypes. Sci Rep. 2025 Jan 2;15. doi:10.1038/s41598-024-84471-4

53. Bakhshi B, Amiri Oghan H, Rameeh V, Zeinalzadeh Tabrizi H, Askari A, Faraji A, et al. Trait profiling and genotype selection in oilseed rape using genotype by trait and genotype by yield*trait approaches. Food Sci Nutr. 2023 Feb 28;11:3083–95. doi:10.1002/fsn3.3290

54. Shojaei SH, Mostafavi K, Khosroshahli M, Bihamta MR, Ramshini H. Assessment of genotype-trait interaction in maize (Zea mays L.) hybrids using GGT biplot analysis. Food Sci Nutr. 2020 Aug;8(10):5340–51. doi:10.1002/fsn3.1826 PubMed PMID: 33133537.

55. Yan W. Two types of biplots to integrate multi-trial and multi-trait information for genotype selection. Crop Sci. 2024 Mar 20. doi:10.1002/csc2.21231

56. Dang X, Lin Y, Hu X, Yanli L. Comprehensive evaluation of sugar beet varieties based on genotype × yield × trait (GYT) in different environments. Sci Rep. 2025 Mar 21;15. doi:10.1038/s41598-025-90126-9

57. Rad MRN, Bakhshi B, Ghasemi A, Ghasemi MM, Kohpalekani JA. Identification of High-Yielding and Drought-Tolerant Melon Genotypes Based on Yield–Trait Combinations: A Comparison of Pcor Index and GYT Biplot Approaches. Food Sci Nutr. 2025 Feb 28;13. doi:10.1002/fsn3.70048

Response to Reviewers

Dear Academic Editor and Reviewer(s),

We sincerely thank you for the evaluation and constructive comments provided on our manuscript titled: Assessment of agronomic traits in fall armyworm (*Spodoptera frugiperda*) resistant maize (*Zea mays*) hybrids grown in the derived savanna agro-ecology of Nigeria.

We have revised the manuscript thoroughly as follows:

Comment 1: Please specify the type of fall armyworm damage evaluated (leaf, stalk, or ear damage).

Response: The study evaluated foliar fall armyworm damage (leaf damage) using the modified Davis 1–9 scale at 4, 8, and 12 weeks after planting. This has now been explicitly stated in the Abstract, Materials and Methods (Data Collection section), Results tables, and figure captions to ensure clarity and consistency throughout the manuscript.

Comment 2: The plant height values appear questionable for tropical hybrids. Please review and correct.

Response: Upon careful re-examination of the raw dataset and statistical outputs, we identified a unit-of-measurement handling error during statistical processing. Field measurements were correctly recorded in centimeters; however, an error occurred during data handling that affected the reported summaries. This error has now been corrected, and all affected tables and related results have been regenerated using the corrected dataset. The revised plant and ear height values are accurate and internally consistent.

Comment 3: The reported height of Oba Super II contradicts previous studies.

Response: Following correction of the unit-processing error described above, all genotype height values including Oba Super II were updated. The corrected values now reflect the properly processed field measurements for the 2024 growing season.

Comment 4: The ANOVA/mixed model description is not correct. The replicate effect is missing, and the nesting structure should reflect the alpha-lattice design.

Response: The Statistical Data Analysis section was revised to correctly reflect the alpha-lattice experimental design. Genotype and environment were treated as random effects. Replicates were modeled as nested within environment, and incomplete blocks were

modeled as nested within replicate and environment. The genotype × environment interaction was retained within the mixed-model framework. The model description has been rewritten accordingly for accuracy and transparency.

Comment 5: Avoid interpretation in the Results section. Move explanatory statements to the Discussion.

Response: The Results section has been revised to present only descriptive statistical outputs, including means, ranges, significance levels, variance components, principal component loadings, and correlation coefficients. Interpretative statements and broader implications have been relocated to the Discussion section to maintain proper scientific structure.

Comment 6: Since all entries were obtained from IITA, the "Source" column in Table 1 is unnecessary.

Response: The "Source" column has been removed from Table 1. A clarifying statement has been added in the Materials and Methods section indicating that all hybrids and checks were obtained from the International Institute of Tropical Agriculture (IITA), Ibadan, Nigeria.

Comment 7: Avoid qualitative terms such as "highly significant." Report significance clearly.

Response: The manuscript has been revised to report statistical significance strictly using probability levels ($p < 0.05$, $p < 0.01$, $p < 0.001$), without qualitative modifiers.

Comment 8: Clarify terminology (write terms in full at first use; avoid beginning sentences with acronyms).

Response: All statistical terms are now written in full at first mention, including Genotypic variance, Phenotypic variance, Broad-sense heritability, Genotypic Coefficient of Variation, and Phenotypic Coefficient of Variation. Sentences no longer begin with acronyms, and terminology has been standardized throughout.

Comment 9: Clarify what "Cumulative" refers to in the PCA table.

Response: The PCA table has been revised to read "Cumulative percentage of total variation explained" to improve clarity.

The discussion and conclusion section was also improved.

We are grateful for the reviewer(s)’ detailed and insightful comments. The revisions have strengthened the manuscript by clarifying the FAW damage assessment, correcting an error in the height unit processing, and improving transparency in the statistical modeling framework. We respectfully submit the revised manuscript for further consideration.

Sincerely,

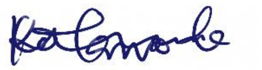

Olayinka A.O

Corresponding Author
