# Supplementary material for: Assessment of agronomic traits in fall armyworm (Spodoptera frugiperda) resistant maize (Zea mays) hybrids grown in the derived savanna agro-ecology of Nigeria
Source: PLoS One. 2026 Jun 26;21(6):e0352140. doi: 10.1371/journal.pone.0352140 (PMC13308806; doi:10.1371/journal.pone.0352140)
Supplement: Attachment — Submitted filename: Response to Reviewers.docx [file pone.0352140.s004.docx]

**Response to Reviewers**

Dear Academic Editor and Reviewer(s),

We sincerely thank you for the evaluation and constructive comments provided on our manuscript titled: Assessment of agronomic traits in fall armyworm (*Spodoptera frugiperda*) resistant maize (*Zea mays*) hybrids grown in the derived savanna agro-ecology of Nigeria.

We have revised the manuscript thoroughly as follows:

**Comment 1: Please specify the type of fall armyworm damage evaluated (leaf, stalk, or ear damage).**

Response: The study evaluated foliar fall armyworm damage (leaf damage) using the modified Davis 1–9 scale at 4, 8, and 12 weeks after planting. This has now been explicitly stated in the Abstract, Materials and Methods (Data Collection section), Results tables, and figure captions to ensure clarity and consistency throughout the manuscript.

**Comment 2: The plant height values appear questionable for tropical hybrids. Please review and correct.**

Response: Upon careful re-examination of the raw dataset and statistical outputs, we identified a unit-of-measurement handling error during statistical processing. Field measurements were correctly recorded in centimeters; however, an error occurred during data handling that affected the reported summaries. This error has now been corrected, and all affected tables and related results have been regenerated using the corrected dataset. The revised plant and ear height values are accurate and internally consistent.

**Comment 3: The reported height of Oba Super II contradicts previous studies.**

Response: Following correction of the unit-processing error described above, all genotype height values including Oba Super II were updated. The corrected values now reflect the properly processed field measurements for the 2024 growing season.

**Comment 4: The ANOVA/mixed model description is not correct. The replicate effect is missing, and the nesting structure should reflect the alpha-lattice design.**

Response: The Statistical Data Analysis section was revised to correctly reflect the alpha-lattice experimental design. Genotype and environment were treated as random effects. Replicates were modeled as nested within environment, and incomplete blocks were modeled as nested within replicate and environment. The genotype × environment interaction was retained within the mixed-model framework. The model description has been rewritten accordingly for accuracy and transparency.

**Comment 5: Avoid interpretation in the Results section. Move explanatory statements to the Discussion.**

Response: The Results section has been revised to present only descriptive statistical outputs, including means, ranges, significance levels, variance components, principal component loadings, and correlation coefficients. Interpretative statements and broader implications have been relocated to the Discussion section to maintain proper scientific structure.

**Comment 6: Since all entries were obtained from IITA, the “Source” column in Table 1 is unnecessary.**

Response: The “Source” column has been removed from Table 1. A clarifying statement has been added in the Materials and Methods section indicating that all hybrids and checks were obtained from the International Institute of Tropical Agriculture (IITA), Ibadan, Nigeria.

**Comment 7: Avoid qualitative terms such as “highly significant.” Report significance clearly.**

Response: The manuscript has been revised to report statistical significance strictly using probability levels (p < 0.05, p < 0.01, p < 0.001), without qualitative modifiers.

**Comment 8: Clarify terminology (write terms in full at first use; avoid beginning sentences with acronyms).**

Response: All statistical terms are now written in full at first mention, including Genotypic variance, Phenotypic variance, Broad-sense heritability, Genotypic Coefficient of Variation, and Phenotypic Coefficient of Variation. Sentences no longer begin with acronyms, and terminology has been standardized throughout.

**Comment 9: Clarify what “Cumulative” refers to in the PCA table.**

Response: The PCA table has been revised to read “Cumulative percentage of total variation explained” to improve clarity.

The discussion and conclusion section was also improved.

We are grateful for the reviewer(s)’ detailed and insightful comments. The revisions have strengthened the manuscript by clarifying the FAW damage assessment, correcting an error in the height unit processing, and improving transparency in the statistical modeling framework. We respectfully submit the revised manuscript for further consideration.

Sincerely,

Olayinka A.O

Corresponding Author
