# Supplementary material for: Assessment of agronomic traits in fall armyworm (Spodoptera frugiperda) resistant maize (Zea mays) hybrids grown in the derived savanna agro-ecology of Nigeria
Source: PLoS One. 2026 Jun 26;21(6):e0352140. doi: 10.1371/journal.pone.0352140 (PMC13308806; doi:10.1371/journal.pone.0352140)
Supplement: Attachment — Submitted filename: Response_to_Reviewers_auresp_2.docx [file pone.0352140.s005.docx]

**Manuscript:** Assessment of agronomic traits in fall armyworm (Spodoptera frugiperda) resistant maize (Zea mays) hybrids grown in the derived savanna agro-ecology of Nigeria **Journal:** PLOS ONE **Manuscript Number:** PONE-D-25-64535R1

Dear Academic Editor and Reviewer(s),

Thank you for your valuable comments. We have carefully addressed all points raised. All changes are shown in the “Revised Manuscript with Track Changes” file.

**Reviewer #1**

**Comment 1:** Which-won-where was wrongly used for genotype-yield trait association.

**Response:** We thank the reviewer for this comment and for the opportunity to clarify. We respectfully point out that the analysis employed in this study is the **Genotype by Yield × Trait (GYT) biplot**, not the GGE biplot. The GYT biplot, as proposed by Yan and Frégeau-Reid (2018), is specifically designed to evaluate and rank genotypes based on their combined performance in grain yield with other desirable agronomic traits simultaneously. Unlike the GGE “which-won-where” polygon view (which identifies winning genotypes for specific environments), the GYT “which-won-where” view identifies which genotypes perform best for specific **yield-trait combinations**.

In this manuscript, the “which-won-where” polygon in Figure 4 correctly shows the sectors in which different genotypes excel for particular trait combinations (e.g., G1 for GY/PASP, GY/EASP, and GY/FFAWD8). This interpretation is fully consistent with the GYT framework and its intended application for multi-trait genotype selection. We believe this clarification and revision fully address the reviewer’s concern while maintaining the methodological integrity of the GYT approach.

For further clarification, kindly check these published articles:

i. Yan, W., & Frégeau-Reid, J. (2018). Genotype by yield*trait (GYT) biplot: A novel approach for genotype selection based on multiple traits. Scientific Reports, 8(1), 8242. <https://doi.org/10.1038/s41598-018-26688-8>
ii. Yan, W., Fregeau-Reid, J., Mountain, N., & Kobler, J. (2019). Genotype and management evaluation based on Genotype by Yield*Trait (GYT) analysis. Crop Breeding, Genetics, and Genomics. 1:e190002. <https://doi.org/10.20900/cbgg20190002>
iii. Mohammadi, R. (2019). Genotype by yield*trait biplot for genotype evaluation and trait profiles in durum wheat. Cereal Research Communications, 47(3), 541-551. <https://doi.org/10.1556/0806.47.2019.32>
iv. Gholizadeh, A., & Ghaffari, M. (2023). Genotype by yield* trait (GYT) biplot analysis: A novel approach for phenotyping sunflower single cross hybrids based on multiple traits. Food Science & Nutrition, 11(10), 5928-5937. <https://doi.org/10.1002/fsn3.3524>

**Comment 2:** The correlation coefficient claimed to have been calculated is erroneous and misleading. Spearman´s correlation coefficient does not determine association among traits; rather, it determines the association between ranks of environments or variables on two data points, which is why it is called Spearman´s Rank Correlation Coefficient (SRCC). Please confirm if it was Spearman's or Pearson´s correlation coefficient you used.

**Response:** Thank you for this important observation. We carefully reviewed our correlation analysis again. We originally labelled it as Spearman’s, but after checking the actual calculation, we confirmed it was **Pearson’s correlation coefficient (r)**. We have now changed it to Pearson’s throughout the manuscript. Pearson’s is the correct and more suitable method here because it measures the strength and direction of the linear relationship between the actual trait values. This matches the reviewer’s view and is appropriate for this study. We apologize for the earlier error. The Methods section now clearly states: “Pearson’s correlation analysis was computed to determine the strength and direction of associations among the measured traits using the ‘metan’ package in R version 4.4.2.”

**Comment 3:** Other comments and corrections are in the body of the manuscript.

**Response:** We have addressed all comments and corrections marked in the body of the manuscript. The main changes include:

**Weather figure (Fig. 1):** We reviewed the reviewer’s concern about providing the weather figure for the Oyo experimental site. We completely removed the weather figure because we couldn’t get the data. However, both experimental sites are located within the same derived savanna agro-ecological zone and share similar climatic conditions (bimodal rainfall of 1,000-1,200 mm and mean temperatures of 28-30 °C). This avoids presenting incomplete data while still providing the necessary environmental information.

Sincerely,

Olayinka A.O

Corresponding Author
Email: [aokolawole@lautech.edu.ng](mailto:aokolawole@lautech.edu.ng)
