# Supplementary material for: Assessment of agronomic traits in fall armyworm (Spodoptera frugiperda) resistant maize (Zea mays) hybrids grown in the derived savanna agro-ecology of Nigeria
Source: PLoS One. 2026 Jun 26;21(6):e0352140. doi: 10.1371/journal.pone.0352140 (PMC13308806; doi:10.1371/journal.pone.0352140)
Supplement: Peer review history

# Peer review: Decision Letter 0

**DOI:** 10.1371/journal.pone.0352140.r001

**Authors:** Tzen-Yuh Chiang

8 Jan 2026

PONE-D-25-64535

Assessment of agronomic traits in fall armyworm (Spodoptera frugiperda) resisistant maize hybrids grown in the derived savanna agro-ecology of Nigeria

PLOS One

Dear Dr. Olayinka,

Thank you for submitting your manuscript to PLOS ONE. After careful consideration, we feel that it has merit but does not fully meet PLOS ONE’s publication criteria as it currently stands. Therefore, we invite you to submit a revised version of the manuscript that addresses the points raised during the review process.

Please submit your revised manuscript by Feb 22 2026 11:59PM. If you will need more time than this to complete your revisions, please reply to this message or contact the journal office at plosone@plos.org. When you're ready to submit your revision, log on to https://www.editorialmanager.com/pone/ and select the 'Submissions Needing Revision' folder to locate your manuscript file.

Please include the following items when submitting your revised manuscript:

- A letter that responds to each point raised by the academic editor and reviewer(s). You should upload this letter as a separate file labeled 'Response to Reviewers'.
- A marked-up copy of your manuscript that highlights changes made to the original version. You should upload this as a separate file labeled 'Revised Manuscript with Track Changes'.
- An unmarked version of your revised paper without tracked changes. You should upload this as a separate file labeled 'Manuscript'.

If you would like to make changes to your financial disclosure, please include your updated statement in your cover letter. Guidelines for resubmitting your figure files are available below the reviewer comments at the end of this letter.

If applicable, we recommend that you deposit your laboratory protocols in protocols.io to enhance the reproducibility of your results. Protocols.io assigns your protocol its own identifier (DOI) so that it can be cited independently in the future. For instructions see: https://journals.plos.org/plosone/s/submission-guidelines#loc-laboratory-protocols. Additionally, PLOS ONE offers an option for publishing peer-reviewed Lab Protocol articles, which describe protocols hosted on protocols.io. Read more information on sharing protocols at https://plos.org/protocols?utm_medium=editorial-email&utm_source=authorletters&utm_campaign=protocols.

We look forward to receiving your revised manuscript.

Kind regards,

Tzen-Yuh Chiang

Academic Editor

PLOS One

Journal Requirements:

When submitting your revision, we need you to address these additional requirements.

1. Please ensure that your manuscript meets PLOS ONE's style requirements, including those for file naming. The PLOS ONE style templates can be found at

https://journals.plos.org/plosone/s/file?id=wjVg/PLOSOne_formatting_sample_main_body.pdf and

https://journals.plos.org/plosone/s/file?id=ba62/PLOSOne_formatting_sample_title_authors_affiliations.pdf

2. Please note that PLOS One has specific guidelines on code sharing for submissions in which author-generated code underpins the findings in the manuscript. In these cases, we expect all author-generated code to be made available without restrictions upon publication of the work. Please review our guidelines at https://journals.plos.org/plosone/s/materials-and-software-sharing#loc-sharing-code and ensure that your code is shared in a way that follows best practice and facilitates reproducibility and reuse.

3. In the online submission form, you indicated that “The data underlying the results presented in the study are available on request to the corresponding Author”

All PLOS journals now require all data underlying the findings described in their manuscript to be freely available to other researchers, either 1. In a public repository, 2. Within the manuscript itself, or 3. Uploaded as supplementary information.

This policy applies to all data except where public deposition would breach compliance with the protocol approved by your research ethics board. If your data cannot be made publicly available for ethical or legal reasons (e.g., public availability would compromise patient privacy), please explain your reasons on resubmission and your exemption request will be escalated for approval.

4. PLOS requires an ORCID iD for the corresponding author in Editorial Manager on papers submitted after December 6th, 2016. Please ensure that you have an ORCID iD and that it is validated in Editorial Manager. To do this, go to ‘Update my Information’ (in the upper left-hand corner of the main menu), and click on the Fetch/Validate link next to the ORCID field. This will take you to the ORCID site and allow you to create a new iD or authenticate a pre-existing iD in Editorial Manager.

5. If the reviewer comments include a recommendation to cite specific previously published works, please review and evaluate these publications to determine whether they are relevant and should be cited. There is no requirement to cite these works unless the editor has indicated otherwise.

[Note: HTML markup is below. Please do not edit.]

Reviewers' comments:

Reviewer's Responses to Questions

**Comments to the Author**

1. Is the manuscript technically sound, and do the data support the conclusions?

The manuscript must describe a technically sound piece of scientific research with data that supports the conclusions. Experiments must have been conducted rigorously, with appropriate controls, replication, and sample sizes. The conclusions must be drawn appropriately based on the data presented.

Reviewer #1: Partly

**********

2. Has the statistical analysis been performed appropriately and rigorously?

Reviewer #1: Yes

**********

3. Have the authors made all data underlying the findings in their manuscript fully available?

The PLOS Data policy requires authors to make all data underlying the findings described in their manuscript fully available without restriction, with rare exception (please refer to the Data Availability Statement in the manuscript PDF file). The data should be provided as part of the manuscript or its supporting information, or deposited to a public repository. For example, in addition to summary statistics, the data points behind means, medians and variance measures should be available. If there are restrictions on publicly sharing data—e.g. participant privacy or use of data from a third party—those must be specified.

Reviewer #1: Yes

**********

4. Is the manuscript presented in an intelligible fashion and written in standard English?

PLOS ONE does not copyedit accepted manuscripts, so the language in submitted articles must be clear, correct, and unambiguous. Any typographical or grammatical errors should be corrected at revision, so please note any specific errors here.

Reviewer #1: No

**********

5. Review Comments to the Author

Please use the space provided to explain your answers to the questions above. You may also include additional comments for the author, including concerns about dual publication, research ethics, or publication ethics. (Please upload your review as an attachment if it exceeds 20,000 characters)

Reviewer #1: 1. The Plant height for tropical maize hybrids reported in this manuscript is misleading compared to most similar studies. Please review and correct.

2. Fall armyworm damage was used loosely without specifying the type of fall armyworm damage, whether leaf damage, stalk damage or ear damage.

3. In several parts of the results, the authors mixed results and discussion.

4. The grammar is poor as there are inconsistencies in the use of tenses: past and present tenses, especially in the discussion session.

5. All my comments and corrections are in the body of the MS.

**********

6. PLOS authors have the option to publish the peer review history of their article (what does this mean?). If published, this will include your full peer review and any attached files.

If you choose “no”, your identity will remain anonymous but your review may still be made public.

**Do you want your identity to be public for this peer review?**   For information about this choice, including consent withdrawal, please see our Privacy Policy.

Reviewer #1: No

**********

[NOTE: If reviewer comments were submitted as an attachment file, they will be attached to this email and accessible via the submission site. Please log into your account, locate the manuscript record, and check for the action link "View Attachments". If this link does not appear, there are no attachment files.]

To ensure your figures meet our technical requirements, please review our figure guidelines: https://journals.plos.org/plosone/s/figures

You may also use PLOS’s free figure tool, NAAS, to help you prepare publication quality figures: https://journals.plos.org/plosone/s/figures#loc-tools-for-figure-preparation.

NAAS will assess whether your figures meet our technical requirements by comparing each figure against our figure specifications.

Attachment Submitted filename: PONE-D-25-64535_REVIEWED.pdf

# Author comment: Author response to Decision Letter 1

**DOI:** 10.1371/journal.pone.0352140.r002

2 Mar 2026

We have revised the manuscript thoroughly as follows:

Comment 1: Please specify the type of fall armyworm damage evaluated (leaf, stalk, or ear damage).

Response: The study evaluated foliar fall armyworm damage (leaf damage) using the modified Davis 1–9 scale at 4, 8, and 12 weeks after planting. This has now been explicitly stated in the Abstract, Materials and Methods (Data Collection section), Results tables, and figure captions to ensure clarity and consistency throughout the manuscript.

Comment 2: The plant height values appear questionable for tropical hybrids. Please review and correct.

Response: Upon careful re-examination of the raw dataset and statistical outputs, we identified a unit-of-measurement handling error during statistical processing. Field measurements were correctly recorded in centimeters; however, an error occurred during data handling that affected the reported summaries. This error has now been corrected, and all affected tables and related results have been regenerated using the corrected dataset. The revised plant and ear height values are accurate and internally consistent.

Comment 3: The reported height of Oba Super II contradicts previous studies.

Response: Following correction of the unit-processing error described above, all genotype height values including Oba Super II were updated. The corrected values now reflect the properly processed field measurements for the 2024 growing season.

Comment 4: The ANOVA/mixed model description is not correct. The replicate effect is missing, and the nesting structure should reflect the alpha-lattice design.

Response: The Statistical Data Analysis section was revised to correctly reflect the alpha-lattice experimental design. Genotype and environment were treated as random effects. Replicates were modeled as nested within environment, and incomplete blocks were modeled as nested within replicate and environment. The genotype × environment interaction was retained within the mixed-model framework. The model description has been rewritten accordingly for accuracy and transparency.

Comment 5: Avoid interpretation in the Results section. Move explanatory statements to the Discussion.

Response: The Results section has been revised to present only descriptive statistical outputs, including means, ranges, significance levels, variance components, principal component loadings, and correlation coefficients. Interpretative statements and broader implications have been relocated to the Discussion section to maintain proper scientific structure.

Comment 6: Since all entries were obtained from IITA, the “Source” column in Table 1 is unnecessary.

Response: The “Source” column has been removed from Table 1. A clarifying statement has been added in the Materials and Methods section indicating that all hybrids and checks were obtained from the International Institute of Tropical Agriculture (IITA), Ibadan, Nigeria.

Comment 7: Avoid qualitative terms such as “highly significant.” Report significance clearly.

Response: The manuscript has been revised to report statistical significance strictly using probability levels (p < 0.05, p < 0.01, p < 0.001), without qualitative modifiers.

Comment 8: Clarify terminology (write terms in full at first use; avoid beginning sentences with acronyms).

Response: All statistical terms are now written in full at first mention, including Genotypic variance, Phenotypic variance, Broad-sense heritability, Genotypic Coefficient of Variation, and Phenotypic Coefficient of Variation. Sentences no longer begin with acronyms, and terminology has been standardized throughout.

Comment 9: Clarify what “Cumulative” refers to in the PCA table.

Response: The PCA table has been revised to read “Cumulative percentage of total variation explained” to improve clarity.

The discussion and conclusion section was also revised

Attachment Submitted filename: Response to Reviewers.docx

# Peer review: Decision Letter 1

**DOI:** 10.1371/journal.pone.0352140.r003

**Authors:** Tzen-Yuh Chiang, Tzen-Yuh Chiang

15 Apr 2026

<div>PONE-D-25-64535R1

Assessment of agronomic traits in fall armyworm (Spodoptera frugiperda) resisistant maize (Zea mays) hybrids grown in the derived savanna agro-ecology of Nigeria

PLOS One

Dear Dr.  Olayinka,

Thank you for submitting your manuscript to PLOS ONE. After careful consideration, we feel that it has merit but does not fully meet PLOS ONE’s publication criteria as it currently stands. Therefore, we invite you to submit a revised version of the manuscript that addresses the points raised during the review process.

Please submit your revised manuscript by May 30 2026 11:59PM. If you will need more time than this to complete your revisions, please reply to this message or contact the journal office at plosone@plos.org. When you're ready to submit your revision, log on to https://www.editorialmanager.com/pone/ and select the 'Submissions Needing Revision' folder to locate your manuscript file.

Please include the following items when submitting your revised manuscript:

- A letter that responds to each point raised by the academic editor and reviewer(s). You should upload this letter as a separate file labeled 'Response to Reviewers'.
- A marked-up copy of your manuscript that highlights changes made to the original version. You should upload this as a separate file labeled 'Revised Manuscript with Track Changes'.
- An unmarked version of your revised paper without tracked changes. You should upload this as a separate file labeled 'Manuscript'.

If you would like to make changes to your financial disclosure, please include your updated statement in your cover letter. Guidelines for resubmitting your figure files are available below the reviewer comments at the end of this letter.

If applicable, we recommend that you deposit your laboratory protocols in protocols.io to enhance the reproducibility of your results. Protocols.io assigns your protocol its own identifier (DOI) so that it can be cited independently in the future. For instructions see: https://journals.plos.org/plosone/s/submission-guidelines#loc-laboratory-protocols. Additionally, PLOS ONE offers an option for publishing peer-reviewed Lab Protocol articles, which describe protocols hosted on protocols.io. Read more information on sharing protocols at https://plos.org/protocols?utm_medium=editorial-email&utm_source=authorletters&utm_campaign=protocols.

As the corresponding author, your ORCID iD is verified in the submission system and will appear in the published article. PLOS supports the use of ORCID, and we encourage all coauthors to register for an ORCID iD and use it as well. Please encourage your coauthors to verify their ORCID iD within the submission system before final acceptance, as unverified ORCID iDs will not appear in the published article. *Only*   the individual author can complete the verification step; PLOS staff *cannot*   verify ORCID iDs on behalf of authors.

We look forward to receiving your revised manuscript.

Kind regards,

Tzen-Yuh Chiang

Academic Editor

PLOS One

Journal Requirements:

If the reviewer comments include a recommendation to cite specific previously published works, please review and evaluate these publications to determine whether they are relevant and should be cited. There is no requirement to cite these works unless the editor has indicated otherwise.

Please review your reference list to ensure that it is complete and correct. If you have cited papers that have been retracted, please include the rationale for doing so in the manuscript text, or remove these references and replace them with relevant current references. Any changes to the reference list should be mentioned in the rebuttal letter that accompanies your revised manuscript. If you need to cite a retracted article, indicate the article’s retracted status in the References list and also include a citation and full reference for the retraction notice.

[Note: HTML markup is below. Please do not edit.]

Reviewers' comments:

Reviewer's Responses to Questions

**Comments to the Author**

1. If the authors have adequately addressed your comments raised in a previous round of review and you feel that this manuscript is now acceptable for publication, you may indicate that here to bypass the “Comments to the Author” section, enter your conflict of interest statement in the “Confidential to Editor” section, and submit your "Accept" recommendation.

Reviewer #1: (No Response)

**********

2. Is the manuscript technically sound, and do the data support the conclusions?

The manuscript must describe a technically sound piece of scientific research with data that supports the conclusions. Experiments must have been conducted rigorously, with appropriate controls, replication, and sample sizes. The conclusions must be drawn appropriately based on the data presented.

Reviewer #1: Partly

**********

3. Has the statistical analysis been performed appropriately and rigorously?

Reviewer #1: No

**********

4. Have the authors made all data underlying the findings in their manuscript fully available?

The PLOS Data policy requires authors to make all data underlying the findings described in their manuscript fully available without restriction, with rare exception (please refer to the Data Availability Statement in the manuscript PDF file). The data should be provided as part of the manuscript or its supporting information, or deposited to a public repository. For example, in addition to summary statistics, the data points behind means, medians and variance measures should be available. If there are restrictions on publicly sharing data—e.g. participant privacy or use of data from a third party—those must be specified.

Reviewer #1: Yes

**********

5. Is the manuscript presented in an intelligible fashion and written in standard English?

PLOS ONE does not copyedit accepted manuscripts, so the language in submitted articles must be clear, correct, and unambiguous. Any typographical or grammatical errors should be corrected at revision, so please note any specific errors here.

Reviewer #1: No

**********

6. Review Comments to the Author

Please use the space provided to explain your answers to the questions above. You may also include additional comments for the author, including concerns about dual publication, research ethics, or publication ethics. (Please upload your review as an attachment if it exceeds 20,000 characters)

Reviewer #1: 1. Which-won-where was wrongly used for genotype-yield trait association

2. The correlation coefficient claimed to have been calculated is erroneous and misleading. Spearman´s correlation coefficient does not determine association among traits; rather, it determines the association between ranks of environments or variables on two data points, which is why it is called Spearman´s Rank Correlation Coefficient (SRCC). Please confirm if it was Spearman's or Pearson´s correlation coefficient you used.

3. Other comments and corrections are in the body of the manuscript.

**********

7. PLOS authors have the option to publish the peer review history of their article (what does this mean?). If published, this will include your full peer review and any attached files.

If you choose “no”, your identity will remain anonymous but your review may still be made public.

**Do you want your identity to be public for this peer review?**   For information about this choice, including consent withdrawal, please see our Privacy Policy.

Reviewer #1: No

**********

[NOTE: If reviewer comments were submitted as an attachment file, they will be attached to this email and accessible via the submission site. Please log into your account, locate the manuscript record, and check for the action link "View Attachments". If this link does not appear, there are no attachment files.]

To ensure your figures meet our technical requirements, please review our figure guidelines: https://journals.plos.org/plosone/s/figures

You may also use PLOS’s free figure tool, NAAS, to help you prepare publication quality figures: https://journals.plos.org/plosone/s/figures#loc-tools-for-figure-preparation.

NAAS will assess whether your figures meet our technical requirements by comparing each figure against our figure specifications.

Attachment Submitted filename: PONE-D-25-64535_R1_REVISION.pdf

# Author comment: Author response to Decision Letter 2

**DOI:** 10.1371/journal.pone.0352140.r004

22 May 2026

Dear Academic Editor and Reviewer(s),

Thank you for your valuable comments. We have carefully addressed all points raised. All changes are shown in the “Revised Manuscript with Track Changes” file.

Reviewer #1

Comment 1: Which-won-where was wrongly used for genotype-yield trait association.

Response: We thank the reviewer for this comment and for the opportunity to clarify. We respectfully point out that the analysis employed in this study is the Genotype by Yield × Trait (GYT) biplot, not the GGE biplot. The GYT biplot, as proposed by Yan and Frégeau-Reid (2018), is specifically designed to evaluate and rank genotypes based on their combined performance in grain yield with other desirable agronomic traits simultaneously. Unlike the GGE “which-won-where” polygon view (which identifies winning genotypes for specific environments), the GYT “which-won-where” view identifies which genotypes perform best for specific yield-trait combinations.

In this manuscript, the “which-won-where” polygon in Figure 4 correctly shows the sectors in which different genotypes excel for particular trait combinations (e.g., G1 for GY/PASP, GY/EASP, and GY/FFAWD8). This interpretation is fully consistent with the GYT framework and its intended application for multi-trait genotype selection. We believe this clarification and revision fully address the reviewer’s concern while maintaining the methodological integrity of the GYT approach.

For further clarification, kindly check these published articles:

i. Yan, W., & Frégeau-Reid, J. (2018). Genotype by yield*trait (GYT) biplot: A novel approach for genotype selection based on multiple traits. Scientific Reports, 8(1), 8242. https://doi.org/10.1038/s41598-018-26688-8

ii. Yan, W., Fregeau-Reid, J., Mountain, N., & Kobler, J. (2019). Genotype and management evaluation based on Genotype by Yield*Trait (GYT) analysis. Crop Breeding, Genetics, and Genomics. 1:e190002. https://doi.org/10.20900/cbgg20190002

iii. Mohammadi, R. (2019). Genotype by yield*trait biplot for genotype evaluation and trait profiles in durum wheat. Cereal Research Communications, 47(3), 541-551. https://doi.org/10.1556/0806.47.2019.32

iv. Gholizadeh, A., & Ghaffari, M. (2023). Genotype by yield* trait (GYT) biplot analysis: A novel approach for phenotyping sunflower single cross hybrids based on multiple traits. Food Science & Nutrition, 11(10), 5928-5937. https://doi.org/10.1002/fsn3.3524

Comment 2: The correlation coefficient claimed to have been calculated is erroneous and misleading. Spearman´s correlation coefficient does not determine association among traits; rather, it determines the association between ranks of environments or variables on two data points, which is why it is called Spearman´s Rank Correlation Coefficient (SRCC). Please confirm if it was Spearman's or Pearson´s correlation coefficient you used.

Response: Thank you for this important observation. We carefully reviewed our correlation analysis again. We originally labelled it as Spearman’s, but after checking the actual calculation, we confirmed it was Pearson’s correlation coefficient (r). We have now changed it to Pearson’s throughout the manuscript. Pearson’s is the correct and more suitable method here because it measures the strength and direction of the linear relationship between the actual trait values. This matches the reviewer’s view and is appropriate for this study. We apologize for the earlier error. The Methods section now clearly states: “Pearson’s correlation analysis was computed to determine the strength and direction of associations among the measured traits using the ‘metan’ package in R version 4.4.2.”

Comment 3: Other comments and corrections are in the body of the manuscript.

Response: We have addressed all comments and corrections marked in the body of the manuscript. The main changes include:

Weather figure (Fig. 1): We reviewed the reviewer’s concern about providing the weather figure for the Oyo experimental site. We completely removed the weather figure because we couldn’t get the data. However, both experimental sites are located within the same derived savanna agro-ecological zone and share similar climatic conditions (bimodal rainfall of 1,000-1,200 mm and mean temperatures of 28-30 °C). This avoids presenting incomplete data while still providing the necessary environmental information.

Attachment Submitted filename: Response_to_Reviewers_auresp_2.docx

# Peer review: Decision Letter 2

**DOI:** 10.1371/journal.pone.0352140.r005

**Authors:** Tzen-Yuh Chiang, Tzen-Yuh Chiang, Tzen-Yuh Chiang

8 Jun 2026

Assessment of agronomic traits in fall armyworm (Spodoptera frugiperda) resisistant maize (Zea mays) hybrids grown in the derived savanna agro-ecology of Nigeria

PONE-D-25-64535R2

Dear Dr. Olayinka,

We’re pleased to inform you that your manuscript has been judged scientifically suitable for publication and will be formally accepted for publication once it meets all outstanding technical requirements.

Within one week, you’ll receive an e-mail detailing the required amendments. When these have been addressed, you’ll receive a formal acceptance letter and your manuscript will be scheduled for publication.

An invoice will be generated when your article is formally accepted. Please note, if your institution has a publishing partnership with PLOS and your article meets the relevant criteria, all or part of your publication costs will be covered. Please make sure your user information is up-to-date by logging into Editorial Manager at Editorial Manager® and clicking the ‘Update My Information' link at the top of the page. For questions related to billing, please contact billing support.

If your institution or institutions have a press office, please notify them about your upcoming paper to help maximize its impact. If they’ll be preparing press materials, please inform our press team as soon as possible -- no later than 48 hours after receiving the formal acceptance. Your manuscript will remain under strict press embargo until 2 pm Eastern Time on the date of publication. For more information, please contact onepress@plos.org.

Kind regards,

Tzen-Yuh Chiang

Academic Editor

PLOS One

Additional Editor Comments (optional):

Reviewers' comments:

Reviewer's Responses to Questions

**Comments to the Author**

1. If the authors have adequately addressed your comments raised in a previous round of review and you feel that this manuscript is now acceptable for publication, you may indicate that here to bypass the “Comments to the Author” section, enter your conflict of interest statement in the “Confidential to Editor” section, and submit your "Accept" recommendation.

Reviewer #1: All comments have been addressed

**********

2. Is the manuscript technically sound, and do the data support the conclusions?

The manuscript must describe a technically sound piece of scientific research with data that supports the conclusions. Experiments must have been conducted rigorously, with appropriate controls, replication, and sample sizes. The conclusions must be drawn appropriately based on the data presented.

Reviewer #1: Yes

**********

3. Has the statistical analysis been performed appropriately and rigorously?

Reviewer #1: Yes

**********

4. Have the authors made all data underlying the findings in their manuscript fully available?

The PLOS Data policy requires authors to make all data underlying the findings described in their manuscript fully available without restriction, with rare exception (please refer to the Data Availability Statement in the manuscript PDF file). The data should be provided as part of the manuscript or its supporting information, or deposited to a public repository. For example, in addition to summary statistics, the data points behind means, medians and variance measures should be available. If there are restrictions on publicly sharing data—e.g. participant privacy or use of data from a third party—those must be specified.

Reviewer #1: Yes

**********

5. Is the manuscript presented in an intelligible fashion and written in standard English?

PLOS ONE does not copyedit accepted manuscripts, so the language in submitted articles must be clear, correct, and unambiguous. Any typographical or grammatical errors should be corrected at revision, so please note any specific errors here.

Reviewer #1: Yes

**********

6. Review Comments to the Author

Please use the space provided to explain your answers to the questions above. You may also include additional comments for the author, including concerns about dual publication, research ethics, or publication ethics. (Please upload your review as an attachment if it exceeds 20,000 characters)

Reviewer #1: The authors have responded to the issues raised in the manuscript. The Corrections and clarifications are satisfactory

**********

7. PLOS authors have the option to publish the peer review history of their article (what does this mean?). If published, this will include your full peer review and any attached files.

If you choose “no”, your identity will remain anonymous but your review may still be made public.

**Do you want your identity to be public for this peer review?**   For information about this choice, including consent withdrawal, please see our Privacy Policy.

Reviewer #1: No

**********

# Editor report: Acceptance letter

**DOI:** 10.1371/journal.pone.0352140.r006

**Authors:** Tzen-Yuh Chiang, Tzen-Yuh Chiang, Tzen-Yuh Chiang

PONE-D-25-64535R2

PLOS One

Dear Dr. Olayinka,

I'm pleased to inform you that your manuscript has been deemed suitable for publication in PLOS One. Congratulations! Your manuscript is now being handed over to our production team.

At this stage, our production department will prepare your paper for publication. This includes ensuring the following:

* All references, tables, and figures are properly cited

* All relevant supporting information is included in the manuscript submission,

* There are no issues that prevent the paper from being properly typeset

You will receive further instructions from the production team, including instructions on how to review your proof when it is ready. Please keep in mind that we are working through a large volume of accepted articles, so please give us a few days to review your paper and let you know the next and final steps.

Lastly, if your institution or institutions have a press office, please let them know about your upcoming paper now to help maximize its impact. If they'll be preparing press materials, please inform our press team within the next 48 hours. Your manuscript will remain under strict press embargo until 2 pm Eastern Time on the date of publication. For more information, please contact onepress@plos.org.

You will receive an invoice from PLOS for your publication fee after your manuscript has reached the completed accept phase. If you receive an email requesting payment before acceptance or for any other service, this may be a phishing scheme. Learn how to identify phishing emails and protect your accounts at https://explore.plos.org/phishing.

If we can help with anything else, please email us at customercare@plos.org.

Thank you for submitting your work to PLOS ONE and supporting open access.

Kind regards,

PLOS ONE Editorial Office Staff

on behalf of

Dr. Tzen-Yuh Chiang

Academic Editor

PLOS One
